# Exploring the prognostic value of S100A11 and its association with immune infiltration in breast cancer

**DOI:** 10.1038/s41598-023-50160-x

**Published:** 2023-12-21

**Authors:** Junfang He, Yuxi Lei, Xiabin Li, Bin Wu, Yan Tang

**Affiliations:** 1 School of Public Health, Southwest Medical University, Luzhou, 646000 Sichuan China; 2 Precision Pathology Diagnosis for Serious Diseases Key Laboratory of LuZhou, Luzhou, 646000 Sichuan China; 3 Departments of Breast Surgery, The Affiliated Hospital of Southwest Medical University, Luzhou, 646000 Sichuan China; 4 Institute of Cancer Medicine, School of Basic Medical Sciences, Southwest Medical University, Luzhou, 646000 Sichuan China

**Keywords:** Cancer, Computational biology and bioinformatics, Biomarkers, Risk factors

## Abstract

Breast cancer (BC) is a severe danger to women’s lives and health globally. S100A11 is aberrantly expressed in many carcinomas and serves a crucial function in cancer development. However, the role of S100A11 in BC is unclear. In this study, we utilized multiple databases and online tools, including the TCGA database, cBioPortal, and STRING, to evaluate the significance of S100A11 in BC prognosis and immune infiltration. We found that S100A11 was considerably more abundant in BC tissues. Survival analysis indicated that individuals with S100A11 high expression of BC had shorter overall survival. Multivariate Cox regression analysis revealed that high S100A11 expression independently influenced the poor outcome of patients with BC (***HR*** = 1.738, *95%CI* 1.197–2.524). Our nomogram incorporating five factors, including S100A11, age, clinical stage, N, and M, was developed to anticipate the survival probability in BC prognosis. The model demonstrated good consistency and accuracy. Furthermore, the mutation rete of S100A11 was 14%. Survival analysis suggested that breast cancer patients with S100A11 mutation had a worse prognosis. KEGG pathway enrichment analysis revealed that S100A11 may be mainly involved in the IL-17 signaling pathway. Finally, we discovered a correlation between S100A11 expression and immune cell infiltration on BC. S100A11 expression was positively associated with 17 immune checkpoint-related genes. In conclusion, this study indicates that S100A11 may contribute to a worse prognosis for BC and potentially has a significant impact through its influence on immune cell infiltration and the IL-17 signaling pathway.

## Introduction

Breast cancer (BC) is a particularly frequent malignant carcinoma in women globally. According to global statistics for 2022, BC is the most frequently diagnosed tumor, with an anticipated 20,000 new cases, which accounts for approximately 3.11% of all cancer cases and approximately 36.85 million deaths from this disease^1^. In 2070, the number of patients with BC will have approached 4.4 million^2^. Despite the abundance of treatment options for breast cancer patients, approximately 12% of patients with BC still metastasize each year; additionally, the 5-year survival rate is only 26%^3^. Therefore, it is imperative to identify and validate newer biomarkers that can effectively predict the progression and prognosis of breast cancer. Furthermore, gaining a comprehensive understanding of the underlying mechanisms is crucial in order to enhance the prognosis and overall outcomes for BC patients.

Calcium ions are a critical factor in controlling the equilibrium of signal transduction, cell survival, and metabolism. Furthermore, an imbalance of calcium ions has been directly linked to the development of tumors^4^. The S100 family (S100s), consisting of at least 20 members, is a group of calcium-binding proteins. These proteins are involved in regulating calcium ions and interacting with target proteins to participate in many different types of cellular activities, including cell survival, cell differentiation, cell proliferation, and cell motility. Numerous members of the S100 family show abnormal expression patterns in various kinds of malignancies. These members play a role in regulating cancer cell proliferation, migration, and differentiation. These processes, in turn, have a significant impact on tumorigenesis and cancer development^5^. S100A11, also named S100C, is an individual of the S100 family, which is found in the q21 region of human chromosome 1, and it could engage in a number of biological activities that include enzyme activity, cell growth, apoptosis, and inflammatory responses^6^. S100A11 is abnormally expressed in a large number of malignancies and could engage in the development and progression of cancers. Zeng et al.^7^, for instance, found that S100A11 was overexpressed in pancreatic ductal adenocarcinoma (PDAC) and had a strong link with a poor prognosis and disease progression in patients, which mechanistically demonstrated that S100A11 could induce malignant biological behaviors, including proliferation and migration of PDAC cells, by activating the pentose phosphate pathway. In intrahepatic cholangiocarcinoma, S100A11 had the potential to promote cancer cell proliferation by regulating the P38/MAPK signaling pathway^8^. Liu et al.^9^ discovered that S100A11 was excessively expressed in BC tissues, and its subcellular localization was in the cytoplasm, proving that S100A11 is a unique diagnostic biomarker for BC. Based on an exploration of several online databases, Zhang et al. identified a close correlation between higher expression of S100A11 mRNA and adverse outcomes in patients with BC^10^. However, there is a lack of clarity regarding the mechanism of S100A11’s role in the development of BC and its influence on the immune microenvironment in BC.

This study aimed to assess the expression, prognosis, and mutation patterns of S100A11 in breast cancer, as well as its interaction network, immune cell infiltration, and immune checkpoints. We utilized data from the TCGA database, cBioPortal, STRING, and other databases and tools. Cox regression analysis and a nomogram were employed to assess the value of S100A11 in predicting the prognosis of BC and the potential mechanism of action. This research is intended to provide valuable insights for future research on the clinical treatment of breast cancer.

## Data sources and methods

### Data acquisition and processing

From the Cancer Genome Atlas(TCGA)database(https://portal.gdc.com), RNA-sequencing data for breast cancer (HTseq-TPM) and relevant clinical data for patients with BC were obtained. To improve the accuracy of this study, patients with complete clinicopathologic information (age, gender, stage, T(Tumor), N(regional lymph nodes), M (distant metastases), overall survival, and survival status) were included, while patients with clinical data lacking overall survival (OS) or OS < 30 days were excluded. Finally, 874 BC patients had complete clinical data. At the same time, we obtained the RNA-seq data of breast normal tissue (179) from the Genotype-Tissue Expression(GTEX)dataset from the UCSC Xena database (https://xena.ucsc.edu/). Before further analysis, the RNA sequencing data were log2 (TPM + 1) transformed.

### Levels of S100A11 mRNA as well as protein expression in BC

Based on RNA-seq data for BC and normal breast tissues from the TCGA database and GTEX, we utilized the limma package in R software to assess the expression level of the S100A11 mRNA in BC tissues. Furthermore, the ggplot2 package was utilized to visualize these analysis results.

The Human Protein Atls(HPA) database (https://www.proteinatlas.org) is an online tool with proteomics, transcriptomics, and system biology data, which also includes protein expression data and clinical information for both normal and tumor tissues^11^. We obtained S100A11 protein information in BC tissues as well as normal breast tissues from this database.

### Analysis of the connection between S100A11 and clinical features of BC patients

In R software, the limma package was employed to examine the connection between S100A11 expression levels and clinicopathological characteristics (including age, clinical stage, T, M, and N). Meanwhile, the “ggpubr” package was utilized to draw box plots to visualize the results of this analysis.

### Prognostic value of S100A11 in BC

In order to assess the connection between S100A11 and the OS of patients with BC, we used the survminer package in R software. Specifically, we applied the sur_cutpoint algorithm to determine the best cutoff value of S100A11 expression. Furthermore, we employed the Kaplan–Meier method to draw a survival curve based on this cutoff value. Finally, we performed both univariate and multivariate Cox regression analyses separately for the S100A11 high- and low-expression groups.

### Establishment and verification of a Nomogram to anticipate prognosis in BC patients

Following that, a nomogram for prognostic prediction was created based on S100A11 (high- and low-expression), age, stage, N, and M. To evaluate the model’s predictive accuracy and stability, the C-index, time-dependent ROC curves, and calibration curves were utilized, respectively. For this portion of the study, we utilized the rms package and performed Cox regression analysis in R software. The criteria for interpreting the C-index values are as follows: 0.50–0.70 indicates poor accuracy; 0.70–0.90 indicates moderate accuracy; > 0.90 indicates excellent accuracy.

### Mutation level of S100A11 and its relationship with the prognosis of patients with BC

cBioPortal (https://www.cbioportal.org) is a complete free website that integrates data mining, datasets, and visualization based on the TCGA database^12^. Based on cBioPortal, we utilized breast cancer samples (TCGA, The Pan-Cancer Atlas) to obtain S100A11 mutations and genetic variants. Setup requirements: mRNA expression was z-scored relative to all samples (log RNA Seq V2 RSEM), and the z-score threshold was set to ± 2. Using this tool, we also investigated the link between S100A11 mutation levels and the BC prognosis, including progress-free survival (PFS), disease-specific survival (DSS), OS, and disease-free survival (DFS).

### Protein–protein interaction(PPI) networks and biological functions enrichment analysis of S100A11-related genes in BC

We utilized cor.test() in R software to obtain genes co-expressed with S100A11 based on RNA-sequencing data of BC from the TCGA database. Correlation coefficients >|0.3| as well as *P* < 0.05 were considered relevant.

Next, in R software, the limma package was utilized to obtain the differential genes (DEGs) between S100A11 high- and low-expression groups. FDR < 0.05 and |log 2FC|> 1.5 were considered S100A11-related DEGs. Subsequently, we performed an intersection analysis to identify common genes by comparing the DEGs associated with S100A11 to the genes that are co-expressed with S100A11. These common genes were then subjected to enrichment analysis to determine their functions.

Additionally, STRING (https://string-db.org/) is a searchable resource that is utilized to create protein–protein interaction (PPI) networks^13^. We used this database to construct a PPI network using the common genes from S100A11-related DEGs and genes co-expressed with S100A11. The aim was to identify proteins that interact with S100A11. Cytoscape software (v3.9.0) was utilized to visualize the findings.

The clusterProfiler package in R language software was utilized to carry out the Gene Ontology (GO) and Kyoto Encyclopedia of Genes and Genomes (KEGG) pathway enrichment analysis on S100A11-associated genes. The GO analysis included three categories: biological process (BP), molecular function (MF), and cellular component (CC). In our study, it was considered statistically significant at *P* < 0.05.

### Analysis of the association of S100A11 with tumor microenvironment (TME) and immune cell infiltration in BC

The tumor microenvironment (TME) includes two major non-tumor components, namely immune cells and stromal cells, both of which are useful in tumor diagnosis as well as prognostic assessment. To assess the TME score in the S100A11 high- and low-expression groups, we utilized the estimate package in R language software. The TME score includes the immunity score, stromal score, and estimate score. Furthermore, immune cell infiltration analysis was performed to explore the variations between S100A11 high- and low-expression groups. We used the CIBERSORT algorithm to assess the proportions of 22 immune cell subtypes and to evaluate the association between S100A11 and immune cells. *P* < 0.05 was used as the statistical significance level.

### Study of the link between S100A11 and immunological checkpoints in breast cancer

Immune checkpoints are crucial for tumor immune escape. We finalized 50 genes of immune checkpoint-associated proteins for subsequent analysis by reviewing relevant literature; see Supplementary Table S1^14^. The relationship between S100A11 and immune checkpoints in BC was further investigated using the corrplot package and ggplot2 of R software.

### Data analysis

Data analysis for this study was carried out using both R language software and SPSS 27.0 software. To assess the variations between the two groups, the Wilcoxon rank-sum test was utilized. Additionally, Cox regression analysis was utilized to assess the correction between S100A11 gene expression and the prognosis for patients with BC. Furthermore, Spearman correlation analysis was performed to investigate the association between S100A11 and other factors. The threshold for statistical significance was set at *P* < 0.05.

### Ethical approval

The omics and clinical information in this study were collected from publicly accessible TCGA databases and other public database, therefore and we did not need the ethical approval from local committee.

## Results

### S100A11 is overexpressed in BC tissues

**Figure 1:**
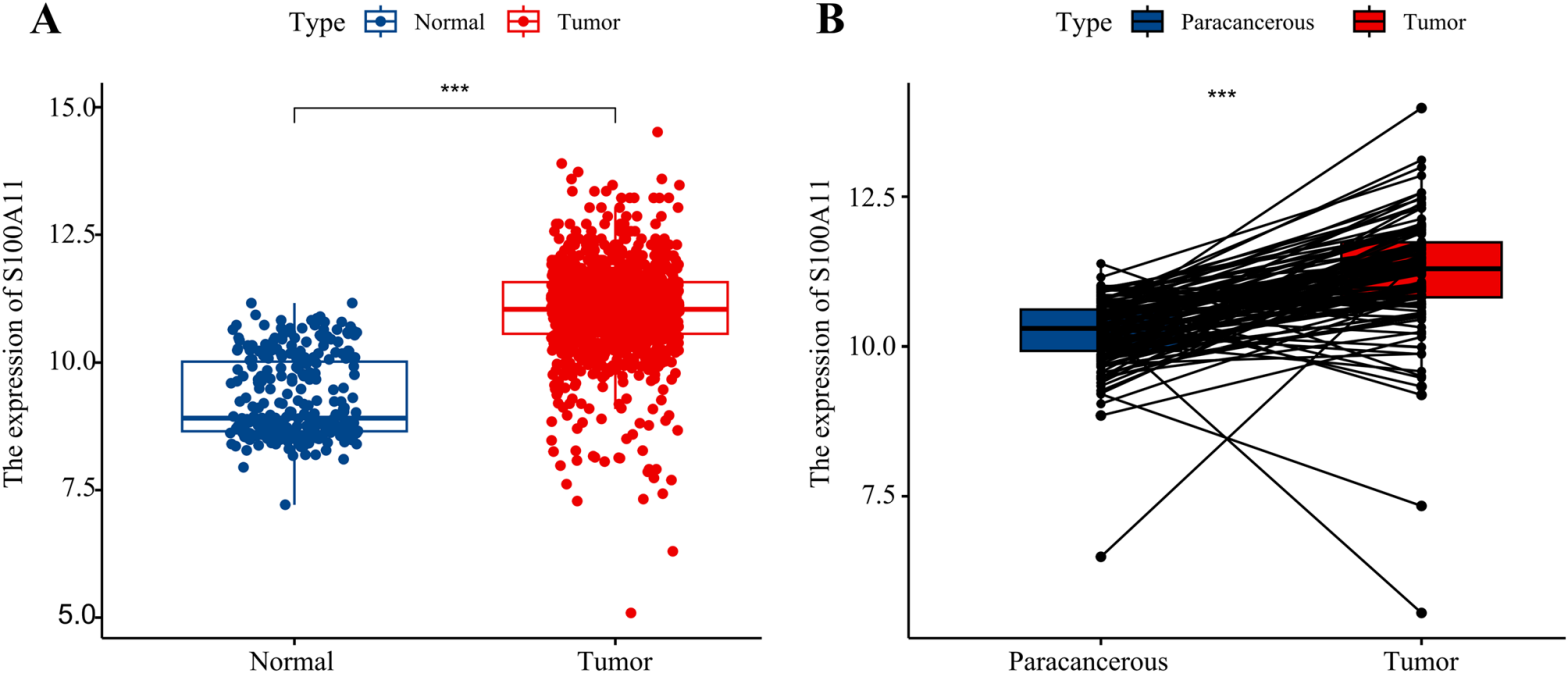
The expression levels of S100A11 mRNA in breast cancer tissues (**A**) Expression of S100A11 mRNA in BC tissues and normal breast tissues (TCGA-GTEx). The significance of the difference was tested with an unpaired student’s t test. (**B**) Expression of S100A11 mRNA in BC tissues and para-cancerous tissues (TCGA). The significance of the difference was tested with a paired student’s t test. *Note* Para-cancerous tissues usually refer to the normal breast tissue 2 cm from the edge of the tumor lesion. ***P < 0.001.

**Figure 2:**
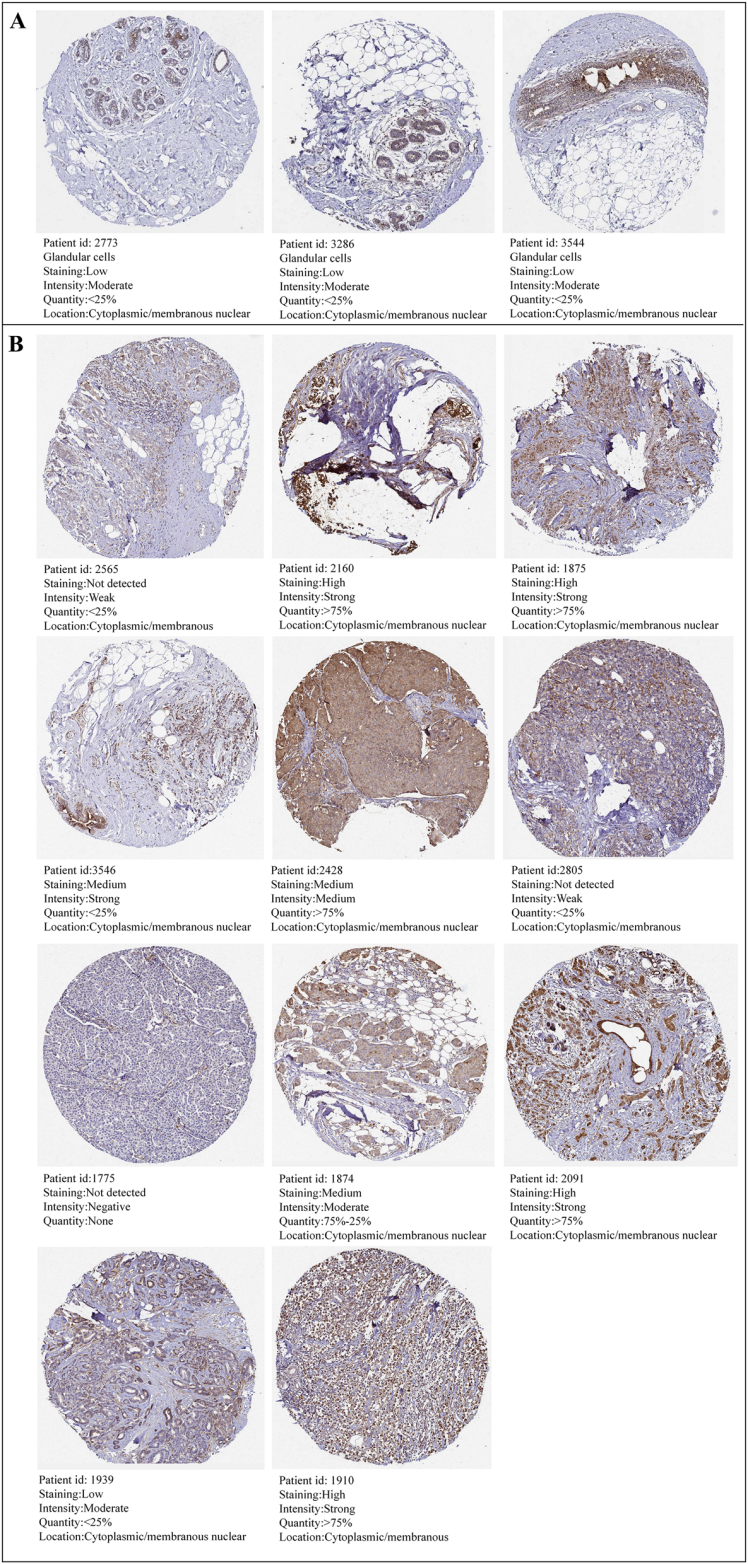
Protein expression of S100A11 in breast cancer tissues compared with breast normal tissues in the HPA database (immunohistochemistry): (**A**) breast normal tissues. (**B**) Breast cancer tissues. HPA042745 is the antibody ID of S100A11 in the HPA database.

As shown in Fig. 1A,B, S100A11 mRNA was substantially more expressed in BC tissues than in normal breast tissues in both unpaired and paired samples. Furthermore, the immunohistochemical results from the HPA database showed that S100A11 exhibited low expression in normal breast tissues, characterized by low staining, moderate intensity, and < 25% quantity (Fig. 2A). As shown in Fig. 2B, out of the 11 breast cancer tissues examined, 7 exhibited medium- to high-intensity staining and moderate- to strong-intensity. These results indicate a high expression of S100A11 in breast cancer tissues.

### Correlation between S100A11 and clinical features of patients with BC

In this study, a total of 874 BC patients were enrolled, and their clinical information is provided in Table 1. As shown in Supplementary Fig. 1A–F, there was no statistically significant association between S100A11 expression and clinical features such as age, stage, T, M, and N.

**Table 1:** Clinical characteristics of patients with BC (TCGA, n = 874).

Clinical factor	Alive(n = 745)	Dead(n = 129)	Total(n = 874)
Age			
Median[min, max]	57[26,89]	61[26,89]	57.5[26,89]
Gender (n(%))			
Female	735	128	863 (98.74)
Male	10	1	11 (1.26)
Stage (n(%))			
Stage I	144(19.3)	15 (11.6)	159 (18.2)
Stage II	446 (59.9)	60 (46.5)	506 (57.9)
Stage III	152 (20.4)	39 (30.2)	191 (21.9)
Stage IV	3 (0.4)	15 (11.6)	18 (2.1)
T (n(%))			
T1	208 (27.9)	27 (20.9)	235 (26.9)
T2	443 (59.5)	69 (53.5)	512 (58.6)
T3	77 (10.3)	20 (15.5)	97 (11.1)
T4	17 (2.3)	13 (10.1)	30 (3.4)
M (n(%))			
M0	742 (99.6)	114 (88.4)	856 (97.9)
M1	3 (0.4)	15 (11.6)	18 (2.1)
N (n(%))			
N0	383 (51.4)	41 (31.85)	424 (48.5)
N1	244 (32.8)	55(42.6)	299 (34.2
N2	77 (10.3)	19(14.7)	96 (11.0)
N3	41 (5.5)	14(10.9)	55 (6.3)

### Prognostic value of S100A11 in BC

**Figure 3:**
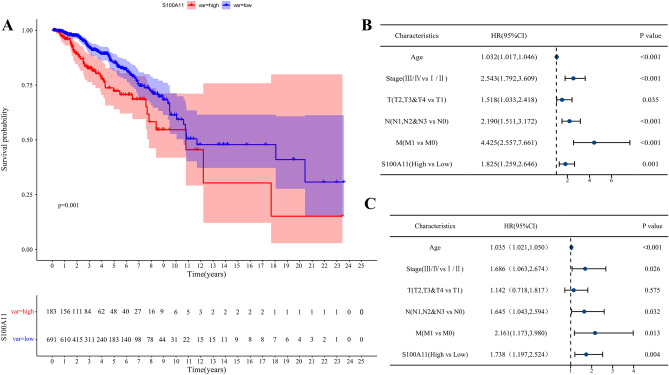
The effect of 100A11 expression on the prognosis (OS) of patients with BC. (**A**) Kaplan–Meier survival curves for OS of patients with BC. (**B**)Forest plot of the univariate Cox regression analysis; (**C**) Forest plot of multivariate Cox regression analysis.

To further understand the value of S100A11 in the prognosis for patients with BC, we obtained the best cutoff value of the S100A11 expression (11.90084). Samples were separated into the S100A11 high- and low-expression groups. Survival curves indicated that patients with BC in the S100A11 high-expression group had a shorter OS than those in the low-expression group (Fig. 3A). The results of the univariate Cox regression analysis demonstrated that these factors were related to the BC prognosis; all characteristics, except age, have significant and high HR values and are good independent indicators of a bad outcome for patients with BC, with M and Stage being the best, followed by N and the expression of S100A11(Fig. 3B).

However, multivariate analysis showed significant HR in the cases of M (HR = 2.161) and S100A11 (HR = 1.738), followed by Stage (HR = 1.686) and N (HR = 1.645) (Fig. 3C). Both analyses confirmed the importance and significance of S100A11 expression in a patient’s survival prognosis.

### Establishment and validation of a Nomogram for forecasting the prognosis in BC patients

**Figure 4:**
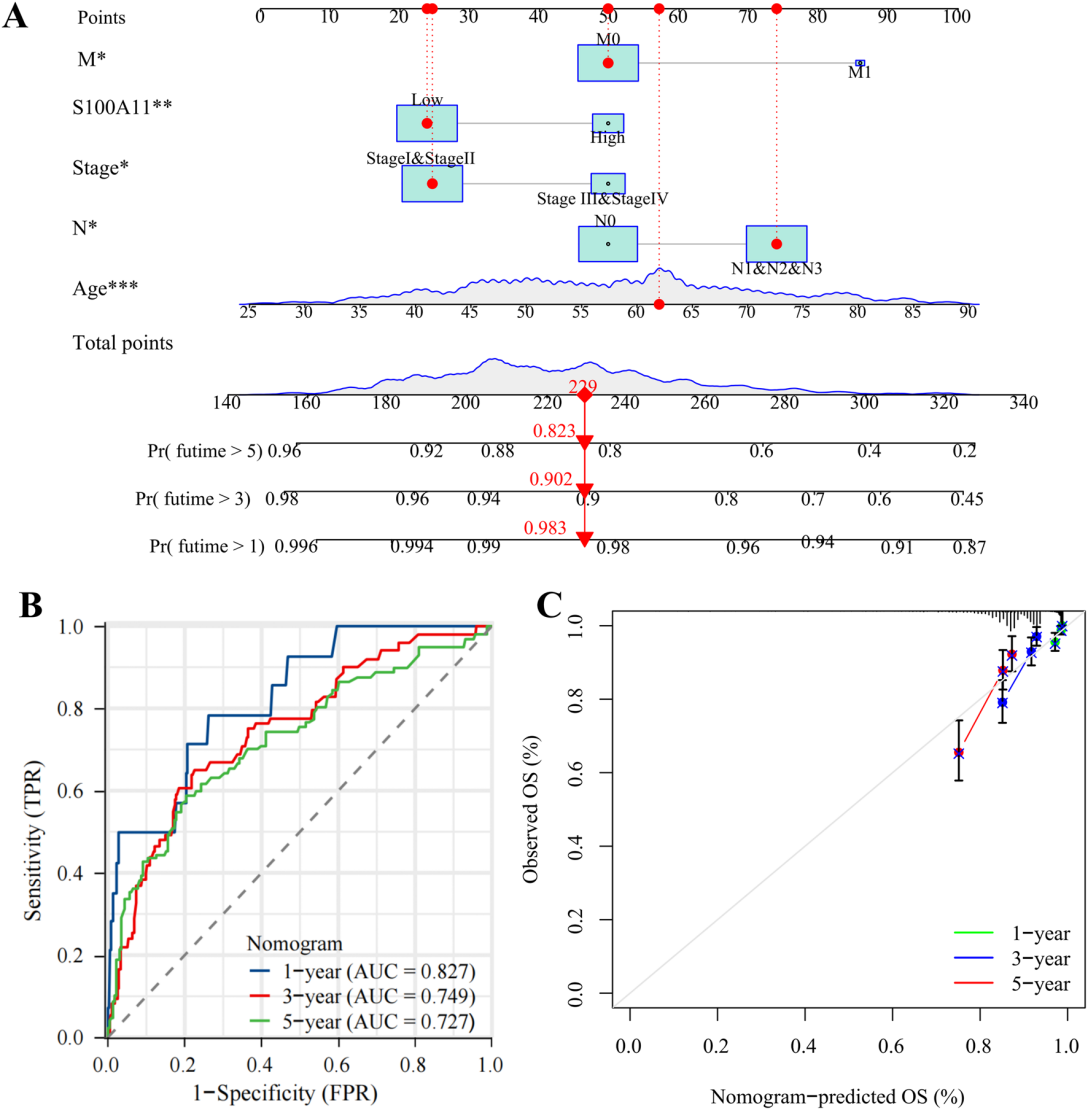
Establishing and validating a nomogram for forecasting the prognosis of patients with BC. (**A**) Nomogram for anticipation the OS of patients with BC at 1, 3, and 5 years. (**B**) ROC curves for validating the nomogram’s accuracy in predicting the prognosis of BC; (**C**) Calibration curves for validating the nomogram’s consistency in predicting the prognosis of BC. *P < 0.05,**P < 0.01;***P < 0.001.

To specifically quantify the probability of survival in BC prognosis, a nomogram was created to anticipate the OS of patients with BC at 1 year, 3 years, and 5 years (Fig. 4A). The nomogram contains five factors, including S100A11 (high and low expression), age, clinical stage, N, and M.

In this model, the regression coefficients for S100A11 (high), age, stage (stage III/IV), N (N1&N2&N3), and M1 were 0.560, 0.034, 0.544, 0.521, and 0.780, respectively.

Subsequently, the C-index of this model was 0.746 (95% CI 0.697–0.795). Meanwhile, time-dependent ROC analysis revealed that the nomogram’s AUC values for predicting BC patients’ 1-year, 3-year, and 5-year OS were respectively 0.827, 0.749, and 0.727 (Fig. 4B). Furthermore, a calibration curve indicated a good agreement between the anticipated and observed values for 1-year, 3-year, and 5-year OS (Fig. 4C).

Consequently, the C-index (0.746), time-dependent ROC curves, and calibration curves together confirmed that the nomogram had a certain degree of accuracy and consistency in predicting the prognosis of patients with BC.

Meanwhile, we constructed a nomogram by incorporating only the clinical data (age, clinical stage, N, and M) as shown in Supplementary Fig. 2. The nomogram containing S100A11 compared favorably with it in predicting 3- and 5-year survival in breast cancer.

### S100A11 mutation and its relationship to the prognosis of patients with BC

**Figure 5:**
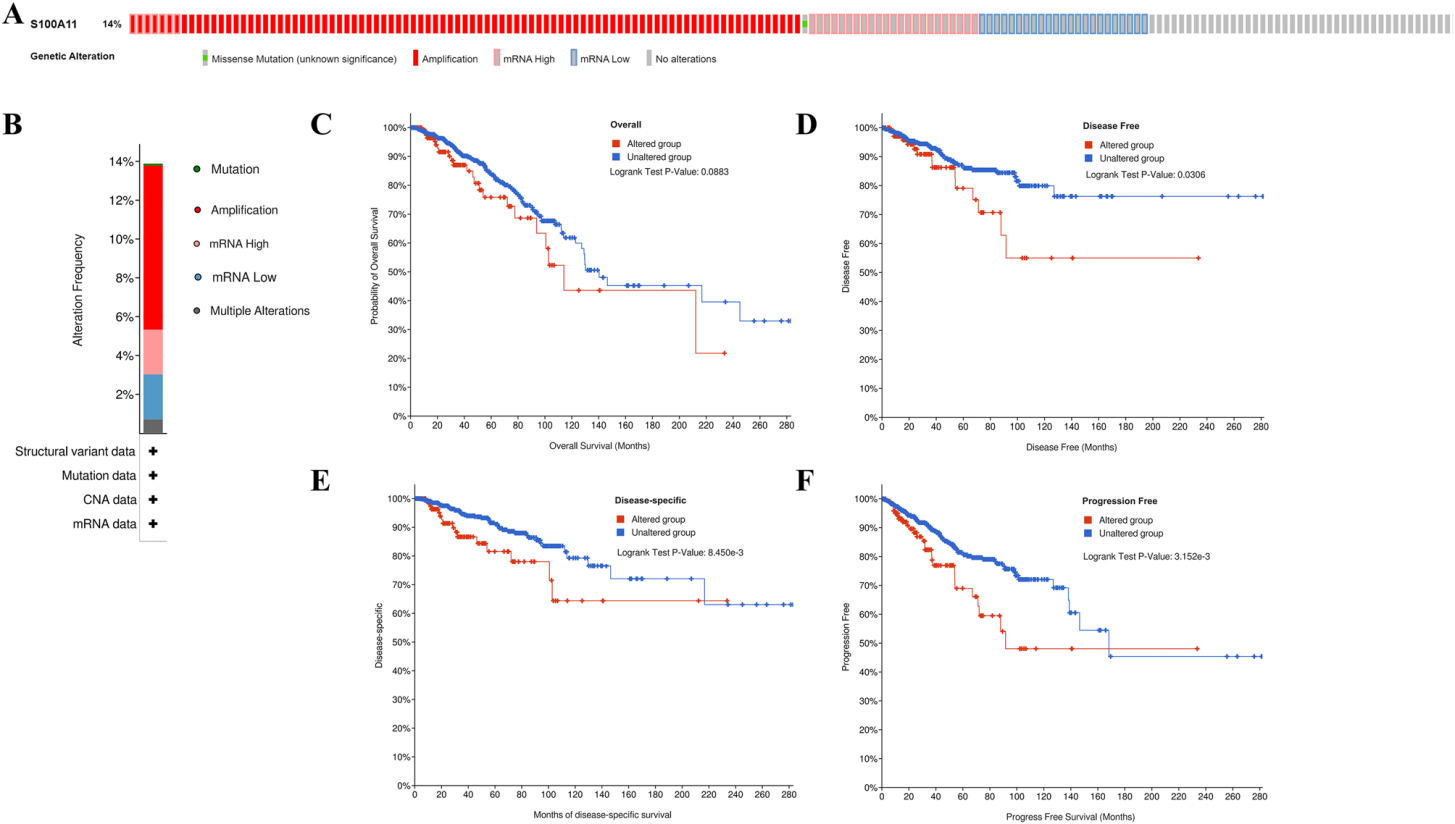
S100A11 mutation and its connection with the prognosis of BC patients. (**A**) Mutation rate of S100A11. (**B**) Total mutations of S100A11; (**C**–**F**) Kaplan–Meier survival curves for outcome of BC patients based on the alternation level of S100A11 (**C**) OS; (**D**) DFS; (**E**) DSS; (**F**) PFS.

Following that, we utilized the cBioPortal to analyze the S100A11 mutation in BC and evaluate its connection with patients’ prognosis. The mutation rate of S100A11 was 14%, and it mainly occurred in amplification, as illustrated in Fig. 5A,B. In addition, although genetic alterations of S100A11 were not found to be linked to the OS of patients with BC (Fig. 5C), the S100A11 mutation group was associated with poorer DFS, DSS, and PFS for BC patients compared to the group without mutations (Fig. 5D–F). This suggests that breast cancer patients with S100A11 mutations had a worse outcome than those without mutations.

### The PPI networks and Biological Functions Enrichment analysis of S100A11-related genes in BC

Based on RNA-sequencing data for BC from the TCGA database, the spearman correlation coefficients of all genes with S100A11 were calculated by cor.test() using R software. As a result, 1469 genes were found to be correlated with S100A11, of which 1006 genes were negatively correlated and 463 genes were positively correlated (Supplementary Table S2).

**Figure 6:**
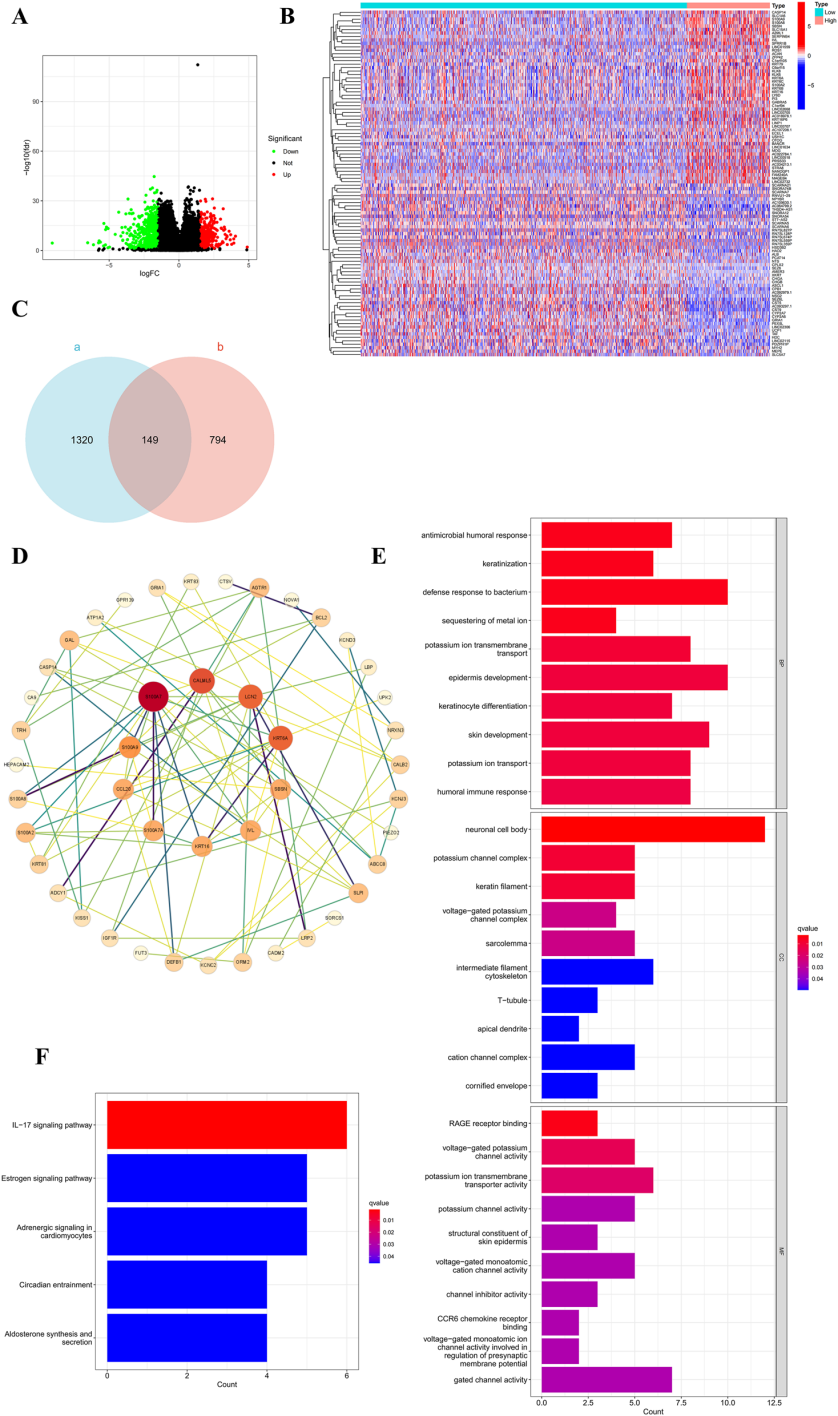
GO and KEGG enrichment analyses of S100A11-related genes. (**A**) Volcano plot of DEGs between S100A11 high- and low-expression groups (Wilcoxon rank-sum test). (**B**) Heatmap of the top 50 DEGs positively and negatively correlated. (**C**) Venn diagram (a. genes with co-expression with S100A11; b.S100A11-associated DGEs). (**D**) The PPT network of S100A11-associated 149 genes; (**E**) circle plots of GO pathway enrichment analysis; (**F**) KEGG enrichment analysis.

Then, based on the best cutoff value of S100A11 expression, samples were split into S100A11 high- and low-expression groups. Next, in R software, we utilized the limma package to perform differential expression analysis between S100A11 high- and low-expression groups. Finally, we employed the Benjamini–Hochberg multiple test method to adjust the *P*-value (denoted as BH), and the adjusted *P*-value was less than 0.05, meaning that the difference was of statistical significance. The screening threshold for DEGs was set at |log2(Fold Change)|> 1.5 as well as BH < 0.05. According to the results, a total of 943 genes were identified as relevant DGEs in the S100A11 high- and low-expressing groups. Out of these, 346 genes exhibited positive expression while 599 genes showed negative expression (Supplementary Table S3, Fig. 6A). Furthermore, a heat map displayed the top 50 DEGs with both positive and negative expressions (Fig. 6B).

Subsequently, we intersected the S100A11 co-expressed genes and S100A11-related DEGs, and found the common 149 genes (Supplementary Table S4, Fig. 6C. Based on the 149 genes, we utilized the STRING database to construct PPI networks and used Cytoscape software to remove the isolated nodes to form a protein network interaction map. Finally, according to the interaction degree shown in the PPI network, we screened out the top 4 genes associated with S100A11: S100A7 (R = 0.41, logFC = 2.23), CALML5 (R = 0.34, logFC = 1.64), LCN2 (R = 0.40, logFC = 1.84), and KRT6A (R = 0.31, logFC = 3.29) (Fig. 6D).

To better understand the possible biological functions as well as signaling pathways of S100A11-related genes, the clusterProfiler package in R software was utilized to perform the GO and KEGG pathway enrichment analyses for the identified 149 genes. The findings of the GO enrichment analysis revealed that the biological functions of S100A11-related genes are mainly involved in biological BP, CC, and MF (Supplementary Table S5 and Fig. 6E). BP aspects were involved mainly in defense response to bacteria, epidermis development, humoral immune response, and skin development. CC aspects were linked with the neuronal cell body, potassium channel complex, keratin filament, voltage − gated potassium channel complex, and sarcolemma. MF aspects were mainly involved in molecular functions such as RAGE receptor binding, voltage–gated potassium channel activity, potassium ion transmembrane transporter activity, and potassium channel activity.

KEGG enrichment analysis indicated that S100A11-related 149 genes were primarily enriched in the IL-17 signaling pathway(*P* < 0.05) (Supplementary Table S5, Fig. 6F).

### Association of S100A11 expression with immunity score, stromal score, and immune cell infiltration in BC

**Figure 7:**
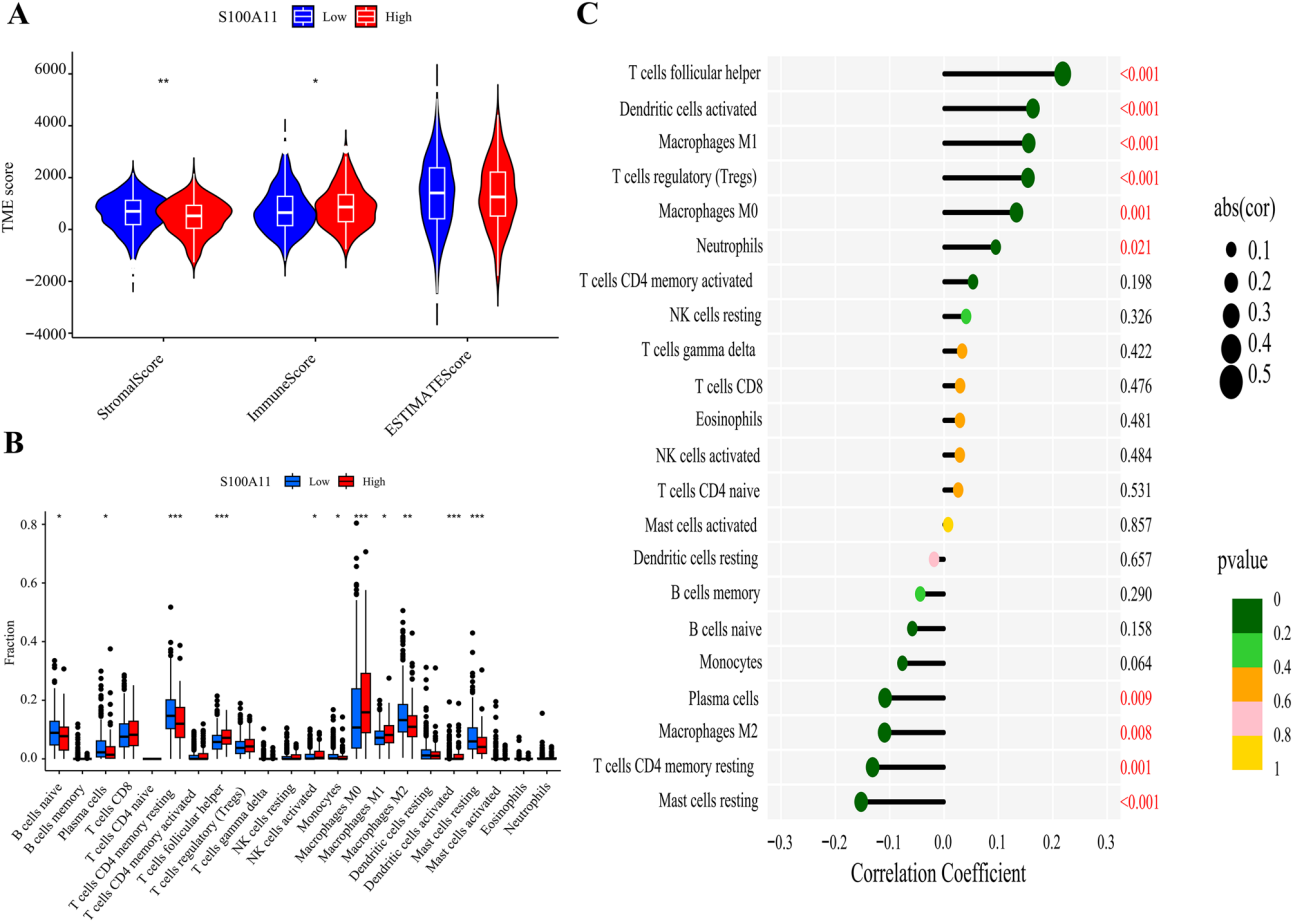
Correlation Analysis between S100A11 and TME in BC. (**A**) Comparison of tumor microenvironment score between S100A11 high- and low-expression groups. (**B**) Fractions of 22 immune cells between S100A11 high- and low-expression groups in BC. (**C**) Spearman correlation analysis between S100A11 and 22 immune cells in BC. *Note* *P < 0.05; ** *P* < 0.01; ***P < 0.001.

The samples were classified into S100A11 high- and low-expression groups based on the best cut-off value (11.90084) of S100A11 expression. In R software, we utilized the estimate and limma packages to evaluate TME scores. The findings revealed that in the S100A11 high-expression group, the immunological score was greater than in the low-expression group, while the stromal score was lower (Fig. 7A, *P* < 0.05). Furthermore, based on the CIBERSORT algorithm, we also compared the difference in immune cell infiltration between S100A11 high- and low-expression groups. In the S100A11 high-expression group, there were higher fractions of T cells follicular helper, NK cells activated, macrophages M0, macrophages M1, and dendritic cells activated compare to the low-expression group. Conversely, the S100A11 low-expression group exhibited lower fractions of B cells naive, monocytes, macrophages M2, T cells CD4 memory resting, plasma cells, and mast cells resting (Fig. 7B, *P* < 0.05).

Meanwhile, Spearman correlation analysis demonstrated that S100A11 was positively related to T cells follicular helper, dendritic cells activated, macrophages M1, T cells regulatory (Tregs), macrophages M0, and neutrophils. Conversely, it showed negative relationships to T cells CD4 memory resting, plasma cells, macrophages M2, and mast cells resting (Fig. 7C, *P* < 0.05).

### Study of the relationship between S100A11 expression and immune checkpoints in BC

**Figure 8:**
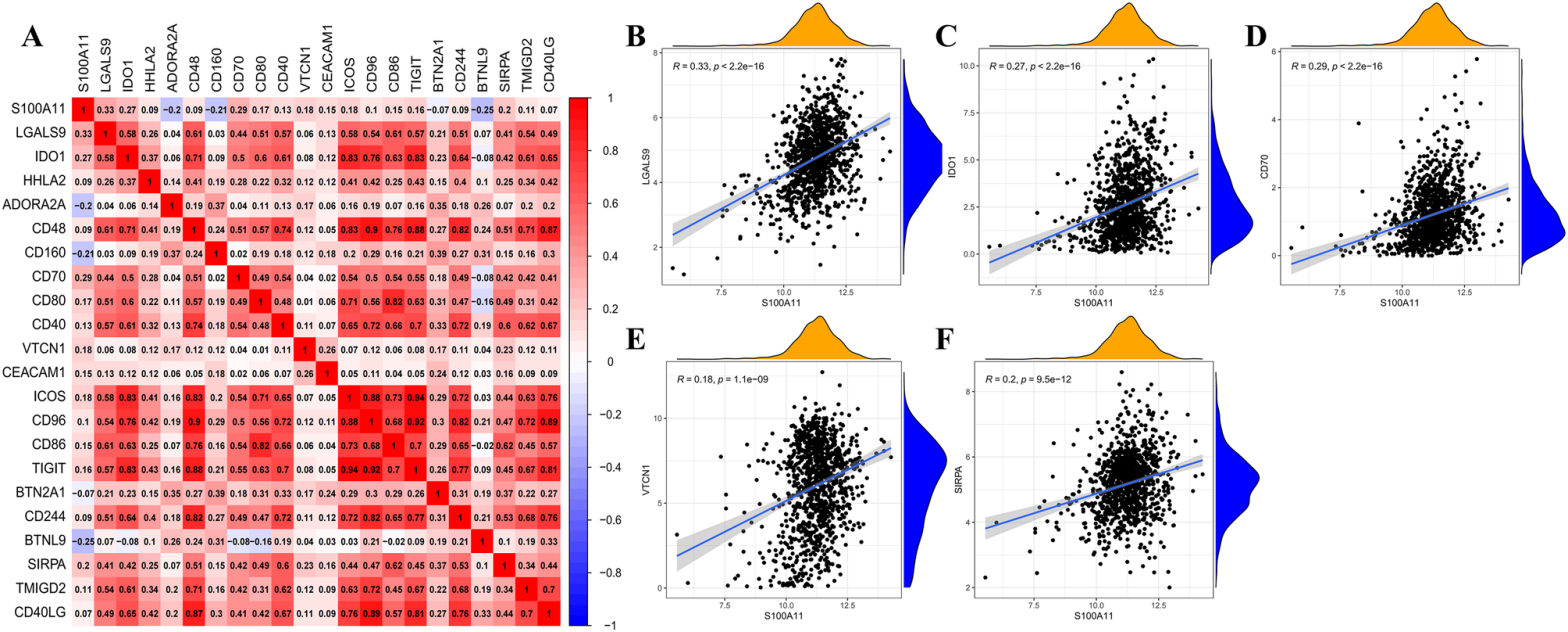
Correlation analysis between S100A11 and immune checkpoint-related genes (Spearman correlation analysis). (**A**) Co-expression heatmap; (**B**–**F**) Scatter plots of the top 5 immune checkpoint-related genes associated with the presence of S100A11 (**B**) LGALS9; (**C**) IDO1; (**D**) CD70; (**E**) VTCN1; (**F**) SIRPA.

To further investigate the potential association between S100A11 expression in BC and immunological checkpoints, we conducted spearman correlation analysis. The results revealed a significant positive correlation between S100A11 and 17 immune checkpoint-related genes (Fig. 8A, *P* < 0.05). Additionally, scatter plots were used to illustrated the association between S100A11 and the top 5 immune checkpoint-associated genes (LGALS9(R = 0.33), IDO1(R = 0.27), CD70(R = 0.28), VTCN1(R = 0.18), and SIRPA(R = 0.20)) (Fig. 8B–F).

## Discussion

At present, breast cancer has become the number one tumor in the world, characterized by its high incidence rate. Despite notable achievements in breast cancer treatment, a considerable number of patients with BC still experience mortality due to recurrence and metastasis, which still seriously jeopardize women's lives and health.

Several studies have shown that S100A11 is aberrantly expressed at both the mRNA and protein levels in a variety of malignant tumors. This aberrant expression is associated with its role as an oncogenic factor in the majority of cancer types. Furthermore, it is essential for cancer cell proliferation, angiogenesis, metastasis, immune escape, and drug resistance^5,15,16^. For example, Cui et al.^17^ demonstrated that S100A11 overexpression was linked to a poorer prognosis for patients with gastric cancer (GC). They further found that S100A11 could accelerate tumor progression by regulating MMP activity as well as the EMT process. Additionally, S100A11 knockdown could markedly inhibit the ability of GC cells to migrate and invade. Moreover, the resistance of cancer cells to chemotherapeutic agents such as fluorouracil (5-FU) and cisplatin was reduced upon S100A11 knockdown. In addition, Zeng et al.^7^ indicated that S100A11 can affect the pentose phosphate pathway by regulating TKT, thereby promoting cell proliferation and migration in pancreatic ductal carcinoma. In our study, we evaluated S100A11 expression in BC at both the mRNA and protein levels. Our findings were consistent with an abnormally high expression in most tumor tissues^16^. Additionally, we evaluated the connections between S100A11 expression and different clinicopathologic features. However, we did not observe any statistically significant association between S100A11 expression and clinical features, including age, clinical stage, T, N, and M, of breast cancer patients.

S100A11 has been found to be linked to the prognosis of a number of cancers in various studies. For example, Wang et al.^18^ showed that in gliomas, there was a positive correlation between S100A11 expression and pathologic grade, while it was negatively correlated with patient survival time, portending a poor prognosis. Additionally, Zhang et al.^19^ conducted a study using multiple databases, and their results suggested that higher S100A11 expression was significantly associated with a worse prognosis in patients with low-grade gliomas. In this study, samples were separated into S100A11 high- and low-expression groups based on the best cutoff value (11.90084) of S100A11 expression. One-factor and multifactor Cox regression analyses revealed that S100A11 overexpression was one of the independent risk indicators for a worse prognosis in individuals with BC. Several studies have also shown a strong association between S100A11 and an unfavorable prognosis in patients with BC^10,20^, which is consistent with the findings of our study. Based on the results of the multifactor Cox regression analysis, we believe that the expression of S100A11 is of significant importance in predicting the prognosis of breast cancer patients. Therefore, we developed a novel predictive model that combines clinical parameters (age, clinical stage, N, and M) with S100A11 expression to predict the prognosis of breast cancer patients. The model showed a certain level of consistency and accuracy. However, external validation of the model is still needed. However, the mechanisms underlying the impact of S100A11 on breast cancer development and prognosis remain unclear, and further research is needed to delve into the possible mechanisms.

Genomic instability is indeed a significant contributor to tumorigenesis, malignant progression, distant metastasis, and treatment resistance^21^. Moreover, some studies have suggested that tumors can be facilitated by mutations in genes that promote the selective growth of tumor cells^22^. In our study, we applied the cBioPortal to look for the alteration of the S100A11 gene in TCGA-BRCA, where we observed a mutation rate of 14%. Meanwhile, we found that S100A11 mutations mainly occurred in amplifications. It has been shown that the amplification of chromosome copy number of 1q21.3 has been proven to be a biomarker and therapeutic target for BC recurrence^23^. The chromosome of S100A11 is localized in the 1q21 region band, which is poorly stable and prone to changes such as chromosomal translocation. These alterations are closely related to tumorigenesis^5^. At the same time, we also found a strong association between mutations in S100A11 and DFS, DSS, and PFS in breast cancer patients. It indicates that the alteration of S100A11 could be implicated in a worse prognosis for BC patients. However, more research on the particular mechanism has to be done in the future.

In this study, we used these 149 genes associated with S100A11 to construct a PPI network. Based on the degree of interaction observed within this network, we identified the top 4 genes that are associated with S100A11: S100A7(R = 0.41, logFC = 2.23), CALML5(R = 0.34, logFC = 1.64), LCN2(R = 0.40, logFC = 1.84), and KRT6A(R = 0.31, logFC = 3.29). Several studies have shown that S100A7^24^, and LCN2^25,26^ are associated with poor prognosis in BC and they have also been implicated in cancer invasion and metastasis. Kurozumi et al. reported that CALML5, a calcium-regulated protein-like protein, is linked to lymphatic vascular invasion in early-stage breast cancer and may have potential prognostic significance^27^. Additionally, Park et al. found that high KRT6A expression is strongly correlated with a poor prognosis in patients with recurrent breast cancer^28^. Whether S100A11 works together with these genes to affect the prognosis of BC and its specific mechanism of action need to be verified by further experiments.

The IL-17 signaling pathway is an essential inflammatory signaling system that activates downstream signaling pathways by interacting with IL-17 family cytokines and their receptors, and it is involved in inflammation, autoimmune disorders, transplant rejection, and malignancies^29–32^ For instance, Bin Wang et al. showed that IL-17 could induces proliferation and migration through the activation of PI3K/Akt1/NF-κB-p65^33^.Additionally, Zhenhua Wu et al. reported that IL-17A/IL-17RA promotes invasion and activates MMP-2 and MMP-9 expression via the p38 MAPK signaling pathway in non-small cell lung cancer^34^. In this study, we performed enrichment analysis on S100A11-associated co-expressed genes and S100A11-associated DEGs. The findings from the KEGG enrichment analysis revealed that the IL-17 signaling pathway was the major signaling pathway involved in S100A11. A study demonstrated that IL-17A could enhance cancer cell proliferation, metastasis, and micro-angiogenesis in BC by multiple pathways^35^. In addition, it has been shown that elevated IL-17B is associated with a poor prognosis in patients with BC^36^. However, the involvement of the IL-17 signaling pathway in BC development, as well as the specific mechanism of action, need to be verified by further experiments.

In recent years, many researchers have grown increasingly interested in the influence of the TME on tumorigenesis and progression, with a particular emphasis on the influence of immune cells in the TME^37,38^. Macrophages are one of the most common immune cells in the TME and have been linked to tumor development. For example, Lu et al.^39^ showed that S100A7 was a potential diagnostic and prognostic marker for patients with esophageal squamous carcinoma. They also demonstrated that S100A7 promotes macrophage infiltration and polarization, as well as tumor angiogenesis. Based on the TIMER database, Zheng et al.^40^ discovered a favorable association between S100A11 and several immune cells, including B cells and macrophages. In this work, we examined the association between S100A11 and immune cell infiltration and discovered that immune infiltration was greater in the S100A11 high-expression group compared to the low-expression group. Meanwhile, S100A11 is correlated with many types of immune cells, including M0/M1 macrophages and Treg cells. However, it is unclear whether S100A11 could participate in the development of BC by influencing immune cells such as macrophages, and more research in ex vivo as well as in vivo studies is still required in the future.

Immunotherapy is a novel therapeutic strategy in the field of oncology, and the expression of immune checkpoints is one of the key determinants of immunotherapy. Immune checkpoints refer to a variety of immune inhibitory mechanisms that maintain self-tolerance and control the duration and amplitude of immune responses in a physiological state. Tumors have the capability to select certain immune checkpoint pathways, such as CTLA4 and PD1/PDL-1, to achieve immune resistance, thereby promoting their escape from the immune system^41^. In our work, we assessed the association between S100A11 and 50 common immune checkpoint-associated molecules. The findings indicated a positive correlation between S100A11 and 17 immune checkpoint-associated genes, including LGALS9, IDO1, CD70, VTCN1, and SIRPA. Therefore, in conjunction with the immune infiltration, we speculate that S100A11 may be associated with tumor immune escape, potentially affecting the efficacy of immunotherapy and, in turn, the prognosis of breast cancer. However, the specific mechanisms need to be studied in depth.

## Conclusion

In summary, S100A11 could serve as a biomarker for poor prognosis in breast cancer patients. It is associated with immune infiltration and may influence breast cancer progression through the IL-17 signaling pathway. More in vitro and in vivo experiments will be needed to validate our findings in the future and unravel the potential molecular mechanisms in breast cancer.

### Supplementary Information

Supplementary Figures.

Supplementary Table S1.

Supplementary Table S2.

Supplementary Table S3.

Supplementary Table S4.

Supplementary Table S5.

## Data Availability

The datasets analyzed for this sttudy can be obtained from the TCGA-BRCA project((https://portal.gdc.com), the Genotype-Tissue Expression(GTEX)dataset from the UCSC Xena database (https://xena.ucsc.edu/), HPA dadabase (https://www.proteinatlas.org), cBioPortal ((https://www.cbioportal.org), STRING (https://string-db.org/).
