# Supplementary material for: Exploring the prognostic value of S100A11 and its association with immune infiltration in breast cancer
Source: Sci Rep. 2023 Dec 21;13:22922. doi: 10.1038/s41598-023-50160-x (PMC10739898; doi:10.1038/s41598-023-50160-x)
Supplement: Supplementary file 1 — Supplementary Figures. [file 41598_2023_50160_MOESM1_ESM.docx]

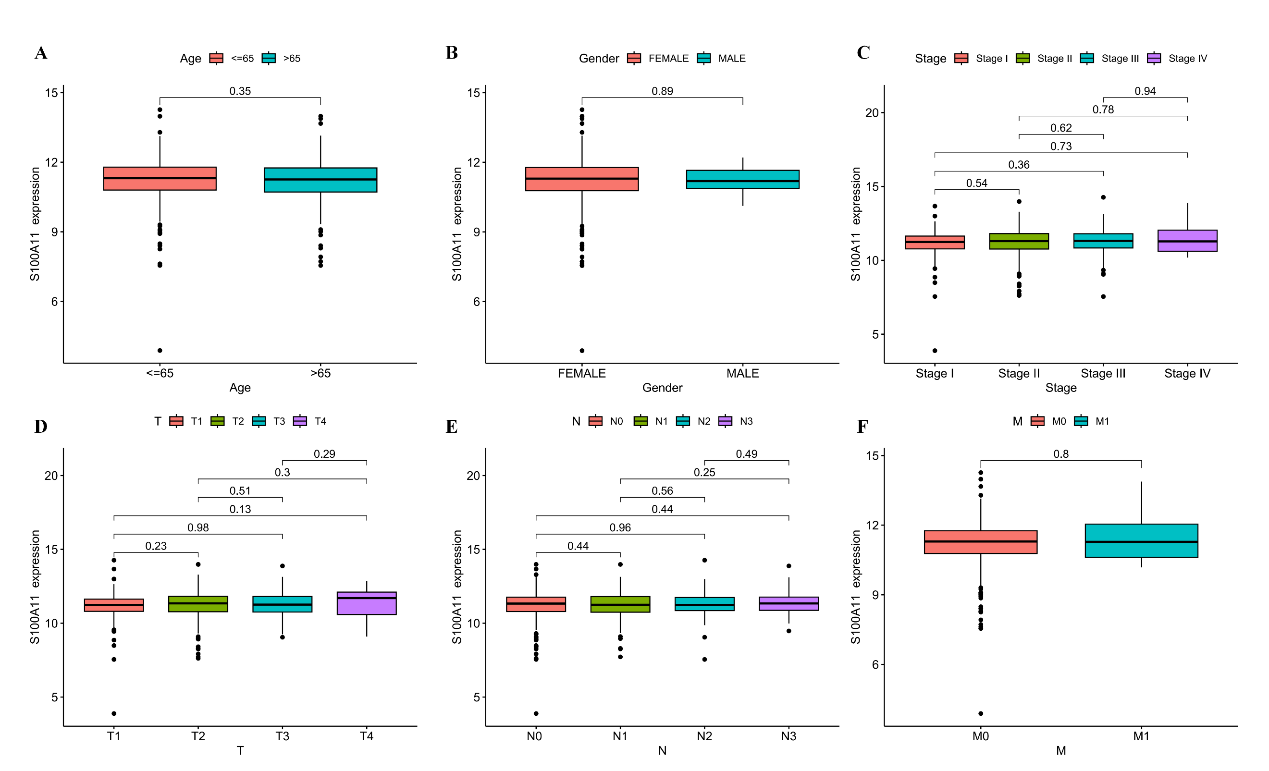
 Supplementary Fig.S1 Analysis of various clinical characteristics associated with S100A11 expression. A-F S100A11 was not significantly different in age, gender, Stage, Pathologic-, T-, N-,M-stage in TCGA database. The significance of the difference was tested by one‐way ANOVA. A. age; B. gender; C. stage ; D. T stage; E. N stage; F:M stage.


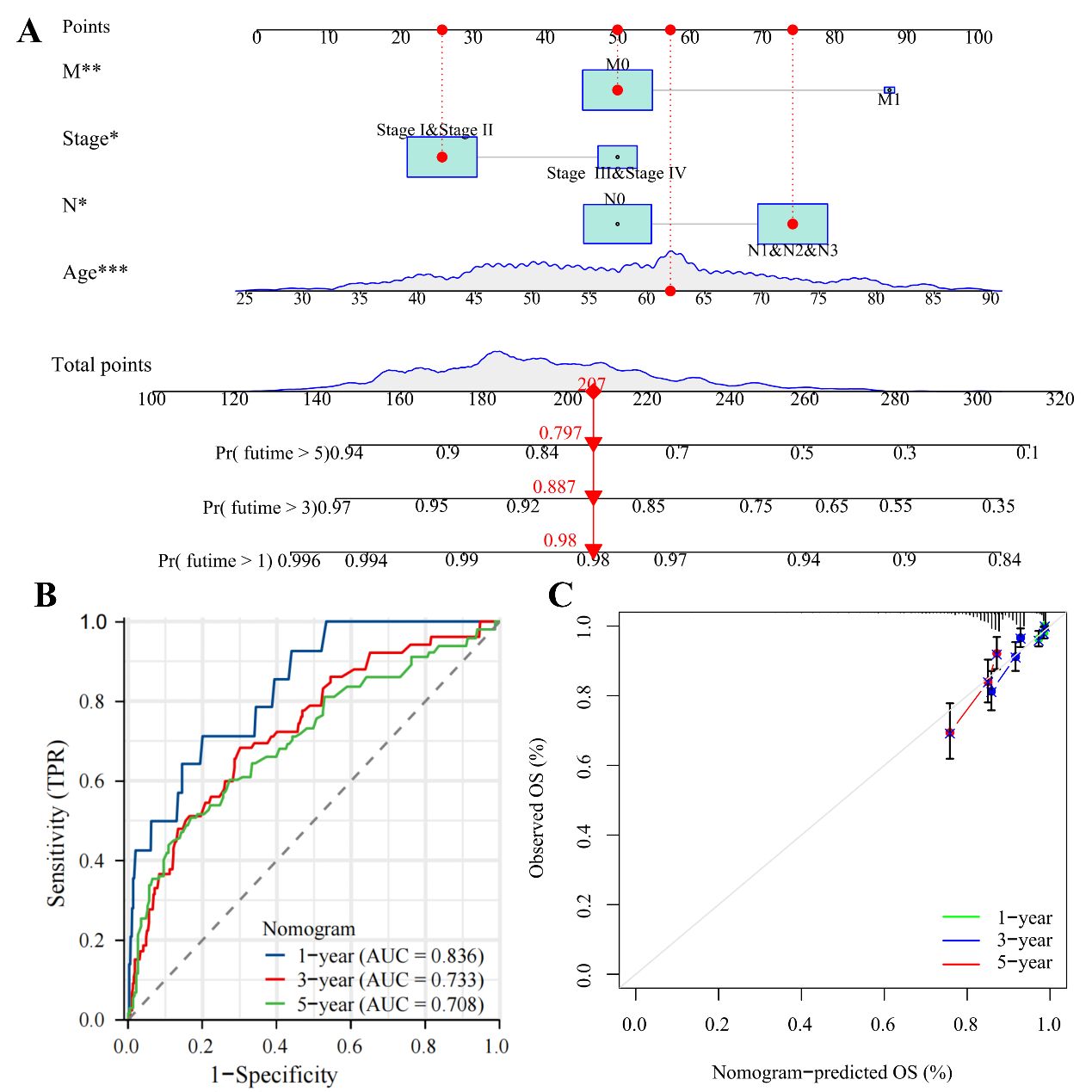


Supplementary Fig.S2 Establishing and validating a nomogram for forecasting the prognosis of patients with BC. A The construction of a nomogram anticipating the OS of patients with BC at 1, 3, and 5 years(note:**P*<0.05,***P*<0.01;****P*<0.001); B. The ROC curves for the nomogram’s prediction of the OS of patients with BC at 1, 3, and 5 years; C. The calibration curves for nomogram’s prediction of the OS of patients with BC at 1, 3, and 5 years.
