# Supplementary material for: Exploring the prognostic value of S100A11 and its association with immune infiltration in breast cancer
Source: Sci Rep. 2023 Dec 21;13:22922. doi: 10.1038/s41598-023-50160-x (PMC10739898; doi:10.1038/s41598-023-50160-x)
Supplement: Supplementary file 2 — Supplementary Table S1. [file 41598_2023_50160_MOESM2_ESM.docx]

**Supplementary Table S1 The gene list of immune check-point**

| **Serial number** | **Gene symbol** | **Serial number** | **Serial number** |
| --- | --- | --- | --- |
| 1 | ADORA2A | 26 | CD86 |
| 2 | BTNL3 | 27 | CD96 |
| 3 | 2B4（CD244） | 28 | CEACAM1 |
| 4 | B7-H3 | 29 | CTLA-4 |
| 5 | VTCN1 | 30 | DC-SIGN |
| 6 | BTLA | 31 | DNAM1 |
| 7 | BTN2A1 | 32 | GITRL |
| 8 | BTN2A2 | 33 | HHLA2 |
| 9 | BTN3A1 | 34 | HVEM |
| 10 | BTNL9 | 35 | ICOS |
| 11 | CD137 | 36 | ICOSLG |
| 12 | CD137L | 37 | IDO1 |
| 13 | CD155 | 38 | LAG-3 |
| 14 | CD160 | 39 | LGALS9 |
| 15 | CD200 | 40 | LIGHT |
| 16 | CD200R | 41 | OX40 |
| 17 | CD244 | 42 | PD1 |
| 18 | CD27 | 43 | PDL1 |
| 19 | CD28 | 44 | PDL2 |
| 20 | CD40 | 45 | SIRPA |
| 21 | CD40LG | 46 | TDO |
| 22 | CD47 | 47 | TIGIT |
| 23 | CD48 | 48 | TIM-3 |
| 24 | CD70 | 49 | TMIGD2 |
| 25 | CD80 | 50 | VISTA |
