# Supplementary material for: Exploring the prognostic value of S100A11 and its association with immune infiltration in breast cancer
Source: Sci Rep. 2023 Dec 21;13:22922. doi: 10.1038/s41598-023-50160-x (PMC10739898; doi:10.1038/s41598-023-50160-x)
Supplement: Supplementary file 3 — Supplementary Table S2. [file 41598_2023_50160_MOESM3_ESM.docx]

**Supplementary Table S2 The gene list of co expression genes with S100A11**

| **Gene** | **cor** | **pvalue** |
| --- | --- | --- |
| NECAB3 | -0.3096225 | 2.89E-26 |
| GLRB | -0.354389594 | 2.01E-34 |
| CCDC18-AS1 | -0.371596412 | 6.24E-38 |
| ZNF346-IT1 | -0.302554653 | 4.25E-25 |
| FAM185BP | -0.31499587 | 3.57E-27 |
| CDK17 | -0.448452999 | 2.05E-56 |
| ITPR1 | -0.467765841 | 7.35E-62 |
| GATA3-AS1 | -0.310647817 | 1.95E-26 |
| AC007191.1 | -0.304242973 | 2.25E-25 |
| LGALS9 | 0.345608188 | 1.02E-32 |
| C11orf24 | 0.351524731 | 0 |
| MTX1 | 0.303918456 | 2.54E-25 |
| RN7SL674P | -0.311146128 | 1.61E-26 |
| FAM47E | -0.338101578 | 2.68E-31 |
| ULK2 | -0.337400658 | 3.62E-31 |
| EXO1 | 0.311460593 | 1.42E-26 |
| POTEKP | -0.312005662 | 1.15E-26 |
| GTF2E2 | 0.355225456 | 1.37E-34 |
| DUSP28 | -0.312154173 | 1.09E-26 |
| EPM2AIP1 | -0.351935543 | 6.10E-34 |
| CHI3L2 | 0.319094007 | 7.03E-28 |
| AC026691.1 | -0.313490803 | 6.45E-27 |
| TCF3 | 0.300062848 | 1.08E-24 |
| AC008555.1 | -0.324151413 | 9.15E-29 |
| DUSP8P5 | -0.330983123 | 5.46E-30 |
| ADCY6 | -0.319891415 | 5.11E-28 |
| AL590617.2 | 0.308992344 | 3.69E-26 |
| LINC02175 | -0.305142346 | 1.60E-25 |
| ZNF594 | -0.317903626 | 1.13E-27 |
| TTC36 | -0.305699266 | 1.30E-25 |
| DESI2 | 0.320475654 | 0 |
| LINC00339 | -0.333103713 | 2.24E-30 |
| PEDS1 | 0.389343325 | 8.87E-42 |
| RUNDC1 | -0.329678751 | 9.41E-30 |
| RBL2 | -0.352691809 | 4.33E-34 |
| EIF5A | 0.31533659 | 0 |
| TUBA5P | 0.309152022 | 3.47E-26 |
| AC008268.1 | -0.369747596 | 1.52E-37 |
| AC012557.1 | -0.362103057 | 5.71E-36 |
| IVD | -0.34307518 | 0 |
| SPTBN4 | -0.421450506 | 2.30E-49 |
| DCDC1 | -0.346103539 | 8.24E-33 |
| MSTO1 | 0.338710493 | 2.07E-31 |
| FBXW8 | -0.303667098 | 2.80E-25 |
| KAT6B | -0.417380343 | 2.34E-48 |
| FAM227B | -0.348491106 | 2.86E-33 |
| TSGA10 | -0.307915574 | 5.57E-26 |
| DIS3L | -0.337399565 | 3.62E-31 |
| PER2 | -0.319565563 | 5.83E-28 |
| CNIH4 | 0.432132584 | 0 |
| GREB1 | -0.345378837 | 1.13E-32 |
| KIAA1109 | -0.431450127 | 6.69E-52 |
| DYNC2H1 | -0.341589713 | 5.95E-32 |
| ZNF345 | -0.329212247 | 1.14E-29 |
| TMPRSS6 | -0.306447272 | 9.76E-26 |
| AC020910.6 | -0.351622847 | 7.02E-34 |
| FRYL | -0.357322232 | 5.24E-35 |
| PLEKHO1 | 0.307299239 | 7.06E-26 |
| NRDE2 | -0.317946786 | 1.11E-27 |
| MTFR2 | 0.37298042 | 3.19E-38 |
| CWH43 | 0.319429232 | 6.15E-28 |
| NFIL3 | 0.346211934 | 7.85E-33 |
| AL845472.1 | -0.323040533 | 1.44E-28 |
| IRS1 | -0.323266005 | 1.31E-28 |
| ACACB | -0.344199083 | 1.90E-32 |
| C2CD4D | 0.303499126 | 2.98E-25 |
| ICE2 | -0.333622458 | 1.80E-30 |
| KIAA1328 | -0.35023766 | 1.31E-33 |
| AC005070.3 | -0.362234528 | 5.37E-36 |
| PLAU | 0.369013771 | 0 |
| LGALS3 | 0.370918449 | 0 |
| IQCH-AS1 | -0.32627349 | 3.84E-29 |
| BCL2L12 | 0.33501846 | 9.99E-31 |
| LINC02224 | -0.304010711 | 2.46E-25 |
| GTF2IP4 | -0.347942804 | 3.65E-33 |
| THAP6 | -0.383453272 | 1.78E-40 |
| PDIK1L | -0.305973648 | 1.17E-25 |
| AL356311.1 | -0.387470386 | 2.32E-41 |
| AC020917.4 | -0.331077911 | 5.25E-30 |
| RHCG | 0.316285988 | 2.15E-27 |
| EFCAB6 | -0.392824213 | 1.46E-42 |
| SGMS1-AS1 | -0.324908101 | 6.72E-29 |
| PBDC1 | 0.31692316 | 0 |
| GSTP1 | 0.368603985 | 0 |
| AC096733.2 | -0.450650848 | 5.13E-57 |
| ARRB1 | -0.410734344 | 9.70E-47 |
| AC010280.2 | 0.307176037 | 7.40E-26 |
| ZCCHC4 | -0.319521291 | 5.93E-28 |
| MIDEAS | -0.398548455 | 7.20E-44 |
| ZNF136 | -0.345738669 | 9.68E-33 |
| RETREG3 | -0.321729072 | 0 |
| MON2 | -0.304528887 | 2.02E-25 |
| YBX1 | 0.302557885 | 0 |
| SLC43A3 | 0.37896467 | 1.69E-39 |
| CCL20 | 0.392146578 | 2.08E-42 |
| PIKFYVE | -0.307688853 | 6.08E-26 |
| BID | 0.304860989 | 1.78E-25 |
| TNFRSF21 | 0.332995217 | 2.35E-30 |
| MECP2 | -0.314973087 | 3.60E-27 |
| AC007619.1 | -0.327713036 | 2.12E-29 |
| PCSK6 | -0.357413213 | 5.03E-35 |
| KRR1 | -0.36817185 | 3.24E-37 |
| TLE1 | 0.31716825 | 0 |
| C17orf113 | -0.348508329 | 2.83E-33 |
| EHD1 | 0.320746966 | 3.63E-28 |
| SIN3A | -0.304165719 | 2.32E-25 |
| UBIAD1 | -0.306419844 | 9.87E-26 |
| NUTF2 | 0.438900805 | 7.57E-54 |
| MTMR10 | -0.433995295 | 1.47E-52 |
| AC087071.2 | -0.310080015 | 2.43E-26 |
| PSENEN | 0.303522985 | 0 |
| ZNF546 | -0.306103454 | 1.11E-25 |
| TTC8 | -0.33771761 | 3.16E-31 |
| AC020661.1 | 0.308530508 | 4.40E-26 |
| B3GNT7 | 0.326636272 | 3.31E-29 |
| CRABP1 | 0.33244998 | 2.95E-30 |
| MINDY2 | -0.303750125 | 2.71E-25 |
| CD302 | -0.326329416 | 3.75E-29 |
| ACTR8 | -0.317664139 | 1.24E-27 |
| CHST6 | 0.307939599 | 5.52E-26 |
| AL122035.1 | -0.31158817 | 1.35E-26 |
| TNFRSF14-AS1 | -0.346720871 | 6.27E-33 |
| ARSI | 0.32944932 | 1.03E-29 |
| EFNA3 | 0.414426953 | 1.24E-47 |
| SLC35E3 | -0.347000132 | 5.54E-33 |
| SUDS3P1 | -0.305907682 | 1.20E-25 |
| AC007406.4 | -0.319032574 | 7.21E-28 |
| WWP1 | -0.372794558 | 0 |
| ULBP1 | 0.307843537 | 5.73E-26 |
| SPG11 | -0.355275599 | 0 |
| ADCY1 | -0.336129759 | 6.23E-31 |
| LAMA3 | -0.369360527 | 1.83E-37 |
| CHDH | -0.342202983 | 4.56E-32 |
| POMP | 0.302775057 | 0 |
| LINC01087 | -0.32076681 | 3.60E-28 |
| CCDC58 | 0.312901514 | 8.11E-27 |
| IRS2 | -0.328508831 | 1.53E-29 |
| AL353807.5 | 0.430723947 | 1.03E-51 |
| PDSS1 | 0.364367691 | 1.97E-36 |
| PHF3 | -0.302528736 | 4.29E-25 |
| RN7SL262P | -0.306734275 | 8.75E-26 |
| AC002064.2 | -0.327988671 | 1.89E-29 |
| GGCT | 0.393892768 | 8.38E-43 |
| CCNE1 | 0.378306441 | 2.34E-39 |
| ZNF407 | -0.389939584 | 6.53E-42 |
| NXPH3 | -0.380401646 | 8.24E-40 |
| ACTB | 0.574841795 | 0 |
| NXN | 0.397980804 | 9.74E-44 |
| SHPRH | -0.317162968 | 1.52E-27 |
| DZIP3 | -0.316683696 | 1.83E-27 |
| SH3GL1 | 0.323205872 | 0 |
| CACYBP | 0.321153526 | 0 |
| CAPS2 | -0.350800791 | 1.02E-33 |
| AKR1B15 | 0.344067023 | 2.02E-32 |
| PGGT1B | -0.322191705 | 2.03E-28 |
| MAG | -0.353971551 | 2.43E-34 |
| KCNC2 | -0.305346577 | 1.48E-25 |
| CDKN3 | 0.306156424 | 1.09E-25 |
| SFN | 0.303373566 | 0 |
| ARID2 | -0.327788726 | 2.06E-29 |
| HAUS3 | -0.349356695 | 1.94E-33 |
| SLPI | 0.372071288 | 4.96E-38 |
| ATG2B | -0.380370942 | 8.37E-40 |
| COX6B1 | 0.35983528 | 1.64E-35 |
| ATE1 | -0.360886717 | 1.01E-35 |
| CPEB3 | -0.42122333 | 2.62E-49 |
| ASTN2 | -0.314218416 | 4.85E-27 |
| KIF12 | -0.333341425 | 0 |
| IDH2 | 0.371563243 | 0 |
| KISS1 | 0.301156904 | 7.17E-25 |
| ZNF236 | -0.348026863 | 3.51E-33 |
| PREPL | -0.314969846 | 3.61E-27 |
| CPLX1 | -0.422945078 | 9.73E-50 |
| SMYD2 | 0.343064317 | 0 |
| AL390195.2 | -0.355621862 | 1.14E-34 |
| RICTOR | -0.36525488 | 1.30E-36 |
| MEX3A | 0.305799055 | 0 |
| SYBU | -0.388696423 | 1.24E-41 |
| AC109347.1 | -0.323858619 | 1.03E-28 |
| NUDT12 | -0.365028899 | 0 |
| AVEN | 0.355402152 | 1.26E-34 |
| CICP14 | -0.321120167 | 3.12E-28 |
| C12orf66 | -0.33557938 | 7.87E-31 |
| UNC13D | 0.345483734 | 1.08E-32 |
| ZNF620 | -0.371829511 | 5.57E-38 |
| BRD8 | -0.326082494 | 0 |
| AC023024.2 | -0.308012681 | 5.37E-26 |
| AC018752.1 | -0.367170662 | 5.22E-37 |
| TMED8 | -0.346695218 | 6.34E-33 |
| TMEM171 | 0.366625737 | 6.77E-37 |
| ZNRD1ASP | -0.316556946 | 1.93E-27 |
| FNIP1 | -0.393152686 | 1.23E-42 |
| ZNF844 | -0.347860315 | 3.78E-33 |
| ANKRD20A5P | -0.346057964 | 8.40E-33 |
| WDR73 | -0.324652465 | 7.46E-29 |
| SSR2 | 0.426569566 | 1.19E-50 |
| CARD16 | 0.320721628 | 3.66E-28 |
| LINC01133 | 0.358775345 | 2.68E-35 |
| ANKRD26 | -0.302800627 | 3.87E-25 |
| ZNF599 | -0.303206251 | 3.33E-25 |
| GUK1 | 0.335392517 | 0 |
| GRAMD1C | -0.359266349 | 2.14E-35 |
| KLHDC1 | -0.432221753 | 4.23E-52 |
| AC103760.1 | -0.327980196 | 1.90E-29 |
| TBC1D9 | -0.453168098 | 0 |
| AC133552.4 | -0.314945056 | 3.64E-27 |
| STMN1P1 | -0.315394016 | 3.05E-27 |
| KLHDC2 | -0.35440111 | 2.00E-34 |
| TMSB10 | 0.571973545 | 0 |
| ZNF516 | -0.371933192 | 0 |
| GRIK3 | -0.303397783 | 3.10E-25 |
| KIAA0319L | -0.338907244 | 0 |
| MAGI1 | -0.364007242 | 2.34E-36 |
| CHD3 | -0.343542935 | 2.54E-32 |
| ERVE-1 | -0.313968229 | 5.35E-27 |
| GIGYF2 | -0.336786184 | 4.71E-31 |
| UBE3A | -0.303692572 | 2.77E-25 |
| JMJD7-PLA2G4B | -0.307402766 | 6.78E-26 |
| CEP120 | -0.393654712 | 9.49E-43 |
| KLHL11 | -0.437220871 | 2.10E-53 |
| TSTD2 | -0.308537494 | 4.39E-26 |
| MROH8 | -0.418692239 | 1.11E-48 |
| DRC3 | -0.326860514 | 3.02E-29 |
| IMPACT | -0.322401686 | 1.86E-28 |
| CYP4V2 | -0.320163762 | 4.58E-28 |
| AC107027.3 | -0.339196186 | 1.68E-31 |
| MIR641 | -0.318525269 | 8.83E-28 |
| AP1AR | -0.338924261 | 1.88E-31 |
| SLC22A5 | -0.378347326 | 2.29E-39 |
| AL132989.1 | -0.306165346 | 1.09E-25 |
| GLUD1 | -0.306497914 | 0 |
| RPGR | -0.381635702 | 4.45E-40 |
| AC008764.2 | -0.362563972 | 4.60E-36 |
| ZNF91 | -0.359495794 | 1.92E-35 |
| CCDC158 | -0.310802156 | 1.83E-26 |
| NADK2-AS1 | -0.375510012 | 9.27E-39 |
| AL391422.4 | 0.310884494 | 1.78E-26 |
| RAB39B | -0.3376516 | 3.25E-31 |
| AC024575.1 | -0.316320214 | 2.12E-27 |
| DPY19L2P4 | -0.336179362 | 6.10E-31 |
| BCL2L2 | -0.311427749 | 0 |
| AFF4 | -0.318181903 | 0 |
| ERCC6L2 | -0.339507168 | 1.47E-31 |
| SLC1A2 | -0.344305273 | 1.82E-32 |
| CFAP70 | -0.355077607 | 1.47E-34 |
| AC083855.2 | 0.369542656 | 1.68E-37 |
| RFC4 | 0.300981096 | 7.65E-25 |
| CASP14 | 0.321163175 | 3.07E-28 |
| CROCCP3 | -0.347971766 | 3.60E-33 |
| AC018665.1 | -0.33946949 | 1.49E-31 |
| EZH1 | -0.378333472 | 2.30E-39 |
| SLC22A4 | -0.324538042 | 7.81E-29 |
| PTPRN2 | -0.358839481 | 2.61E-35 |
| PCAT6 | 0.370600711 | 0 |
| DEFB1 | 0.336679566 | 4.93E-31 |
| SCAMP1 | -0.33388604 | 0 |
| S100A8 | 0.483989019 | 0 |
| ZZEF1 | -0.339273102 | 1.62E-31 |
| ZNF776 | -0.370321093 | 1.15E-37 |
| IVL | 0.358169849 | 3.55E-35 |
| AC012313.4 | -0.336095355 | 6.32E-31 |
| SYNGAP1 | -0.305911968 | 1.20E-25 |
| CPEB2 | -0.394105656 | 7.50E-43 |
| CASZ1 | -0.343616229 | 2.46E-32 |
| NDUFA6-DT | -0.367291483 | 4.93E-37 |
| ZNF226 | -0.311780621 | 1.26E-26 |
| HDGFL3 | -0.311397609 | 1.46E-26 |
| AC118344.2 | -0.313322184 | 6.88E-27 |
| PPP1R14B | 0.497171258 | 0 |
| MFAP2 | 0.340301292 | 1.04E-31 |
| PRCC | 0.376967944 | 4.53E-39 |
| TATDN2P2 | -0.306207847 | 1.07E-25 |
| SYTL4 | -0.370248931 | 1.20E-37 |
| SLC10A3 | 0.410651356 | 0 |
| RBM34 | 0.301737791 | 5.77E-25 |
| AL021068.1 | -0.345334716 | 1.16E-32 |
| MPHOSPH8 | -0.300871768 | 7.97E-25 |
| CAMTA2 | -0.336559959 | 0 |
| RSU1 | 0.380075869 | 9.70E-40 |
| KCNN4 | 0.319185733 | 6.78E-28 |
| SYN3-AS1 | -0.324653122 | 7.45E-29 |
| P4HTM | -0.39198047 | 2.27E-42 |
| AL035413.1 | -0.346201735 | 7.89E-33 |
| CREBL2 | -0.38396218 | 1.38E-40 |
| ZNF814 | -0.362140985 | 5.61E-36 |
| THSD4 | -0.485476831 | 3.60E-67 |
| ZBTB26 | -0.303603291 | 2.87E-25 |
| ZNF132 | -0.310857133 | 1.80E-26 |
| DYNLT1 | 0.313244082 | 0 |
| CSNK1G3 | -0.362727926 | 4.26E-36 |
| RGS10 | 0.32152272 | 0 |
| RNF141 | -0.309819999 | 2.68E-26 |
| PXDC1 | 0.353760054 | 0 |
| DBNDD2 | -0.345754729 | 9.61E-33 |
| RAB25 | 0.411315429 | 7.03E-47 |
| MYO5C | -0.341924045 | 0 |
| DNAJC18 | -0.319744851 | 5.42E-28 |
| MAP3K12 | -0.34523271 | 1.21E-32 |
| SRGAP1 | -0.377693074 | 3.16E-39 |
| EPG5 | -0.340320871 | 1.03E-31 |
| IKBKE | 0.348169176 | 3.30E-33 |
| NOVA1 | -0.4384092 | 1.02E-53 |
| AC016586.1 | -0.303415107 | 3.08E-25 |
| BCAS3 | -0.324542121 | 7.80E-29 |
| RPP40 | 0.340984502 | 7.74E-32 |
| A2ML1 | 0.339102496 | 1.74E-31 |
| AC007255.1 | -0.337781976 | 3.08E-31 |
| KRT16 | 0.38579225 | 5.46E-41 |
| MPZL1 | 0.342662715 | 0 |
| BECN1 | -0.375161036 | 1.10E-38 |
| AC008124.1 | -0.404217145 | 3.44E-45 |
| MTMR3 | -0.30060041 | 8.82E-25 |
| CYB5R2 | 0.338428909 | 2.33E-31 |
| AL049796.1 | -0.373981215 | 1.96E-38 |
| SYNJ2BP | -0.345403732 | 1.12E-32 |
| DYNLL1 | 0.334050452 | 0 |
| PPP1R14C | 0.372957086 | 3.22E-38 |
| CCDC91 | -0.304157591 | 2.33E-25 |
| PJA2 | -0.338023156 | 0 |
| IFITM3 | 0.331498507 | 0 |
| KIF20A | 0.315080281 | 3.45E-27 |
| XPC-AS1 | -0.327564784 | 2.26E-29 |
| ARFGEF2 | -0.325578051 | 0 |
| CBX2 | 0.355469984 | 1.23E-34 |
| USP8 | -0.310389672 | 2.15E-26 |
| AP000766.1 | -0.344044709 | 2.04E-32 |
| KRT87P | 0.302565231 | 4.23E-25 |
| AC103740.2 | 0.405450581 | 1.76E-45 |
| AC064799.2 | -0.420821194 | 3.30E-49 |
| VGLL1 | 0.359622812 | 1.81E-35 |
| Z97653.2 | -0.326902473 | 2.97E-29 |
| TNFSF9 | 0.31230336 | 1.02E-26 |
| PRORP | -0.308023933 | 5.35E-26 |
| PDCD5 | 0.390458475 | 4.99E-42 |
| PLEKHA3 | -0.30690971 | 8.19E-26 |
| MRPS21 | 0.375670587 | 0 |
| MRNIP | -0.304109356 | 2.37E-25 |
| C2CD5 | -0.322798311 | 1.58E-28 |
| DONSON | 0.31519248 | 3.31E-27 |
| DENND4C | -0.33773039 | 0 |
| RASD2 | 0.319464459 | 6.07E-28 |
| AC025917.1 | -0.32068399 | 3.72E-28 |
| MRPL55 | 0.300237878 | 1.01E-24 |
| NUMA1 | -0.343991814 | 2.09E-32 |
| ETFBKMT | -0.391177024 | 3.44E-42 |
| AC018809.2 | -0.322076632 | 2.12E-28 |
| TP53BP1 | -0.312361786 | 1.00E-26 |
| CCNQ | 0.363899223 | 0 |
| CLSTN2 | -0.401593379 | 1.42E-44 |
| TMPRSS13 | 0.367483939 | 4.50E-37 |
| FTL | 0.330298974 | 0 |
| ADAM15 | 0.414667133 | 0 |
| CALB2 | 0.300117749 | 1.05E-24 |
| ISY1 | 0.34815689 | 3.31E-33 |
| MBD5 | -0.319111772 | 6.98E-28 |
| MMP1 | 0.403854653 | 4.19E-45 |
| WDR17 | -0.374023765 | 1.92E-38 |
| FMN1 | -0.386865267 | 3.16E-41 |
| PARD6B | -0.335892375 | 0 |
| PAXBP1-AS1 | -0.342931208 | 3.32E-32 |
| AC093297.2 | -0.358180256 | 3.53E-35 |
| KLHL28 | -0.303054001 | 3.52E-25 |
| S100A10 | 0.585052513 | 0 |
| ADAL | -0.335532074 | 8.03E-31 |
| DPH6 | -0.311312208 | 1.51E-26 |
| ZKSCAN8 | -0.311899406 | 0 |
| SRARP | -0.352414851 | 4.91E-34 |
| TCF7L1 | 0.314013225 | 5.25E-27 |
| ACADSB | -0.481201865 | 7.35E-66 |
| KRT7 | 0.428630026 | 0 |
| FSCN1 | 0.333474078 | 1.92E-30 |
| ZYX | 0.316585886 | 0 |
| WARS2-AS1 | -0.359622828 | 1.81E-35 |
| NDUFS5 | 0.361887977 | 0 |
| GTF2IP7 | -0.313207467 | 7.20E-27 |
| ARPC2 | 0.315955391 | 2.45E-27 |
| NUDT16 | -0.326880486 | 2.99E-29 |
| ATP7A | -0.305216056 | 1.56E-25 |
| ACER2 | -0.345935354 | 8.87E-33 |
| NBEA | -0.398251865 | 8.43E-44 |
| TPPP | -0.303234478 | 3.29E-25 |
| NUDT8 | 0.314169195 | 4.94E-27 |
| ABCD3 | -0.335362029 | 8.63E-31 |
| TEX14 | -0.321038867 | 3.22E-28 |
| AL445933.2 | -0.3171808 | 1.51E-27 |
| CEP68 | -0.372161907 | 4.74E-38 |
| SLC24A1 | -0.347682286 | 4.09E-33 |
| RNU6-531P | -0.339382347 | 1.55E-31 |
| CACNA1D | -0.344709192 | 1.52E-32 |
| AC099677.1 | -0.355925614 | 9.95E-35 |
| IKZF5 | -0.387814065 | 0 |
| IRAIN | -0.332713388 | 2.64E-30 |
| MIR5581 | -0.374424056 | 1.58E-38 |
| CACNB2 | -0.360769657 | 1.06E-35 |
| THTPA | -0.354029506 | 2.36E-34 |
| AL136115.1 | -0.345781388 | 9.50E-33 |
| AC139149.1 | -0.392342391 | 1.88E-42 |
| TUBB | 0.347581412 | 0 |
| C18orf32 | -0.410484953 | 1.11E-46 |
| FAM222A-AS1 | -0.324106086 | 9.32E-29 |
| PI4KA | -0.344646466 | 1.57E-32 |
| LINC00957 | -0.32728058 | 2.54E-29 |
| NPC2 | 0.314458943 | 0 |
| BBS10 | -0.323955278 | 9.91E-29 |
| C1orf50 | -0.359363939 | 2.04E-35 |
| MAPT | -0.308709195 | 4.11E-26 |
| C17orf75 | -0.313112251 | 7.47E-27 |
| ABHD18 | -0.314685545 | 4.03E-27 |
| PDIA5 | 0.303185651 | 3.35E-25 |
| CCNT1 | -0.324220285 | 8.89E-29 |
| SYDE2 | -0.335432095 | 8.38E-31 |
| DPY19L2P3 | -0.409838807 | 1.59E-46 |
| DDI2 | -0.302142407 | 4.96E-25 |
| RRM2B | -0.307432586 | 6.71E-26 |
| GLCE | -0.330444185 | 0 |
| ABRAXAS1 | -0.342286332 | 4.39E-32 |
| FLAD1 | 0.443179902 | 0 |
| SOX11 | 0.366941276 | 5.82E-37 |
| RPL5P30 | -0.371221907 | 7.48E-38 |
| MTX3 | -0.347186969 | 5.10E-33 |
| ARPC4 | 0.343969822 | 2.11E-32 |
| C2 | 0.309228328 | 3.37E-26 |
| ZADH2 | -0.349037363 | 2.24E-33 |
| TMEM181 | -0.301927346 | 0 |
| ACOT9 | 0.306322249 | 1.02E-25 |
| MIR635 | -0.325598025 | 5.07E-29 |
| TIMM8B | 0.356298311 | 0 |
| UCHL1 | 0.300671606 | 8.58E-25 |
| STON2 | -0.309339228 | 3.23E-26 |
| ZNF568 | -0.352157889 | 5.52E-34 |
| CNTD1 | -0.334707332 | 1.14E-30 |
| H2AX | 0.331463728 | 0 |
| LY6E | 0.302762511 | 0 |
| SDC1 | 0.461593356 | 0 |
| PRKAR1A | -0.314393894 | 0 |
| KIF5C | -0.31974032 | 5.43E-28 |
| CCNDBP1 | -0.335089551 | 9.69E-31 |
| USB1 | 0.431138358 | 8.05E-52 |
| SLC25A46 | -0.317201184 | 1.49E-27 |
| VPS39 | -0.325505631 | 5.26E-29 |
| CRBN | -0.337002159 | 4.29E-31 |
| RASA4CP | -0.32572169 | 4.82E-29 |
| NEBL | -0.300393492 | 0 |
| SOX9 | 0.302902424 | 0 |
| ORM2 | 0.337850421 | 2.99E-31 |
| INCA1 | -0.414520214 | 1.18E-47 |
| GIPC1 | 0.339553893 | 1.44E-31 |
| POLK | -0.312615932 | 9.07E-27 |
| ATP5F1C | 0.312007726 | 0 |
| PAXIP1-AS2 | -0.379481081 | 1.30E-39 |
| SCAPER | -0.345848248 | 9.22E-33 |
| EVL | -0.307846316 | 0 |
| AC092667.1 | -0.437568102 | 1.70E-53 |
| RALGAPA1 | -0.374637307 | 1.42E-38 |
| AL391121.1 | -0.343421663 | 2.68E-32 |
| RNF213-AS1 | -0.437125456 | 2.23E-53 |
| FAM161B | -0.317365587 | 1.40E-27 |
| SREK1 | -0.317184781 | 0 |
| WDR19 | -0.432725079 | 3.13E-52 |
| AKAP10 | -0.337338612 | 0 |
| HSPA8P15 | -0.359196769 | 2.21E-35 |
| SLC39A6 | -0.391948807 | 2.31E-42 |
| ATP5MF | 0.346592758 | 6.64E-33 |
| AKR7L | -0.309336558 | 3.23E-26 |
| TMEM26 | -0.33087089 | 5.72E-30 |
| LINC01801 | -0.364302484 | 2.03E-36 |
| CFL1 | 0.413982563 | 0 |
| KDELR3 | 0.329465569 | 0 |
| S1PR2 | 0.304780592 | 1.84E-25 |
| FOXQ1 | 0.313065722 | 7.61E-27 |
| DNAJC16 | -0.310846902 | 1.80E-26 |
| ACBD4 | -0.301142142 | 7.20E-25 |
| SF3B6 | 0.327364164 | 0 |
| ADAM22 | -0.354240593 | 2.15E-34 |
| HDGF | 0.395038824 | 4.60E-43 |
| ILF2 | 0.417808468 | 0 |
| CELSR1 | -0.325839946 | 0 |
| DNAJB14 | -0.368131586 | 3.30E-37 |
| PER3 | -0.324084506 | 0 |
| AC016590.2 | -0.361011009 | 9.51E-36 |
| DUSP9 | 0.373804054 | 2.13E-38 |
| ULBP2 | 0.388523075 | 1.35E-41 |
| NECTIN4 | 0.429304523 | 0 |
| BRI3 | 0.37636461 | 6.09E-39 |
| CPA4 | 0.357547562 | 4.73E-35 |
| FAAH | -0.30635777 | 0 |
| UFC1 | 0.319121281 | 6.96E-28 |
| HEBP2 | 0.372649796 | 3.74E-38 |
| KIF3A | -0.335969143 | 6.67E-31 |
| ATP1A2 | -0.333145874 | 2.20E-30 |
| AC125603.2 | -0.309888014 | 2.61E-26 |
| DCAF16 | -0.402934894 | 6.88E-45 |
| GRHL3 | 0.340735002 | 8.62E-32 |
| ZNF740 | -0.37626636 | 6.39E-39 |
| S100A6 | 0.30767613 | 6.11E-26 |
| HEPACAM2 | -0.317932361 | 1.12E-27 |
| IL4I1 | 0.303927125 | 2.54E-25 |
| CCNJL | 0.307116925 | 7.56E-26 |
| ATP8B1 | -0.337092045 | 0 |
| EFNA4 | 0.563556548 | 0 |
| FKBP1A | 0.376132254 | 6.83E-39 |
| GIN1 | -0.334131094 | 1.45E-30 |
| IGSF9B | -0.346852287 | 5.92E-33 |
| UGCG | -0.322641652 | 0 |
| MSANTD3 | 0.380812067 | 6.72E-40 |
| AC002128.1 | -0.304604876 | 1.96E-25 |
| AC006059.1 | -0.309040251 | 3.62E-26 |
| NCS1 | 0.314316721 | 4.66E-27 |
| CENPW | 0.43330703 | 2.21E-52 |
| CDH3 | 0.354730965 | 1.72E-34 |
| NXPH4 | 0.369841582 | 1.45E-37 |
| SIAH2-AS1 | -0.311161569 | 1.60E-26 |
| ZNF852 | -0.378086823 | 2.60E-39 |
| SLC5A6 | 0.418204671 | 1.47E-48 |
| RNF122 | 0.31200267 | 1.15E-26 |
| ZNF573 | -0.315094489 | 3.44E-27 |
| MED27 | 0.355213051 | 1.38E-34 |
| CCDC30 | -0.342685865 | 3.69E-32 |
| BAZ2A | -0.305688588 | 0 |
| AC010168.2 | -0.325728732 | 4.80E-29 |
| AC010761.3 | -0.32317641 | 1.36E-28 |
| TLE3 | -0.337681817 | 3.21E-31 |
| AC011815.1 | -0.391534876 | 2.86E-42 |
| PEX12 | -0.346901071 | 5.79E-33 |
| SARM1 | -0.341401514 | 6.46E-32 |
| PITX1 | 0.316466131 | 2.00E-27 |
| NFKBIE | 0.32666847 | 3.27E-29 |
| CRYBG2 | 0.32011947 | 4.67E-28 |
| NUF2 | 0.319624379 | 5.69E-28 |
| JPT1 | 0.388170351 | 0 |
| CBR4 | -0.348430293 | 2.93E-33 |
| SNRPEP2 | 0.307776097 | 5.88E-26 |
| AC008763.1 | -0.35954031 | 1.88E-35 |
| TUBGCP6 | -0.302716739 | 4.00E-25 |
| LETMD1 | -0.405745896 | 1.50E-45 |
| CPEB4 | -0.410249694 | 0 |
| BTRC | -0.345922018 | 8.92E-33 |
| AC012467.1 | -0.330163294 | 7.69E-30 |
| CREBRF | -0.404873928 | 2.41E-45 |
| SLC25A37 | 0.309394116 | 0 |
| REXO2 | 0.321633498 | 2.54E-28 |
| SEC61B | 0.393577396 | 9.88E-43 |
| POLI | -0.377172395 | 4.09E-39 |
| TPTE2P5 | -0.323536814 | 1.17E-28 |
| SCAI | -0.317667849 | 1.24E-27 |
| SNRPE | 0.369972551 | 0 |
| ZNF44 | -0.385393337 | 6.68E-41 |
| ZNF92 | -0.36289719 | 0 |
| H4-16 | -0.404062933 | 3.74E-45 |
| SETBP1 | -0.387780382 | 1.98E-41 |
| KMT2D | -0.344217415 | 1.89E-32 |
| NKAIN1 | -0.313994862 | 5.29E-27 |
| TAF1 | -0.303375078 | 3.12E-25 |
| CASTOR3 | -0.315898513 | 2.50E-27 |
| PIEZO2 | -0.355987109 | 9.68E-35 |
| SAMD8 | -0.372231331 | 4.59E-38 |
| ZFP14 | -0.403552549 | 4.93E-45 |
| SETDB2 | -0.304956666 | 1.72E-25 |
| ULBP3 | 0.341484836 | 6.23E-32 |
| ANKRA2 | -0.307315159 | 7.01E-26 |
| LINC00663 | -0.304409389 | 2.11E-25 |
| MYB | -0.330778918 | 0 |
| AGTR1 | -0.333063394 | 2.28E-30 |
| PLOD1 | 0.358272375 | 0 |
| VPS72 | 0.427545022 | 0 |
| CLIC3 | 0.336501019 | 5.32E-31 |
| UBAP1L | -0.3900613 | 6.13E-42 |
| DNAL1 | -0.339458422 | 1.50E-31 |
| ZNF331 | -0.305727872 | 1.28E-25 |
| GSDMC | 0.487344742 | 9.49E-68 |
| ST14 | 0.348784918 | 2.51E-33 |
| DEFB132 | -0.303305076 | 3.21E-25 |
| SIK3 | -0.301078605 | 7.38E-25 |
| SLC26A1 | -0.34690621 | 5.78E-33 |
| RIC8B | -0.328667894 | 1.43E-29 |
| AC004771.2 | -0.330889618 | 5.68E-30 |
| AC053513.2 | -0.345495187 | 1.08E-32 |
| MAGI2 | -0.348018845 | 3.52E-33 |
| EFNA1 | 0.343985364 | 0 |
| APH1A | 0.316236229 | 0 |
| MTRES1 | 0.307037696 | 7.80E-26 |
| GPR135 | -0.328873892 | 1.31E-29 |
| MIS18A | 0.302392902 | 4.51E-25 |
| TOM1L1 | -0.312118465 | 1.10E-26 |
| HNRNPA1P14 | -0.308476893 | 4.50E-26 |
| PIGV | -0.319985294 | 4.92E-28 |
| BDP1 | -0.325923856 | 4.43E-29 |
| RAB37 | -0.31192598 | 1.19E-26 |
| NKX1-2 | 0.309003924 | 3.67E-26 |
| AC004231.1 | 0.300395181 | 9.51E-25 |
| CASKIN1 | -0.330608944 | 6.38E-30 |
| Z68871.1 | -0.314034164 | 5.21E-27 |
| EIF4BP7 | -0.308832784 | 3.92E-26 |
| GUF1 | -0.320736854 | 3.64E-28 |
| GCC2 | -0.341991487 | 5.00E-32 |
| ZNF780A | -0.331567996 | 4.28E-30 |
| ACTBP2 | 0.301415069 | 6.51E-25 |
| CKAP4 | 0.336593991 | 0 |
| ZBTB16 | -0.315474904 | 2.96E-27 |
| LINC01503 | 0.337771534 | 3.09E-31 |
| TRIM16 | 0.314113975 | 5.05E-27 |
| OLFM2 | 0.311400553 | 0 |
| TOGARAM1 | -0.364538953 | 1.82E-36 |
| TEX52 | -0.368349716 | 2.97E-37 |
| CHD1 | -0.305658803 | 1.32E-25 |
| AC087163.1 | -0.357187011 | 5.58E-35 |
| EIF3J-DT | -0.439907432 | 4.10E-54 |
| IGSF9 | 0.30050837 | 9.12E-25 |
| ZNF695 | 0.320150389 | 4.61E-28 |
| ABCA5 | -0.351292032 | 8.15E-34 |
| AC096586.2 | -0.344157979 | 1.94E-32 |
| PADI2 | 0.377625836 | 0 |
| EIF4B | -0.377694217 | 0 |
| LINC02004 | 0.305255322 | 1.54E-25 |
| IQCH | -0.386989785 | 2.96E-41 |
| ABCC8 | -0.304054921 | 2.42E-25 |
| AC092718.4 | 0.445239665 | 1.53E-55 |
| WDFY3 | -0.36805352 | 3.43E-37 |
| INTU | -0.360448596 | 1.24E-35 |
| IL32 | 0.367704382 | 4.05E-37 |
| CTC1 | -0.326184457 | 3.98E-29 |
| TIGD4 | -0.305121419 | 1.62E-25 |
| RALGAPA1P1 | -0.347727264 | 4.01E-33 |
| PANK3 | -0.378072677 | 2.62E-39 |
| ZNF141 | -0.423798573 | 5.94E-50 |
| FRAT1 | -0.312115453 | 1.10E-26 |
| TNFRSF12A | 0.423271299 | 0 |
| ATP6V1C2 | 0.31195447 | 1.17E-26 |
| MYL12A | 0.306044282 | 0 |
| MAPT-IT1 | -0.364404409 | 1.94E-36 |
| TREM1 | 0.32411295 | 9.29E-29 |
| PSMD4 | 0.444877301 | 0 |
| ZNF516-DT | -0.300601961 | 8.81E-25 |
| AC037459.4 | -0.336380676 | 5.60E-31 |
| AC130650.2 | -0.321663726 | 2.51E-28 |
| TCP11L2 | -0.36493145 | 1.51E-36 |
| WDR35 | -0.33744322 | 3.56E-31 |
| SOCS1 | 0.329932063 | 8.46E-30 |
| UBE2C | 0.350609841 | 0 |
| KANSL1 | -0.343946754 | 2.13E-32 |
| SACM1L | -0.333491322 | 1.91E-30 |
| MGST3 | 0.510901751 | 2.32E-75 |
| NEDD4L | -0.383963429 | 1.38E-40 |
| USP32 | -0.304765806 | 0 |
| GPR19 | 0.327356853 | 2.46E-29 |
| SMIM14 | -0.312885735 | 8.16E-27 |
| ZNF25 | -0.302653988 | 4.09E-25 |
| DRAIC | -0.30388965 | 2.57E-25 |
| ELOVL2 | -0.320893092 | 3.42E-28 |
| AL024507.2 | 0.326024732 | 4.25E-29 |
| GMDS-DT | -0.363088913 | 3.60E-36 |
| AC124944.3 | -0.300770886 | 8.27E-25 |
| TEAD2 | 0.339320911 | 0 |
| ZSWIM6 | -0.366499943 | 7.18E-37 |
| PARP11 | -0.350613297 | 1.11E-33 |
| TRAPPC11 | -0.309097177 | 3.54E-26 |
| TRIM47 | 0.345881988 | 9.08E-33 |
| AC008663.1 | -0.322384922 | 1.87E-28 |
| NIPBL-DT | -0.353890642 | 2.52E-34 |
| AL357054.2 | -0.329070192 | 1.21E-29 |
| SF3B4 | 0.488382441 | 0 |
| RN7SL381P | -0.32160731 | 2.56E-28 |
| FAM198B-AS1 | -0.361788915 | 6.61E-36 |
| TAPT1-AS1 | -0.321838729 | 2.34E-28 |
| HTT | -0.355562894 | 1.17E-34 |
| AC010203.1 | -0.360046544 | 1.49E-35 |
| DCAF11 | -0.358654828 | 2.84E-35 |
| AL133387.1 | -0.349842681 | 1.56E-33 |
| S100A2 | 0.347924324 | 3.68E-33 |
| ZNF875 | -0.331504871 | 4.39E-30 |
| MPZL2 | 0.426047776 | 0 |
| RUFY2 | -0.312241247 | 1.05E-26 |
| AC008393.1 | -0.320128173 | 4.65E-28 |
| GNPDA2 | -0.315100576 | 3.43E-27 |
| GABPB1-AS1 | -0.301829803 | 5.57E-25 |
| N4BP2 | -0.402454285 | 8.92E-45 |
| ZNF808 | -0.33425811 | 1.38E-30 |
| SNHG14 | -0.381147303 | 5.68E-40 |
| CHD2 | -0.367050096 | 5.53E-37 |
| SLC35E2B | -0.349550916 | 0 |
| HOMER3 | 0.38336977 | 1.86E-40 |
| PELATON | 0.394583098 | 5.84E-43 |
| FSCN2 | -0.300206547 | 1.02E-24 |
| PCMTD2 | -0.404237354 | 3.40E-45 |
| P2RY6 | 0.311969079 | 1.17E-26 |
| DMAC2L | -0.303847075 | 2.61E-25 |
| GNAS | 0.313782204 | 0 |
| HECTD4 | -0.381579113 | 4.58E-40 |
| MYBL2 | 0.339922156 | 1.23E-31 |
| AL078582.2 | -0.407743096 | 5.04E-46 |
| TXNDC17 | 0.36205328 | 5.84E-36 |
| AXIN2 | -0.303324986 | 3.18E-25 |
| PRIMPOL | -0.326586488 | 3.38E-29 |
| PPP1R14BP3 | 0.502923147 | 1.02E-72 |
| MSRB2 | 0.318543415 | 8.76E-28 |
| CHM | -0.342928141 | 3.32E-32 |
| SALL2 | -0.303668365 | 2.80E-25 |
| KCNG1 | 0.345755119 | 9.61E-33 |
| SCNM1 | 0.569861048 | 2.92E-97 |
| USF1 | 0.342810897 | 0 |
| STXBP4 | -0.31717973 | 1.51E-27 |
| EDEM1 | -0.306697303 | 0 |
| PTTG1 | 0.358780804 | 2.68E-35 |
| HOXC13-AS | 0.353643583 | 2.82E-34 |
| TTC6 | -0.366809983 | 6.20E-37 |
| TMEM79 | 0.364209285 | 2.12E-36 |
| PRSS27 | 0.331438446 | 4.51E-30 |
| FBP1 | -0.319815223 | 0 |
| ZNF791 | -0.324469029 | 8.03E-29 |
| CDCA7 | 0.315179793 | 3.32E-27 |
| TMSB4X | 0.36244591 | 0 |
| ZNF792 | -0.319275048 | 6.54E-28 |
| TRIM66 | -0.374592281 | 1.45E-38 |
| EID1 | -0.341543714 | 0 |
| AC005225.4 | -0.325767742 | 4.73E-29 |
| AP2S1 | 0.315292468 | 0 |
| S100A7 | 0.411047813 | 8.15E-47 |
| ZSWIM5 | -0.398442356 | 7.62E-44 |
| ZNF587 | -0.406337032 | 0 |
| RAD17P1 | -0.326128451 | 4.08E-29 |
| PARL | 0.323724537 | 1.09E-28 |
| SORCS1 | -0.326810257 | 3.08E-29 |
| ZBTB40 | -0.365485242 | 1.16E-36 |
| RNF157 | -0.340730313 | 8.64E-32 |
| MIR3671 | -0.301561853 | 6.16E-25 |
| LINC01488 | -0.31027858 | 2.25E-26 |
| APOC1 | 0.306393614 | 9.97E-26 |
| EN1 | 0.417802868 | 1.84E-48 |
| NAPB | -0.371248457 | 7.38E-38 |
| AC092718.5 | -0.336892851 | 4.50E-31 |
| CTR9 | -0.312177989 | 1.08E-26 |
| TBX3 | -0.348140462 | 0 |
| RFC1 | -0.324617773 | 7.56E-29 |
| AC024075.3 | -0.370924555 | 8.63E-38 |
| CTSV | 0.348534012 | 2.80E-33 |
| ST7L | -0.33586026 | 6.99E-31 |
| TSSK4 | -0.346973074 | 5.61E-33 |
| MAML3 | -0.357481258 | 4.87E-35 |
| AFF1 | -0.303404245 | 3.09E-25 |
| MOB1B | -0.320972538 | 3.31E-28 |
| ATP5MC1P4 | 0.325534401 | 5.20E-29 |
| VPS13C | -0.339862293 | 1.26E-31 |
| DPY19L4 | -0.30196653 | 5.30E-25 |
| CERS6 | -0.335263614 | 9.00E-31 |
| TRAK1 | -0.368565618 | 2.68E-37 |
| CSTB | 0.513621623 | 0 |
| ZFX | -0.331607872 | 4.21E-30 |
| PIK3C2A | -0.352899776 | 3.94E-34 |
| SERPINH1 | 0.428576262 | 3.66E-51 |
| PREX1 | -0.331000547 | 0 |
| FAM160A1-DT | -0.302221568 | 4.81E-25 |
| CD58 | 0.309902652 | 2.60E-26 |
| ANXA3 | 0.31965261 | 5.63E-28 |
| PLAUR | 0.468970136 | 3.27E-62 |
| AC015813.5 | -0.31704276 | 1.59E-27 |
| CT62 | -0.360462668 | 1.23E-35 |
| SPSB1 | 0.316684846 | 0 |
| STK32B | -0.316758255 | 1.78E-27 |
| TRPM7 | -0.34555604 | 0 |
| ALG13-AS1 | -0.332610554 | 2.76E-30 |
| ABCG2 | -0.302402383 | 4.50E-25 |
| AC080112.3 | -0.316050284 | 2.36E-27 |
| PEX5L | -0.410335912 | 1.21E-46 |
| SNORA11 | -0.32709112 | 2.74E-29 |
| VEZF1 | -0.321523595 | 0 |
| ANKRD61 | -0.311296968 | 1.51E-26 |
| ANKS1B | -0.362876307 | 3.97E-36 |
| CDS2 | -0.311252405 | 0 |
| TRAPPC8 | -0.328361379 | 1.62E-29 |
| PRDX6 | 0.386795938 | 0 |
| LRBA | -0.450663538 | 0 |
| ZNF609 | -0.312804843 | 8.43E-27 |
| FRY | -0.381135618 | 5.71E-40 |
| APPL2 | -0.305620001 | 0 |
| RPS6KA5 | -0.308135075 | 5.12E-26 |
| RELL1 | -0.371212174 | 7.51E-38 |
| HACL1 | -0.300050914 | 1.08E-24 |
| SUGT1P3 | -0.405892123 | 1.39E-45 |
| WDR6 | -0.326885071 | 0 |
| LCN2 | 0.397952638 | 9.88E-44 |
| NEURL1 | -0.311470575 | 1.42E-26 |
| KCNK1 | 0.303235583 | 3.29E-25 |
| XPC | -0.36739554 | 4.69E-37 |
| FBXL17 | -0.366018396 | 9.03E-37 |
| UBE2E3 | 0.374481416 | 1.53E-38 |
| GPR139 | -0.312982523 | 7.86E-27 |
| RAD50 | -0.362148439 | 5.59E-36 |
| ATP5MC3 | 0.311609023 | 1.34E-26 |
| DPY19L2 | -0.311558991 | 1.37E-26 |
| FAM219B | -0.34340609 | 2.70E-32 |
| COTL1 | 0.325864256 | 0 |
| AL121672.1 | -0.412134885 | 4.46E-47 |
| DCTN4 | -0.337804782 | 0 |
| AMIGO1 | -0.303261683 | 3.26E-25 |
| WNK4 | -0.316741811 | 1.79E-27 |
| RPL39L | 0.371616261 | 0 |
| TUBB6 | 0.356265745 | 8.52E-35 |
| DNALI1 | -0.320374101 | 0 |
| PLAAT1 | 0.326315795 | 3.77E-29 |
| PHC3 | -0.32223738 | 1.99E-28 |
| THAP5 | -0.332752372 | 2.60E-30 |
| HTR7P1 | -0.357686311 | 4.44E-35 |
| DUBR | -0.36404905 | 2.29E-36 |
| BOD1L1 | -0.38319497 | 2.03E-40 |
| YTHDC2 | -0.329419619 | 1.05E-29 |
| LRRC49 | -0.305101005 | 1.63E-25 |
| ACTG1P14 | 0.306444215 | 9.77E-26 |
| LMX1B | -0.326697392 | 3.23E-29 |
| ARMT1 | -0.340349402 | 0 |
| AC093512.2 | -0.305249058 | 1.54E-25 |
| PDZK1IP1 | 0.367337466 | 4.82E-37 |
| RERE | -0.346994289 | 5.55E-33 |
| ELAPOR2 | -0.39512566 | 4.39E-43 |
| AL691432.1 | -0.340728687 | 8.64E-32 |
| TALDO1 | 0.301526311 | 0 |
| AMN1 | -0.360358739 | 1.29E-35 |
| NMI | 0.386865397 | 3.16E-41 |
| INAVA | 0.391470232 | 2.96E-42 |
| B4GALT3 | 0.336216605 | 0 |
| TSPOAP1 | -0.306512819 | 9.52E-26 |
| ZNF484 | -0.304647245 | 1.93E-25 |
| TRPV6 | 0.349921655 | 1.51E-33 |
| AC036108.1 | -0.40228748 | 9.76E-45 |
| HERC1 | -0.371849018 | 5.52E-38 |
| PHF7 | -0.366431885 | 7.42E-37 |
| RFC2 | 0.321424893 | 0 |
| DUSP23 | 0.378154625 | 2.52E-39 |
| AL031846.2 | -0.380379839 | 8.33E-40 |
| CACNA2D2 | -0.416457864 | 3.95E-48 |
| MAPKBP1 | -0.310607572 | 1.98E-26 |
| C1orf198 | 0.397429324 | 1.30E-43 |
| RGS22 | -0.386398581 | 4.01E-41 |
| CXCL16 | 0.312953175 | 7.95E-27 |
| Z99572.1 | -0.304821304 | 1.81E-25 |
| TSEN15 | 0.358097947 | 3.67E-35 |
| AC233300.1 | -0.332302611 | 3.14E-30 |
| CCDC170 | -0.364035595 | 2.30E-36 |
| BCL2 | -0.542952525 | 1.00E-86 |
| RHOG | 0.380217602 | 9.04E-40 |
| NCK2 | 0.322377404 | 0 |
| SLURP1 | 0.344997498 | 1.34E-32 |
| S100A4 | 0.334567785 | 0 |
| HAX1 | 0.338805039 | 1.98E-31 |
| TAGLN2 | 0.567512851 | 0 |
| CHD6 | -0.410935468 | 8.67E-47 |
| RNF123 | -0.314605552 | 4.16E-27 |
| AL021368.2 | -0.319218655 | 6.69E-28 |
| COA6 | 0.36533262 | 1.25E-36 |
| SH3BGRL3 | 0.486219448 | 0 |
| GLIPR1L2 | -0.339497638 | 1.47E-31 |
| ZNF106 | -0.328998817 | 1.25E-29 |
| TPSG1 | -0.31364101 | 6.08E-27 |
| ZNF552 | -0.30888525 | 0 |
| NBR1 | -0.411451021 | 0 |
| LINC00674 | -0.312677561 | 0 |
| TUBA1C | 0.315693422 | 2.71E-27 |
| ZNF571 | -0.325407672 | 5.48E-29 |
| CD82 | 0.344133074 | 0 |
| RBM38 | 0.354488485 | 1.92E-34 |
| CEP290 | -0.362679985 | 4.36E-36 |
| MDM2 | -0.359775536 | 1.69E-35 |
| SMCR8 | -0.362478676 | 4.79E-36 |
| RAB3IL1 | 0.307209012 | 7.30E-26 |
| PCNX4 | -0.312606864 | 9.10E-27 |
| TTYH3 | 0.352428325 | 4.88E-34 |
| PHLDA2 | 0.342326463 | 0 |
| PLA2G12A | -0.421917659 | 1.76E-49 |
| TXN | 0.370802813 | 0 |
| ERCC5 | -0.301288445 | 6.82E-25 |
| ERBB4 | -0.386776226 | 3.31E-41 |
| DPP8 | -0.329286102 | 1.11E-29 |
| TNRC6C | -0.361744419 | 6.75E-36 |
| TMEM258 | 0.393772521 | 8.92E-43 |
| HSPA12A | -0.304151469 | 2.33E-25 |
| STAM2 | -0.300825885 | 8.11E-25 |
| JADE1 | -0.375232757 | 1.06E-38 |
| MOAP1 | -0.417684374 | 1.97E-48 |
| ATP5MD | 0.305122811 | 1.61E-25 |
| CHIC1 | -0.325556325 | 5.15E-29 |
| GRIA1 | -0.409441578 | 1.98E-46 |
| LYPD6 | -0.389728742 | 7.28E-42 |
| CPB1 | -0.319216157 | 6.70E-28 |
| TOMM20L | -0.369943902 | 1.38E-37 |
| EPB41 | -0.405926371 | 1.36E-45 |
| KRT6A | 0.309014498 | 3.66E-26 |
| MRPS31P4 | -0.324674076 | 7.39E-29 |
| LRRC37A16P | -0.328543693 | 1.51E-29 |
| RCN3 | 0.30998274 | 0 |
| DMXL1 | -0.436375872 | 3.50E-53 |
| CLCN3 | -0.328556743 | 1.50E-29 |
| ABLIM3 | -0.300411325 | 9.46E-25 |
| ZFYVE28 | -0.306428206 | 9.83E-26 |
| PFDN2 | 0.522420736 | 0 |
| LIMK1 | 0.358110041 | 0 |
| DPY19L1P1 | -0.406078267 | 1.25E-45 |
| KDM4B | -0.361494138 | 0 |
| AL356417.2 | 0.318994919 | 7.32E-28 |
| XBP1 | -0.350754983 | 0 |
| FBXO36 | -0.337763015 | 3.10E-31 |
| LONRF2 | -0.410165555 | 1.33E-46 |
| ABRACL | 0.492588899 | 0 |
| RAP2B | 0.372599266 | 3.84E-38 |
| DEDD | 0.443879902 | 3.56E-55 |
| SNAI1 | 0.371656328 | 6.06E-38 |
| CFAP97 | -0.308523937 | 4.41E-26 |
| CEP44 | -0.346490857 | 6.94E-33 |
| LYPD6B | -0.363267175 | 3.31E-36 |
| NUDT6 | -0.359721388 | 1.73E-35 |
| CLASP2 | -0.374848165 | 0 |
| CARF | -0.398771643 | 6.40E-44 |
| SECISBP2L | -0.329305832 | 1.10E-29 |
| AL136981.2 | -0.358227057 | 3.46E-35 |
| SENP7 | -0.320952997 | 3.34E-28 |
| CENPC | -0.349810935 | 1.58E-33 |
| ZNRF2P2 | -0.386774921 | 3.31E-41 |
| AC095057.3 | -0.334455347 | 1.27E-30 |
| POC1B-AS1 | -0.41520177 | 8.02E-48 |
| RAB7B | 0.332976654 | 2.37E-30 |
| TMEM81 | 0.31315951 | 7.34E-27 |
| KRT86 | 0.373751663 | 2.19E-38 |
| PHF21B | -0.357345881 | 5.19E-35 |
| TNRC18P1 | -0.370434357 | 1.09E-37 |
| PSMB4 | 0.564556478 | 0 |
| LINC00472 | -0.313485005 | 6.46E-27 |
| SLC25A38 | -0.301095148 | 7.33E-25 |
| PPP1R12A-AS1 | -0.309375377 | 3.18E-26 |
| DBI | 0.401108708 | 1.84E-44 |
| FABP5 | 0.323839764 | 1.04E-28 |
| PIGL | -0.32486622 | 6.83E-29 |
| SMARCC2 | -0.334713405 | 1.14E-30 |
| CA9 | 0.402775607 | 7.50E-45 |
| AC079296.1 | -0.361124667 | 9.02E-36 |
| AC027644.1 | -0.36821861 | 3.17E-37 |
| AC253536.3 | -0.354717178 | 1.73E-34 |
| COLGALT1 | 0.312589893 | 0 |
| NRBP1 | 0.320187259 | 4.54E-28 |
| SMCO4 | 0.399639177 | 0 |
| AC105328.1 | -0.408110265 | 4.12E-46 |
| BCL2A1 | 0.324717812 | 7.26E-29 |
| STC2 | -0.306436292 | 0 |
| LINC01521 | -0.336767307 | 4.75E-31 |
| AC005154.2 | -0.313785928 | 5.74E-27 |
| VIM-AS1 | 0.305591702 | 1.35E-25 |
| CEBPB | 0.461523529 | 4.62E-60 |
| ASS1 | 0.380213024 | 0 |
| AKR7A3 | -0.315009716 | 3.55E-27 |
| VKORC1 | 0.325677914 | 4.90E-29 |
| GLUD1P3 | -0.328446183 | 1.57E-29 |
| PROM1 | 0.319943967 | 5.01E-28 |
| STX6 | 0.314380194 | 4.55E-27 |
| VPS13B | -0.357659983 | 4.49E-35 |
| RAD17 | -0.332847099 | 2.50E-30 |
| CENPA | 0.306421815 | 9.86E-26 |
| ZNRF3 | -0.348551337 | 2.78E-33 |
| SLC30A9 | -0.322649905 | 1.68E-28 |
| ZNF586 | -0.303210194 | 3.32E-25 |
| SPATA5 | -0.335657843 | 7.61E-31 |
| RAET1L | 0.463237927 | 1.50E-60 |
| PFKP | 0.344324588 | 0 |
| ZBTB14 | -0.308895835 | 3.83E-26 |
| PRMT9 | -0.300242133 | 1.01E-24 |
| LAD1 | 0.470157037 | 1.47E-62 |
| RETREG1 | -0.355003087 | 1.52E-34 |
| PHGDH | 0.323337636 | 1.27E-28 |
| AC131212.2 | -0.331736645 | 3.98E-30 |
| PURA | -0.353699022 | 2.75E-34 |
| AURKB | 0.335167916 | 0 |
| CCT3 | 0.32756442 | 0 |
| MRPS30-DT | -0.350692211 | 1.07E-33 |
| HCAR1 | -0.407152153 | 6.97E-46 |
| KIAA0232 | -0.346794104 | 0 |
| MASP2 | -0.418077789 | 1.58E-48 |
| FOSL1 | 0.386275183 | 4.27E-41 |
| DENND5B | -0.335449043 | 8.32E-31 |
| NXPE3 | -0.310364605 | 2.17E-26 |
| RPH3AL | -0.33572021 | 7.42E-31 |
| MARCO | 0.319142114 | 6.90E-28 |
| DGKE | -0.439674857 | 4.72E-54 |
| LIAS | -0.350462531 | 1.18E-33 |
| C1orf116 | 0.316535715 | 1.95E-27 |
| MED13 | -0.319661549 | 5.61E-28 |
| AC010326.3 | -0.326625499 | 3.32E-29 |
| AC092645.1 | -0.335146608 | 9.46E-31 |
| LZTFL1 | -0.320385468 | 4.19E-28 |
| ALG10B | -0.360255889 | 1.35E-35 |
| CDCA2 | 0.307745461 | 5.95E-26 |
| MARCHF8 | -0.314800619 | 3.86E-27 |
| AC106795.2 | -0.307720879 | 6.01E-26 |
| ATP6V1F | 0.35479399 | 0 |
| SMPD3 | -0.329083488 | 1.20E-29 |
| AHCYL2 | -0.340211805 | 1.08E-31 |
| TPRG1 | -0.301856954 | 5.52E-25 |
| AC027607.1 | -0.311140529 | 1.61E-26 |
| ESR1 | -0.384855193 | 0 |
| AL135818.2 | -0.324479821 | 8.00E-29 |
| RSBN1L | -0.319472707 | 6.05E-28 |
| UBE2FP3 | -0.315182269 | 3.32E-27 |
| C5orf46 | 0.407034714 | 7.43E-46 |
| NOP10 | 0.346223813 | 0 |
| TMEM45A | 0.330384144 | 7.01E-30 |
| PSMG1 | 0.318522612 | 0 |
| AC011477.1 | -0.346615874 | 6.57E-33 |
| ACVR2B | -0.329182166 | 1.16E-29 |
| AL357054.4 | -0.329041144 | 1.23E-29 |
| RANP4 | -0.307046954 | 7.77E-26 |
| RERG-AS1 | -0.385366362 | 6.78E-41 |
| AC108673.2 | 0.358139864 | 3.60E-35 |
| CBR3 | 0.393187708 | 0 |
| ANKRD12 | -0.333417459 | 1.97E-30 |
| FLNB | -0.375047108 | 1.16E-38 |
| CDC20 | 0.356908903 | 6.34E-35 |
| NEK4 | -0.337353381 | 3.70E-31 |
| GTF2IP23 | -0.331978222 | 3.60E-30 |
| ARHGEF6 | -0.313891151 | 0 |
| SNRPF | 0.350745221 | 1.04E-33 |
| BBIP1 | -0.399060485 | 5.49E-44 |
| AC018926.1 | -0.307623057 | 6.23E-26 |
| AC015849.3 | -0.338878494 | 1.92E-31 |
| AHNAK | -0.356315065 | 0 |
| TSHZ1 | -0.413347306 | 2.27E-47 |
| RTL8A | 0.318890337 | 0 |
| ANXA2 | 0.37790105 | 0 |
| KDM7A-DT | 0.347880355 | 3.75E-33 |
| FAM102B | -0.315772172 | 2.63E-27 |
| SKA3 | 0.320926495 | 0 |
| NUDT1 | 0.35271067 | 4.30E-34 |
| LINC01615 | 0.335181746 | 9.32E-31 |
| EFCAB13 | -0.358794451 | 2.66E-35 |
| TMEM170B | -0.321000028 | 3.28E-28 |
| MYT1 | -0.357388201 | 5.09E-35 |
| AC093297.1 | -0.352752087 | 4.22E-34 |
| C12orf76 | -0.361660165 | 7.02E-36 |
| CCND1 | -0.336882687 | 0 |
| PGPEP1 | -0.355786928 | 1.06E-34 |
| AC083798.2 | -0.314245369 | 4.80E-27 |
| AC122129.1 | -0.313183472 | 7.27E-27 |
| TYW3 | -0.327559166 | 2.26E-29 |
| ELOCP19 | -0.305827579 | 1.24E-25 |
| AKAP11 | -0.30295953 | 3.65E-25 |
| AC009716.1 | -0.307980625 | 5.44E-26 |
| S100A9 | 0.471935092 | 4.41E-63 |
| ZNRF2P1 | -0.382414038 | 3.01E-40 |
| ZC3H6 | -0.391043832 | 3.69E-42 |
| ZNF780B | -0.327616507 | 2.21E-29 |
| WDR7 | -0.325602145 | 5.06E-29 |
| KRT81 | 0.325324142 | 5.67E-29 |
| LBP | 0.310391018 | 2.15E-26 |
| SP6 | 0.330637851 | 6.31E-30 |
| MFSD2A | 0.318816935 | 7.86E-28 |
| MRPL47 | 0.435030445 | 7.88E-53 |
| VASP | 0.34680763 | 6.03E-33 |
| SNX13 | -0.301722127 | 5.80E-25 |
| RSBN1 | -0.344732634 | 1.51E-32 |
| PPM1A | -0.301081284 | 7.37E-25 |
| S100A3 | 0.332789464 | 2.56E-30 |
| HAPLN3 | 0.331860141 | 3.78E-30 |
| PPIC | 0.3469867 | 0 |
| LANCL1 | -0.311869942 | 1.21E-26 |
| SEPSECS | -0.404169557 | 3.53E-45 |
| AC073107.1 | -0.309762064 | 2.74E-26 |
| NRIP1 | -0.347136746 | 5.21E-33 |
| ZNF555 | -0.30372422 | 2.74E-25 |
| RTL8C | 0.334945559 | 1.03E-30 |
| AC004522.2 | -0.3235405 | 1.17E-28 |
| MED13L | -0.458782589 | 0 |
| VAMP2 | -0.349020824 | 2.26E-33 |
| TMEM51 | 0.324783615 | 7.07E-29 |
| KBTBD4 | -0.304472254 | 2.06E-25 |
| PERP | 0.322680733 | 0 |
| NAV3 | -0.385995876 | 4.92E-41 |
| SLC35E2A | -0.412102358 | 4.54E-47 |
| GLMP | 0.349751584 | 0 |
| ADA | 0.36079122 | 1.05E-35 |
| GLIPR2 | 0.334858757 | 0 |
| HIPK1 | -0.315269026 | 3.21E-27 |
| SLC4A11 | 0.314833265 | 3.81E-27 |
| SLTM | -0.312468449 | 9.61E-27 |
| BTG3 | 0.356185488 | 8.84E-35 |
| C9orf64 | -0.311279534 | 1.52E-26 |
| ATP9B | -0.335658056 | 7.61E-31 |
| ZNF483 | -0.444000417 | 3.31E-55 |
| FBXL5 | -0.348541961 | 0 |
| KANSL3 | -0.317913551 | 1.13E-27 |
| RPP38 | 0.343967912 | 2.11E-32 |
| PAN2 | -0.394916142 | 4.90E-43 |
| ZNF56 | -0.302627142 | 4.13E-25 |
| HIVEP3 | -0.317437421 | 1.36E-27 |
| SYNGR1 | -0.310239759 | 2.28E-26 |
| DENND4A | -0.34218898 | 0 |
| INSYN2A | -0.388640836 | 1.27E-41 |
| MEAK7 | 0.309959303 | 2.54E-26 |
| FEM1B | -0.32543302 | 0 |
| BANF1 | 0.306807529 | 0 |
| SNRPGP2 | 0.424335584 | 4.35E-50 |
| TTC28 | -0.308593456 | 4.30E-26 |
| ENTPD5 | -0.302098947 | 5.04E-25 |
| ABCG1 | -0.33028314 | 7.31E-30 |
| LINC01671 | 0.324312293 | 8.57E-29 |
| HMGA1 | 0.35419026 | 0 |
| TBC1D14 | -0.38317072 | 2.06E-40 |
| ARF1 | 0.337104428 | 0 |
| LIFR | -0.323397936 | 1.24E-28 |
| LGALS1 | 0.355312465 | 0 |
| NRBF2 | 0.300867946 | 7.98E-25 |
| LAGE3 | 0.371471081 | 6.63E-38 |
| DNAJC12 | -0.355615484 | 0 |
| TRIM23 | -0.380085048 | 0 |
| PSMG3 | 0.365472726 | 1.17E-36 |
| TMEM192 | -0.34421374 | 1.89E-32 |
| MRTFB | -0.384373229 | 1.12E-40 |
| TINCR | 0.319248527 | 6.61E-28 |
| NUBPL | -0.353141925 | 3.54E-34 |
| KRT83 | 0.30204609 | 5.14E-25 |
| PPP4C | 0.312422097 | 0 |
| CST6 | 0.329386396 | 1.06E-29 |
| AC012313.5 | -0.329990251 | 8.26E-30 |
| ASB16 | -0.362581275 | 4.56E-36 |
| PISD | -0.3557084 | 1.10E-34 |
| MYRIP | -0.394618577 | 5.73E-43 |
| CRACR2A | -0.343634702 | 2.44E-32 |
| AL135786.1 | -0.371731583 | 5.84E-38 |
| NEK9 | -0.331575509 | 0 |
| C5AR2 | -0.32620126 | 3.96E-29 |
| RAB11FIP2 | -0.302244914 | 4.77E-25 |
| LINC01842 | 0.303272588 | 3.24E-25 |
| ATP8A1 | -0.30242171 | 4.47E-25 |
| DELE1 | -0.31813724 | 0 |
| PLCD4 | -0.394794572 | 5.22E-43 |
| DNAJC24 | -0.311118908 | 1.62E-26 |
| AC104596.1 | -0.301797146 | 5.64E-25 |
| PRRX2 | 0.360224018 | 1.37E-35 |
| CNNM3 | -0.372137018 | 4.80E-38 |
| ZNF442 | -0.31566386 | 2.75E-27 |
| TMEM92 | 0.312872836 | 8.21E-27 |
| ARSG | -0.46771501 | 7.61E-62 |
| AC083906.3 | -0.352320081 | 5.13E-34 |
| SNRPGP4 | -0.338670702 | 2.10E-31 |
| SPEF2 | -0.31914165 | 6.90E-28 |
| ZNF540 | -0.413952438 | 1.62E-47 |
| MZF1 | -0.304287857 | 2.21E-25 |
| HEATR5B | -0.367619047 | 4.22E-37 |
| MACF1 | -0.355689765 | 0 |
| AFF3 | -0.479381329 | 2.62E-65 |
| SLC7A5 | 0.301620986 | 0 |
| MRPL9 | 0.437090836 | 2.27E-53 |
| TBC1D12 | -0.373458075 | 2.53E-38 |
| GSAP | -0.314422102 | 4.47E-27 |
| CAPG | 0.357346888 | 0 |
| AL353748.2 | -0.327604711 | 2.22E-29 |
| ZNF441 | -0.367410729 | 4.66E-37 |
| MIR29B2CHG | -0.368062642 | 3.41E-37 |
| NRXN3 | -0.337828204 | 3.02E-31 |
| KLHDC7B-DT | 0.367097619 | 5.41E-37 |
| AC090246.1 | -0.303913263 | 2.55E-25 |
| DNAJC14 | -0.325892687 | 4.49E-29 |
| BAZ2B | -0.336637578 | 5.02E-31 |
| LINC01956 | 0.300575885 | 8.90E-25 |
| IGF1R | -0.455935242 | 1.75E-58 |
| UBE2Q1 | 0.383092893 | 2.14E-40 |
| ZNF37BP | -0.328950522 | 1.27E-29 |
| MSI2 | -0.369793523 | 0 |
| EFHC1 | -0.34928512 | 2.00E-33 |
| ENO1 | 0.333407307 | 0 |
| MAB21L4 | 0.337810031 | 3.04E-31 |
| NCCRP1 | 0.376998697 | 4.46E-39 |
| PIK3C3 | -0.341937924 | 5.11E-32 |
| HIPK3 | -0.304633348 | 1.94E-25 |
| AMFR | -0.309368448 | 0 |
| EGFL6 | 0.306494539 | 9.59E-26 |
| NBPF3 | -0.310666511 | 1.93E-26 |
| ART3 | 0.311510088 | 1.39E-26 |
| S100A16 | 0.382303514 | 0 |
| ZDHHC2 | -0.303861301 | 2.60E-25 |
| ANKRD29 | -0.360396801 | 1.27E-35 |
| TBC1D15 | -0.341989879 | 5.00E-32 |
| EDN2 | 0.316669074 | 1.85E-27 |
| DACH1 | -0.385718677 | 5.66E-41 |
| ZKSCAN2 | -0.337326792 | 3.74E-31 |
| LRP2BP | -0.344620254 | 1.58E-32 |
| LCORL | -0.373646592 | 2.31E-38 |
| GPATCH2L | -0.326554748 | 3.42E-29 |
| AC097534.1 | -0.348431343 | 2.93E-33 |
| ATP6V0A1 | -0.304019656 | 0 |
| RERG | -0.435893019 | 0 |
| NCR3LG1 | -0.419001232 | 9.34E-49 |
| TXNDC16 | -0.362005007 | 5.98E-36 |
| PSMB2 | 0.337373447 | 3.66E-31 |
| AC109454.2 | -0.404544881 | 2.88E-45 |
| MADD | -0.374468049 | 0 |
| AC012467.2 | -0.382520627 | 2.85E-40 |
| EGLN2 | -0.385399198 | 6.66E-41 |
| TTBK2 | -0.346295536 | 7.57E-33 |
| RASEF | -0.367549904 | 0 |
| ZNF680 | -0.334001077 | 1.54E-30 |
| LAMTOR2 | 0.358324655 | 0 |
| GLYATL2 | 0.329853111 | 8.75E-30 |
| FLNB-AS1 | -0.370104523 | 1.28E-37 |
| ZBTB4 | -0.332417391 | 2.99E-30 |
| NISCH | -0.320101542 | 4.70E-28 |
| GOLGB1 | -0.30738177 | 0 |
| SLC46A1 | -0.300076875 | 1.07E-24 |
| TRIM4 | -0.302730705 | 3.98E-25 |
| AC139769.1 | -0.317982031 | 1.10E-27 |
| LCOR | -0.337467604 | 3.52E-31 |
| SPHK1 | 0.337948831 | 2.86E-31 |
| FAR1 | -0.342927255 | 0 |
| ZNF181 | -0.324773954 | 7.10E-29 |
| CRADD | -0.307229188 | 7.25E-26 |
| CADM2 | -0.372985303 | 3.18E-38 |
| HERC2 | -0.369669428 | 1.58E-37 |
| LRP2 | -0.371265497 | 7.32E-38 |
| RCN1 | 0.312674332 | 0 |
| B3GNT3 | 0.326858652 | 3.02E-29 |
| NIPAL3 | -0.341948998 | 5.09E-32 |
| ZNF396 | -0.420334974 | 4.36E-49 |
| TMEM9B-AS1 | -0.414447392 | 1.22E-47 |
| MBLAC2 | -0.428842818 | 3.13E-51 |
| ZNF493 | -0.381667478 | 4.38E-40 |
| AC000123.3 | -0.306509843 | 9.53E-26 |
| SLC50A1 | 0.315627733 | 0 |
| AC005670.3 | -0.420484491 | 4.00E-49 |
| PHGR1 | -0.363299572 | 3.26E-36 |
| BBS4 | -0.352616984 | 4.48E-34 |
| AQP11 | -0.345916694 | 8.95E-33 |
| ZNF24 | -0.416987626 | 0 |
| AC015871.3 | -0.350236504 | 1.31E-33 |
| ITPR1-DT | -0.300618168 | 8.76E-25 |
| GPATCH8 | -0.310190959 | 2.32E-26 |
| F11R | 0.511891326 | 0 |
| PRTG | -0.32760755 | 2.22E-29 |
| TRH | -0.370306656 | 1.16E-37 |
| ZFAND4 | -0.337398536 | 3.62E-31 |
| TIMM23 | 0.302798103 | 3.88E-25 |
| RRAS | 0.317621415 | 1.26E-27 |
| AL138724.1 | 0.3535167 | 2.98E-34 |
| AL445187.1 | -0.303841537 | 2.62E-25 |
| ZKSCAN1 | -0.30784542 | 5.73E-26 |
| CYB5D2 | -0.318239566 | 0 |
| ZNF721 | -0.347309142 | 4.83E-33 |
| CYTOR | 0.349343929 | 1.95E-33 |
| MYB-AS1 | -0.341925253 | 5.14E-32 |
| RABEP1 | -0.389405462 | 8.59E-42 |
| ATP1A1-AS1 | -0.309457428 | 3.08E-26 |
| RASAL1 | 0.359379635 | 2.03E-35 |
| ZNF587B | -0.348000656 | 3.55E-33 |
| GFUS | 0.32447996 | 8.00E-29 |
| PCLO | -0.319827214 | 5.25E-28 |
| UVSSA | -0.39356336 | 9.95E-43 |
| FAM234B | -0.363948945 | 2.40E-36 |
| MIER3 | -0.312945883 | 7.97E-27 |
| AL031282.1 | -0.355379843 | 1.28E-34 |
| SECISBP2 | -0.329765521 | 9.07E-30 |
| TTC5 | -0.301878335 | 5.47E-25 |
| USP30 | -0.359212686 | 2.19E-35 |
| AC092053.4 | -0.327549479 | 2.27E-29 |
| AQP9 | 0.306682078 | 8.93E-26 |
| FSIP1 | -0.335943648 | 6.74E-31 |
| JTB | 0.402960052 | 6.79E-45 |
| EEF1AKMT4 | 0.439664772 | 0 |
| AC093827.4 | -0.326653544 | 3.29E-29 |
| SPATA46 | -0.320526813 | 3.96E-28 |
| AC007637.1 | -0.408971264 | 2.57E-46 |
| AC012313.6 | -0.340039913 | 1.16E-31 |
| LIN7A | -0.325949109 | 4.39E-29 |
| AC106738.2 | -0.383453723 | 1.78E-40 |
| GASK1B | -0.312683615 | 8.83E-27 |
| PDK2 | -0.314501568 | 4.34E-27 |
| FAN1 | -0.401366365 | 1.60E-44 |
| STRADA | -0.300078824 | 1.07E-24 |
| ZDHHC17 | -0.372745581 | 3.57E-38 |
| TMEM158 | 0.423718482 | 0 |
| JADE2 | -0.368491729 | 2.78E-37 |
| AL353804.1 | -0.347647905 | 4.16E-33 |
| KCNJ3 | -0.372741296 | 3.58E-38 |
| FUT3 | 0.470126064 | 1.50E-62 |
| TADA2B | -0.339631571 | 1.39E-31 |
| STRN3 | -0.324011429 | 9.68E-29 |
| TLCD3B | -0.329921756 | 8.50E-30 |
| TRIM52 | -0.331267363 | 4.85E-30 |
| CIPC | -0.306083878 | 0 |
| IL20RB | 0.335097178 | 9.66E-31 |
| AC008014.1 | -0.343982808 | 2.09E-32 |
| MAST4 | -0.350244912 | 1.30E-33 |
| VASN | 0.304702217 | 0 |
| AC008770.3 | -0.401765179 | 1.29E-44 |
| AC118658.1 | -0.333394278 | 1.99E-30 |
| RHOV | 0.353536853 | 0 |
| TSPYL1 | -0.32588661 | 4.50E-29 |
| TMEM40 | 0.343251564 | 2.88E-32 |
| NAF1 | -0.304833044 | 1.80E-25 |
| FBXO38 | -0.305884936 | 0 |
| CRTC2 | 0.308109144 | 5.18E-26 |
| BORCS7 | -0.315419639 | 3.02E-27 |
| FAM89B | 0.389959237 | 6.46E-42 |
| RN7SKP150 | -0.306498634 | 9.57E-26 |
| SEC22C | -0.330122051 | 7.82E-30 |
| DTX2 | 0.368634095 | 2.60E-37 |
| TTC39C | -0.371765411 | 5.75E-38 |
| WDFY3-AS2 | -0.410785967 | 9.42E-47 |
| MFSD8 | -0.330475838 | 6.75E-30 |
| SEZ6L | -0.359020094 | 2.40E-35 |
| AC016705.2 | -0.308727367 | 4.08E-26 |
| AC109583.3 | -0.392893481 | 1.41E-42 |
| RAMP2-AS1 | -0.337185911 | 3.97E-31 |
| CKS1B | 0.455777364 | 0 |
| AC093838.1 | -0.352289404 | 5.20E-34 |
| IQSEC1 | -0.336695326 | 4.90E-31 |
| FAM13B | -0.371352707 | 7.02E-38 |
| RFLNA | 0.32422752 | 8.87E-29 |
| CLPSL1 | 0.334298753 | 1.36E-30 |
| ZNF445 | -0.368466954 | 2.81E-37 |
| MCM3AP | -0.325949207 | 4.39E-29 |
| ZNF621 | -0.325466047 | 0 |
| IKZF4 | -0.315010076 | 3.55E-27 |
| AP001434.1 | 0.300644917 | 8.67E-25 |
| HAUS8 | 0.361342421 | 8.15E-36 |
| CDYL2 | -0.376383258 | 6.04E-39 |
| FBLIM1 | 0.351964113 | 6.02E-34 |
| SBSN | 0.350901704 | 9.71E-34 |
| GTF3C6 | 0.358938973 | 2.49E-35 |
| TMEM145 | -0.316609449 | 1.89E-27 |
| SAP30L | -0.304764379 | 1.85E-25 |
| CBX7 | -0.373026266 | 3.12E-38 |
| CALCOCO1 | -0.328959817 | 1.27E-29 |
| UBN2 | -0.3192822 | 6.52E-28 |
| SEM1 | 0.476711849 | 1.67E-64 |
| PFN1 | 0.475935901 | 0 |
| AC098679.5 | -0.339777968 | 1.30E-31 |
| AATK | -0.316424368 | 2.03E-27 |
| ZNF175 | -0.325292049 | 5.74E-29 |
| UBE2T | 0.348025856 | 0 |
| AL161908.1 | -0.309088031 | 3.55E-26 |
| SAMD1 | 0.327734139 | 0 |
| BRWD1 | -0.31203139 | 1.14E-26 |
| CDK2AP1 | 0.383006324 | 0 |
| TMEM144 | -0.34558027 | 1.04E-32 |
| NUP93 | 0.301243447 | 6.94E-25 |
| MOSMO | -0.324455085 | 8.08E-29 |
| TMEM161B | -0.411322495 | 0 |
| SSBP1 | 0.379199438 | 1.50E-39 |
| RAD51-AS1 | -0.307750165 | 5.94E-26 |
| ADM | 0.325487465 | 5.30E-29 |
| ELP2 | -0.400935626 | 0 |
| BRAF | -0.394902878 | 4.94E-43 |
| AC090198.1 | -0.301410358 | 6.52E-25 |
| CST9 | -0.451003534 | 4.10E-57 |
| Z95115.1 | -0.336989861 | 4.32E-31 |
| CCDC148 | -0.316774091 | 1.77E-27 |
| PMF1 | 0.372404494 | 4.22E-38 |
| FITM2 | -0.331458474 | 4.48E-30 |
| TP53TG5 | -0.460713802 | 7.86E-60 |
| NTAN1 | 0.311397554 | 1.46E-26 |
| TMSB10P1 | 0.30214192 | 4.96E-25 |
| CHAC1 | 0.315367207 | 3.09E-27 |
| ARPC5 | 0.399240543 | 4.98E-44 |
| LMBRD2 | -0.374654311 | 1.41E-38 |
| MIR573 | -0.370337644 | 1.15E-37 |
| ALG3 | 0.367085117 | 0 |
| MIR3936HG | -0.414842689 | 9.81E-48 |
| CCDC7 | -0.307066292 | 7.71E-26 |
| FH | 0.345000087 | 1.34E-32 |
| AC007878.1 | -0.333016337 | 2.33E-30 |
| AC026801.2 | -0.323125029 | 1.39E-28 |
| CCL26 | 0.32045843 | 4.07E-28 |
| AC107884.2 | -0.300982623 | 7.65E-25 |
| RHBDL2 | 0.384936812 | 8.42E-41 |
| CDC14A | -0.344929557 | 1.38E-32 |
| SERPINI1 | -0.309046503 | 3.61E-26 |
| SERINC1 | -0.337837276 | 0 |
| CMC2 | 0.344441231 | 1.71E-32 |
| COP1 | 0.342496973 | 4.01E-32 |
| AL356019.2 | -0.356438065 | 7.87E-35 |
| ZNF417 | -0.335115036 | 9.59E-31 |
| ZNF770 | -0.315251057 | 3.23E-27 |
| WFDC21P | 0.307048743 | 7.76E-26 |
| GABPB1-IT1 | -0.378604087 | 2.02E-39 |
| AC024075.1 | -0.406462209 | 0 |
| SMG6 | -0.327462917 | 2.35E-29 |
| BICRAL | -0.309567575 | 2.96E-26 |
| SLC16A6 | -0.341084037 | 7.41E-32 |
| PTK7 | 0.345036285 | 0 |
| EXOC7 | -0.330879571 | 5.70E-30 |
| UGDH-AS1 | -0.440647616 | 2.61E-54 |
| LINC00504 | -0.353221708 | 3.41E-34 |
| CDON | -0.349535273 | 1.79E-33 |
| FRG1HP | -0.309463053 | 3.08E-26 |
| NUDT16P1 | -0.315164614 | 3.34E-27 |
| CCL7 | 0.359166151 | 2.24E-35 |
| HPS4 | -0.317761771 | 1.20E-27 |
| SLC27A2 | -0.346080262 | 8.32E-33 |
| AC012513.3 | -0.352596484 | 4.52E-34 |
| PIGH | -0.305309155 | 1.50E-25 |
| ZNF33B | -0.314836595 | 0 |
| AC061961.1 | -0.376952863 | 4.56E-39 |
| SH2D2A | 0.331195744 | 5.00E-30 |
| MIGA1 | -0.407350104 | 6.25E-46 |
| LMNA | 0.328898275 | 0 |
| UPK2 | 0.342712983 | 3.65E-32 |
| FRS2 | -0.336034967 | 0 |
| CNOT6L | -0.363469996 | 0 |
| SLC15A1 | 0.340435517 | 9.81E-32 |
| SCML2P2 | -0.301434266 | 6.46E-25 |
| APH1B | -0.39476327 | 0 |
| CST9L | -0.37487359 | 1.27E-38 |
| ABHD2 | -0.35920255 | 0 |
| GAL | 0.37642633 | 5.91E-39 |
| METTL14 | -0.305705163 | 1.29E-25 |
| GUSBP9 | -0.33965608 | 1.37E-31 |
| NHLRC2 | -0.354434077 | 1.97E-34 |
| SRM | 0.33968454 | 0 |
| MAGOH | 0.353903098 | 2.50E-34 |
| MICOS10 | -0.356680226 | 7.04E-35 |
| GRAMD4P8 | -0.340882894 | 8.09E-32 |
| KCND3 | -0.353850485 | 2.56E-34 |
| AC060780.2 | -0.300221063 | 1.02E-24 |
| RPS18P9 | -0.333428056 | 1.96E-30 |
| DNAJC27 | -0.340792241 | 8.41E-32 |
| S100A7A | 0.318467856 | 9.03E-28 |
| MRPS30 | -0.346956263 | 5.65E-33 |
| IL6ST | -0.33996987 | 0 |
| PACRGL | -0.331588741 | 4.24E-30 |
| AL139396.1 | -0.313012059 | 7.77E-27 |
| OAZ3 | 0.324137558 | 9.20E-29 |
| ADD1 | -0.306880495 | 0 |
| METTL15 | -0.318531998 | 8.80E-28 |
| SH3BP1 | 0.358289658 | 3.36E-35 |
| HEXIM2 | -0.388015747 | 1.75E-41 |
| CLCN6 | -0.308217281 | 4.97E-26 |
| AC008663.2 | -0.317474602 | 1.34E-27 |
| RERG-IT1 | -0.471435585 | 6.19E-63 |
| ZXDA | -0.322809253 | 1.58E-28 |
| ARID4A | -0.331150195 | 5.09E-30 |
| CKMT2-AS1 | -0.31244304 | 9.70E-27 |
| LINC01863 | -0.328198223 | 1.74E-29 |
| LSM2 | 0.371714749 | 0 |
| APPBP2 | -0.321405269 | 2.78E-28 |
| UBR1 | -0.300651966 | 8.65E-25 |
| APBB2 | -0.359729733 | 0 |
| CDC42EP1 | 0.342700817 | 0 |
| COX10-AS1 | -0.315977311 | 2.43E-27 |
| FXYD5 | 0.38894025 | 1.09E-41 |
| AC135050.5 | -0.308048405 | 5.30E-26 |
| FHL3 | 0.366069595 | 8.81E-37 |
| SNRPG | 0.477787553 | 0 |
| AC006130.3 | -0.331639048 | 4.15E-30 |
| RPL23AP64 | -0.304186229 | 2.30E-25 |
| RARRES1 | 0.342495392 | 0 |
| NR1D2 | -0.305239555 | 1.54E-25 |
| SRGAP3 | -0.313550909 | 6.30E-27 |
| PCF11 | -0.315011796 | 3.55E-27 |
| AC084198.2 | -0.308987594 | 3.69E-26 |
| NRIP2 | -0.32612557 | 4.08E-29 |
| AKAP9 | -0.307030584 | 0 |
| ZNF585B | -0.306531758 | 9.45E-26 |
| SEZ6 | -0.344192478 | 1.91E-32 |
| SNRPC | 0.352076748 | 0 |
| TAPT1 | -0.388723593 | 1.22E-41 |
| CNNM2 | -0.378123682 | 2.56E-39 |
| AL049840.2 | -0.309104208 | 3.53E-26 |
| CALML5 | 0.335925336 | 6.80E-31 |
| CKS2 | 0.300342188 | 9.70E-25 |
| DCLK1 | -0.307170121 | 7.41E-26 |
| ZNF397 | -0.341619305 | 5.87E-32 |
| DEGS1 | 0.3820762 | 0 |
| ZNF543 | -0.315791118 | 2.61E-27 |
| ICA1L | -0.408062853 | 4.23E-46 |
| EBLN3P | -0.337197033 | 0 |
| VTN | -0.341374015 | 6.53E-32 |
| AL049869.2 | -0.305213269 | 1.56E-25 |
