# Supplementary material for: Exploring the prognostic value of S100A11 and its association with immune infiltration in breast cancer
Source: Sci Rep. 2023 Dec 21;13:22922. doi: 10.1038/s41598-023-50160-x (PMC10739898; doi:10.1038/s41598-023-50160-x)
Supplement: Supplementary file 4 — Supplementary Table S3. [file 41598_2023_50160_MOESM4_ESM.docx]

**Supplementary Table S3 S100A11-related DEGs**

| **gene** | **logFC** | **pValue** | **fdr** |
| --- | --- | --- | --- |
| CLVS2 | -3.243535559 | 4.48E-08 | 1.66E-07 |
| AC024610.2 | -1.647390888 | 0.000856988 | 0.001591461 |
| AC090519.1 | -1.517535919 | 7.67E-07 | 2.33E-06 |
| RN7SL674P | -3.458421443 | 1.17E-15 | 1.57E-14 |
| NRAD1 | 2.240304212 | 1.25E-13 | 1.17E-12 |
| ITPK1-AS1 | -2.001959882 | 3.35E-07 | 1.08E-06 |
| FETUB | 1.630518607 | 6.69E-10 | 3.34E-09 |
| CHI3L2 | 1.716533413 | 1.14E-14 | 1.28E-13 |
| AC011933.2 | -2.112743386 | 0.000288091 | 0.000580727 |
| AC012613.2 | -2.024686714 | 3.76E-15 | 4.57E-14 |
| AC104454.2 | 2.304709556 | 2.03E-20 | 7.15E-19 |
| KRT24 | -1.572615124 | 2.02E-05 | 4.92E-05 |
| KRT79 | 4.892342716 | 0.00623142 | 0.009940565 |
| AC092574.2 | -1.799562413 | 1.16E-14 | 1.29E-13 |
| ANKRD30A | -1.518950302 | 6.86E-21 | 2.65E-19 |
| BMPR1B | -1.64081635 | 2.21E-11 | 1.40E-10 |
| NPY2R | -3.047453674 | 4.94E-07 | 1.55E-06 |
| XKR7 | -3.527423071 | 0.002617166 | 0.004472262 |
| THRA1/BTR | -2.061476704 | 0.003193972 | 0.00537383 |
| FAM135B | -2.54837032 | 4.90E-14 | 4.93E-13 |
| AC008663.3 | -1.572445357 | 2.23E-22 | 1.19E-20 |
| AC009560.3 | -1.530742761 | 3.60E-13 | 3.07E-12 |
| FBXO40 | -2.302243896 | 0.001008179 | 0.001850638 |
| DCDC1 | -1.800028069 | 4.85E-23 | 2.97E-21 |
| LHX1-DT | 2.155346743 | 0.000168592 | 0.000353639 |
| SLC1A6 | 2.764678699 | 2.67E-09 | 1.21E-08 |
| AC107208.1 | 2.783279423 | 0.003589636 | 0.00597919 |
| GSTA1 | 1.818646589 | 0.001841658 | 0.003231935 |
| TMPRSS6 | -1.577646811 | 6.57E-20 | 2.06E-18 |
| CYP2T3P | -1.924875161 | 8.19E-05 | 0.000180733 |
| LMOD2 | -1.527956205 | 0.001822222 | 0.003201017 |
| AC008514.1 | 1.917800604 | 5.52E-13 | 4.54E-12 |
| CNGA3 | -2.571483851 | 2.34E-13 | 2.06E-12 |
| AC078993.1 | -1.636787142 | 7.19E-20 | 2.23E-18 |
| AL158212.4 | -1.766902257 | 3.80E-09 | 1.68E-08 |
| AC108925.1 | 2.133783855 | 1.67E-10 | 9.20E-10 |
| CWH43 | 2.018656251 | 5.66E-17 | 9.39E-16 |
| MDGA2 | 1.566918657 | 4.09E-09 | 1.79E-08 |
| SERPINA9 | -1.51257983 | 7.79E-16 | 1.08E-14 |
| AC098679.4 | -1.596886145 | 4.16E-23 | 2.60E-21 |
| AC005544.1 | -1.908187618 | 1.56E-22 | 8.68E-21 |
| GABRA5 | 3.119244437 | 1.61E-06 | 4.64E-06 |
| LINC02224 | -1.637553981 | 6.61E-20 | 2.07E-18 |
| AL356311.1 | -1.878985042 | 2.21E-23 | 1.48E-21 |
| KRT6B | 2.469304636 | 2.13E-10 | 1.15E-09 |
| SLC26A9 | 1.978054909 | 1.61E-15 | 2.09E-14 |
| TAS2R3 | -1.993340829 | 2.67E-14 | 2.80E-13 |
| AC096733.2 | -1.895463373 | 3.54E-42 | 1.66E-38 |
| KRT16P2 | 2.02501231 | 3.12E-09 | 1.40E-08 |
| AL512363.1 | 1.593028123 | 7.62E-07 | 2.32E-06 |
| CXCL6 | 1.80655944 | 5.37E-05 | 0.000122276 |
| AC012513.2 | -2.167651722 | 0.003560518 | 0.005935234 |
| AP002358.2 | 1.57952386 | 2.25E-05 | 5.45E-05 |
| CLLU1-AS1 | 1.609263943 | 7.99E-10 | 3.93E-09 |
| CCL20 | 1.59197476 | 7.68E-26 | 9.48E-24 |
| LEMD1 | 2.320637858 | 7.03E-11 | 4.11E-10 |
| LINC02880 | -1.74213364 | 1.72E-08 | 6.83E-08 |
| BLACAT1 | 1.733040863 | 1.55E-10 | 8.57E-10 |
| UNC13A | -1.976795061 | 0.020990273 | 0.030295628 |
| MAGEA8 | -2.136248695 | 4.56E-05 | 0.000105011 |
| GDPD4 | -1.973308068 | 0.001603987 | 0.002847658 |
| SLIT3-AS1 | -2.052827576 | 0.014691293 | 0.021815493 |
| UNC5D | -3.206010412 | 0.00232844 | 0.004011974 |
| AC018714.2 | 1.941288633 | 5.85E-11 | 3.46E-10 |
| AC104984.4 | -1.653337905 | 0.000336599 | 0.00067084 |
| CRABP1 | 1.8985713 | 8.65E-16 | 1.18E-14 |
| SULT1C2P1 | -1.580514281 | 0.000760481 | 0.001424165 |
| FGF10-AS1 | -2.063877898 | 2.39E-08 | 9.27E-08 |
| IL36G | 1.783292497 | 2.50E-06 | 7.00E-06 |
| SLC2A14 | 1.628682488 | 1.59E-14 | 1.74E-13 |
| AL138773.1 | -1.666245341 | 0.000319818 | 0.000640204 |
| BRDT | -1.61406829 | 3.23E-09 | 1.44E-08 |
| CSAG3 | 1.600840187 | 0.000163225 | 0.000343172 |
| SLITRK6 | -1.662171302 | 1.74E-09 | 8.08E-09 |
| AC079866.1 | -2.797995287 | 3.10E-12 | 2.24E-11 |
| SLIT3-AS2 | -1.604765962 | 4.62E-23 | 2.86E-21 |
| ADCY1 | -1.807874863 | 3.37E-18 | 7.33E-17 |
| AL162578.1 | -2.543922499 | 0.021289822 | 0.03069338 |
| DUSP5P2 | 1.68017773 | 1.84E-21 | 8.05E-20 |
| MMP20 | 1.89058874 | 3.36E-05 | 7.91E-05 |
| C4orf54 | 2.094906448 | 1.33E-15 | 1.75E-14 |
| ROPN1 | 1.787655334 | 1.37E-09 | 6.45E-09 |
| AC096589.1 | -1.680787682 | 2.87E-10 | 1.52E-09 |
| NXPH3 | -1.575512612 | 6.26E-33 | 4.52E-30 |
| NDUFB4P11 | 1.631402484 | 3.87E-10 | 2.00E-09 |
| AC005550.2 | -1.845243841 | 0.000368866 | 0.000730499 |
| CXCL1 | 2.150367357 | 3.29E-12 | 2.37E-11 |
| SLC39A12 | 1.770355282 | 3.89E-07 | 1.24E-06 |
| MAG | -1.597663791 | 2.68E-22 | 1.40E-20 |
| KCNC2 | -2.997740464 | 6.88E-17 | 1.13E-15 |
| AF178030.1 | -2.352610514 | 4.85E-09 | 2.10E-08 |
| SLPI | 1.563689466 | 1.67E-23 | 1.16E-21 |
| FAF1-AS1 | -1.598816986 | 0.00344663 | 0.005765186 |
| POU5F2 | -1.895530415 | 0.014993271 | 0.022218241 |
| KISS1 | 1.637870935 | 9.48E-11 | 5.43E-10 |
| AC100826.1 | -2.05136128 | 1.05E-10 | 5.99E-10 |
| AC011755.1 | -1.746689913 | 0.003019406 | 0.005103285 |
| OLFM4 | 1.531161141 | 0.002212234 | 0.003829546 |
| RPS16P5 | -2.205543615 | 1.63E-05 | 4.03E-05 |
| AC023403.1 | 1.649954479 | 4.84E-05 | 0.00011105 |
| TMEM171 | 1.506057349 | 2.99E-25 | 3.20E-23 |
| AL109807.1 | -1.783390584 | 0.017093033 | 0.025077911 |
| AC016705.3 | -2.917466704 | 8.64E-09 | 3.60E-08 |
| AC108690.1 | -2.841008153 | 0.000152636 | 0.00032221 |
| AL357568.1 | -1.823264482 | 4.75E-07 | 1.49E-06 |
| LINC01133 | 2.237100862 | 6.23E-23 | 3.74E-21 |
| SHANK2-AS2 | -2.112650196 | 6.74E-05 | 0.000150861 |
| CDH10 | -2.388638535 | 0.000412328 | 0.000809401 |
| NMU | 1.728994671 | 1.41E-13 | 1.30E-12 |
| GABRA2 | -1.565174523 | 4.78E-11 | 2.87E-10 |
| PKP1 | 1.561817191 | 9.08E-10 | 4.43E-09 |
| TBC1D9 | -1.605491724 | 2.45E-39 | 5.75E-36 |
| LINC02437 | 1.674400756 | 3.95E-13 | 3.34E-12 |
| AC008679.1 | -1.530698491 | 4.92E-08 | 1.81E-07 |
| CLDN9 | 1.643356017 | 1.51E-14 | 1.66E-13 |
| AL035427.2 | -1.531376551 | 0.000265475 | 0.000538539 |
| ROPN1B | 1.61913378 | 2.96E-09 | 1.33E-08 |
| AC004947.1 | -2.934645917 | 4.66E-11 | 2.80E-10 |
| DSCAML1 | -1.500999974 | 6.32E-12 | 4.37E-11 |
| CHRNB2 | -1.734862737 | 7.10E-06 | 1.85E-05 |
| AL022724.2 | 1.535096641 | 1.52E-11 | 9.87E-11 |
| AC025175.2 | -1.600362032 | 4.50E-08 | 1.67E-07 |
| XIAPP2 | -1.615771998 | 0.00502809 | 0.00815018 |
| IL1RAPL1 | -1.685329222 | 4.74E-17 | 8.00E-16 |
| MKX | -2.405697449 | 2.05E-06 | 5.82E-06 |
| ITGB5-AS1 | -1.743675633 | 5.03E-17 | 8.44E-16 |
| AL031668.2 | 1.837765102 | 0.001944501 | 0.00339718 |
| LINC01983 | -2.200005675 | 3.79E-17 | 6.53E-16 |
| AL731533.3 | 2.106116883 | 4.39E-09 | 1.92E-08 |
| RN7SL827P | -4.024104796 | 1.69E-07 | 5.71E-07 |
| RAB39B | -1.530686604 | 6.74E-15 | 7.86E-14 |
| PICSAR | 1.594723508 | 4.65E-11 | 2.80E-10 |
| C7 | -1.520388175 | 6.53E-12 | 4.51E-11 |
| DPY19L2P4 | -2.095868046 | 3.14E-21 | 1.30E-19 |
| LINC00705 | 2.45706185 | 1.91E-10 | 1.04E-09 |
| AC009102.2 | -1.762399969 | 3.26E-17 | 5.69E-16 |
| ERICH3 | -1.833195919 | 2.35E-13 | 2.08E-12 |
| SLC30A8 | -1.736358581 | 1.19E-06 | 3.50E-06 |
| CASP14 | 2.531560681 | 3.46E-15 | 4.25E-14 |
| AC079296.2 | -2.475376778 | 1.12E-17 | 2.16E-16 |
| PTPRN2 | -1.657059789 | 2.46E-18 | 5.49E-17 |
| CST2 | -1.862217339 | 2.02E-14 | 2.16E-13 |
| DEFB1 | 2.449896902 | 1.62E-18 | 3.72E-17 |
| AC008674.1 | -1.648313968 | 6.96E-16 | 9.68E-15 |
| AC022726.2 | -2.844754066 | 1.07E-14 | 1.20E-13 |
| NGF-AS1 | -1.717162092 | 0.000487248 | 0.000943455 |
| AC005183.1 | -1.8121531 | 0.000899491 | 0.001663374 |
| LINC01879 | -1.920284802 | 8.42E-21 | 3.19E-19 |
| AC012370.1 | -1.793857061 | 3.35E-11 | 2.07E-10 |
| AC023481.1 | -2.011927314 | 0.00016177 | 0.000340292 |
| S100A8 | 3.170062597 | 2.88E-28 | 6.81E-26 |
| LINC02251 | 1.633984313 | 0.00058572 | 0.001117763 |
| SLC7A3 | -1.791344497 | 1.16E-11 | 7.69E-11 |
| IVL | 2.526360901 | 1.19E-18 | 2.79E-17 |
| POU4F3 | -1.524752252 | 8.24E-05 | 0.0001816 |
| AC010547.2 | 1.502805193 | 1.58E-07 | 5.37E-07 |
| AC092118.1 | 1.782433081 | 1.67E-13 | 1.52E-12 |
| STARD13-AS | -2.298086364 | 0.000327836 | 0.000654766 |
| AL021068.1 | -1.616074048 | 3.24E-24 | 2.62E-22 |
| AC007431.1 | -1.855219738 | 6.49E-05 | 0.000145574 |
| KRT9 | 1.987065497 | 8.63E-10 | 4.23E-09 |
| GFRA3 | 1.671594888 | 1.41E-11 | 9.23E-11 |
| SYN3-AS1 | -2.093561242 | 5.85E-21 | 2.29E-19 |
| THSD4 | -1.745069156 | 7.28E-39 | 1.58E-35 |
| SCARNA6 | -3.520740878 | 2.25E-10 | 1.21E-09 |
| AFAP1-AS1 | 1.667610793 | 1.99E-05 | 4.84E-05 |
| LINC02115 | -3.633821559 | 1.55E-14 | 1.69E-13 |
| CD5L | 2.053045473 | 0.009162964 | 0.01416707 |
| C1orf105 | 3.593539472 | 2.90E-13 | 2.51E-12 |
| LINC01198 | 1.63374704 | 4.19E-05 | 9.71E-05 |
| AC133785.1 | 1.665062478 | 5.07E-11 | 3.03E-10 |
| TEX101 | -1.829149409 | 1.57E-09 | 7.36E-09 |
| LINC01170 | 1.579349592 | 0.004964467 | 0.008055394 |
| NOVA1 | -2.436708743 | 8.88E-33 | 6.26E-30 |
| AC099535.2 | -1.762472596 | 6.64E-09 | 2.81E-08 |
| PDSS1P1 | -1.565608184 | 0.000107529 | 0.000232815 |
| AL360169.3 | -2.38772539 | 0.002816206 | 0.004787704 |
| RNU6-813P | -1.613402536 | 4.75E-15 | 5.68E-14 |
| A2ML1 | 2.977048836 | 2.88E-21 | 1.21E-19 |
| AC007496.3 | -1.653161035 | 0.002090209 | 0.003632372 |
| KRT16 | 2.573882484 | 8.71E-22 | 4.06E-20 |
| AC021517.3 | -1.653661313 | 5.84E-17 | 9.64E-16 |
| AP003059.1 | -2.158774967 | 7.07E-16 | 9.82E-15 |
| AC020907.1 | 1.517217736 | 8.84E-17 | 1.42E-15 |
| PPP1R14C | 2.323653666 | 1.25E-21 | 5.67E-20 |
| NELL1 | -2.44303911 | 2.01E-07 | 6.69E-07 |
| AC012588.1 | -1.789842292 | 6.14E-16 | 8.65E-15 |
| DDX3P1 | -2.135552736 | 0.002052338 | 0.00357273 |
| ASIC2 | -1.543536227 | 1.23E-07 | 4.24E-07 |
| AC103740.2 | 1.866852284 | 3.95E-18 | 8.48E-17 |
| AC064799.2 | -3.421734544 | 1.22E-27 | 2.34E-25 |
| VGLL1 | 2.447810383 | 1.80E-23 | 1.24E-21 |
| HTR1E | -1.640441802 | 2.23E-10 | 1.20E-09 |
| AL445487.1 | 1.641918684 | 0.00073717 | 0.001383378 |
| KLK2 | -2.108488593 | 0.003787324 | 0.006277301 |
| LHX1 | 2.354711165 | 0.000181317 | 0.000378191 |
| WARS2-IT1 | -1.79795377 | 1.36E-13 | 1.26E-12 |
| RN7SL559P | -3.78587486 | 2.32E-05 | 5.60E-05 |
| CLSTN2 | -2.360624795 | 2.46E-37 | 4.33E-34 |
| AL023693.1 | -1.709758683 | 2.19E-10 | 1.18E-09 |
| AC009716.2 | -2.901354626 | 2.28E-11 | 1.44E-10 |
| CALB2 | 2.036979818 | 1.85E-10 | 1.01E-09 |
| CALCA | 1.662088553 | 0.014944377 | 0.022157356 |
| SNORA79B | -2.340775955 | 6.05E-05 | 0.000136365 |
| WDR17 | -1.671676193 | 7.86E-17 | 1.27E-15 |
| AC093297.2 | -1.614474151 | 3.89E-24 | 3.11E-22 |
| AL158828.1 | 1.805371861 | 2.42E-05 | 5.81E-05 |
| SRARP | -1.510167413 | 6.39E-29 | 1.92E-26 |
| ACADSB | -1.564287199 | 1.50E-40 | 4.23E-37 |
| RGS20 | 1.98725878 | 1.95E-12 | 1.46E-11 |
| RP1 | -1.789332426 | 5.39E-19 | 1.37E-17 |
| AC120498.10 | -1.627517598 | 3.94E-17 | 6.77E-16 |
| AC007494.2 | -1.700105924 | 0.010334233 | 0.015818276 |
| MAGEB4 | 2.552694448 | 5.69E-10 | 2.87E-09 |
| RLN2 | -1.611511246 | 2.32E-12 | 1.72E-11 |
| SCGN | -1.977373858 | 0.000360359 | 0.000715159 |
| HRCT1 | 1.65128511 | 1.40E-07 | 4.78E-07 |
| AC009560.4 | -1.768774254 | 9.67E-14 | 9.23E-13 |
| AC007496.1 | -1.781989284 | 0.000412986 | 0.000810527 |
| MFSD1P1 | -2.274681384 | 0.000147304 | 0.000311795 |
| TEX14 | -2.125701463 | 4.23E-15 | 5.10E-14 |
| GHRH | -1.517605761 | 4.09E-09 | 1.79E-08 |
| AL445933.2 | -1.538356838 | 1.18E-16 | 1.87E-15 |
| TPH2 | -1.743310722 | 0.008748436 | 0.013570094 |
| AC021517.1 | -1.680956265 | 1.38E-14 | 1.52E-13 |
| AC013652.2 | -2.514097407 | 5.33E-15 | 6.32E-14 |
| IRAIN | -1.732636664 | 1.50E-11 | 9.75E-11 |
| AC012404.2 | -1.964219925 | 1.71E-08 | 6.77E-08 |
| LINC00113 | 1.881143707 | 2.04E-08 | 8.00E-08 |
| AL136115.1 | -1.567457134 | 1.10E-19 | 3.27E-18 |
| AL359555.1 | 1.53533523 | 7.24E-12 | 4.96E-11 |
| AL138740.1 | -1.865185554 | 6.33E-13 | 5.16E-12 |
| FGF14 | -2.316966905 | 7.02E-12 | 4.82E-11 |
| BCAR4 | 2.326954482 | 0.002154174 | 0.003736386 |
| TAS2R30 | -2.501963146 | 0.000136503 | 0.000290263 |
| ACAN | 2.90317277 | 0.000361012 | 0.000716355 |
| LINC01856 | -2.316860969 | 1.57E-16 | 2.42E-15 |
| HSD3B2 | -5.717123386 | 5.34E-07 | 1.66E-06 |
| HEPHL1 | 2.033132346 | 2.77E-09 | 1.25E-08 |
| CYP17A1 | -2.062098612 | 1.08E-07 | 3.75E-07 |
| SLC14A2 | -1.964823564 | 2.22E-18 | 4.99E-17 |
| ASB15 | -1.620053449 | 3.77E-17 | 6.52E-16 |
| AC010476.2 | -1.641174777 | 7.25E-14 | 7.07E-13 |
| DLX2 | -1.732227764 | 1.17E-10 | 6.58E-10 |
| CADPS | -1.981001596 | 1.29E-06 | 3.78E-06 |
| AC087286.4 | -1.725202447 | 3.32E-05 | 7.80E-05 |
| MYOC | -3.078056702 | 1.20E-05 | 3.02E-05 |
| FOXP1-IT1 | -2.323663381 | 2.75E-13 | 2.40E-12 |
| CA6 | 2.01279782 | 0.015392952 | 0.022759048 |
| SPATA31C2 | -1.892387578 | 7.30E-09 | 3.07E-08 |
| TATDN2P3 | -2.313275322 | 2.13E-06 | 6.04E-06 |
| SYNPO2L | -2.901401025 | 3.39E-14 | 3.50E-13 |
| GS1-594A7.3 | 1.928072333 | 2.86E-33 | 2.30E-30 |
| RNF222 | 1.882516272 | 4.16E-07 | 1.32E-06 |
| FBN3 | 1.557248776 | 5.29E-07 | 1.65E-06 |
| SNORA47 | -1.748264603 | 0.003796319 | 0.00628999 |
| VN1R53P | -1.673305045 | 6.19E-21 | 2.41E-19 |
| ORM2 | 1.575427134 | 1.25E-15 | 1.65E-14 |
| SERPINB2 | 1.57537761 | 0.002630002 | 0.004492289 |
| RUNDC3A | -2.516529003 | 0.015344286 | 0.022695431 |
| RD3 | -1.624036695 | 1.33E-14 | 1.47E-13 |
| AC114811.2 | -1.640869345 | 2.36E-05 | 5.70E-05 |
| AC011944.2 | -1.773330874 | 6.56E-09 | 2.78E-08 |
| AC092667.1 | -1.839022856 | 1.20E-33 | 1.20E-30 |
| LINC02668 | 2.653960886 | 0.000648602 | 0.001227494 |
| AL049767.1 | 2.327156924 | 7.56E-13 | 6.07E-12 |
| ERN2 | 1.863155806 | 0.001778598 | 0.003130239 |
| AC108057.1 | -2.831813482 | 7.90E-16 | 1.09E-14 |
| UHRF2P1 | -3.234246445 | 0.005276077 | 0.008518823 |
| TMEM26 | -1.87535051 | 1.93E-24 | 1.68E-22 |
| SNORA74B | -5.397647679 | 0.000997804 | 0.001832549 |
| AP000821.1 | -2.156840857 | 3.47E-22 | 1.77E-20 |
| OSBPL10-AS1 | -1.867341652 | 0.001393268 | 0.002500692 |
| GRIK1 | -2.516814502 | 0.03214422 | 0.044741794 |
| RN7SL838P | -2.398751081 | 9.54E-08 | 3.35E-07 |
| CYP2G1P | -2.077861819 | 2.74E-13 | 2.39E-12 |
| MATN4 | 1.636751255 | 7.32E-08 | 2.62E-07 |
| ASCL1 | -3.678971403 | 7.48E-08 | 2.67E-07 |
| LINC00513 | -2.204788569 | 0.000375797 | 0.000742962 |
| AC006296.3 | -1.732419666 | 3.68E-17 | 6.37E-16 |
| DUSP9 | 1.691772033 | 9.11E-25 | 8.53E-23 |
| AC126564.1 | -1.562677143 | 3.94E-11 | 2.40E-10 |
| LINC01436 | 1.946921518 | 2.48E-07 | 8.16E-07 |
| TPH1 | -3.175623522 | 1.52E-05 | 3.77E-05 |
| CPA4 | 1.829668034 | 4.24E-24 | 3.35E-22 |
| ATP1A2 | -2.394239692 | 1.08E-27 | 2.14E-25 |
| AC125603.2 | -2.421799476 | 8.30E-19 | 2.01E-17 |
| PGLYRP3 | 1.58748151 | 7.32E-22 | 3.50E-20 |
| EIF4BP5 | -1.689993434 | 1.48E-06 | 4.30E-06 |
| HNRNPRP1 | -1.802250528 | 0.031181956 | 0.043523876 |
| AP000851.1 | 1.703717172 | 1.46E-05 | 3.62E-05 |
| CYP2C18 | 1.80983523 | 4.74E-08 | 1.75E-07 |
| HEPACAM2 | -2.40397868 | 8.67E-21 | 3.27E-19 |
| AC108457.1 | 2.133931623 | 3.90E-07 | 1.24E-06 |
| AL139095.3 | 2.129128419 | 4.18E-13 | 3.53E-12 |
| AC005343.4 | -1.556074016 | 0.000562869 | 0.001077805 |
| NKX2-5 | 1.640417514 | 6.78E-06 | 1.77E-05 |
| HDC | -3.842268028 | 5.43E-16 | 7.72E-15 |
| CDHR3 | -1.587067095 | 1.80E-10 | 9.89E-10 |
| CPLX2 | -5.265001721 | 0.000210916 | 0.0004358 |
| AC103705.1 | -2.0704475 | 1.50E-05 | 3.71E-05 |
| SIX3 | 1.537393037 | 1.44E-12 | 1.10E-11 |
| GACAT2 | -2.657551393 | 1.13E-12 | 8.77E-12 |
| APOB | -1.540951343 | 0.000390612 | 0.00077037 |
| PADI3 | 1.969318226 | 0.013096953 | 0.019620708 |
| NUDT19P5 | -1.803495283 | 8.52E-19 | 2.06E-17 |
| FAM240A | 2.456272453 | 2.94E-12 | 2.14E-11 |
| S100A12 | 1.613073775 | 6.35E-09 | 2.70E-08 |
| PCDH19 | -2.128735918 | 1.73E-19 | 4.98E-18 |
| AC103702.2 | 2.040264994 | 1.39E-10 | 7.76E-10 |
| P2RY4 | -2.048777609 | 3.28E-13 | 2.82E-12 |
| TNR | -1.607485342 | 4.90E-07 | 1.54E-06 |
| ATRNL1 | -1.791839284 | 5.73E-09 | 2.46E-08 |
| HSPE1P26 | -1.803626293 | 1.51E-13 | 1.39E-12 |
| LINC02250 | -1.586915738 | 0.000637829 | 0.001208773 |
| LINC02676 | -2.250115488 | 6.18E-10 | 3.10E-09 |
| AC112253.1 | -1.996326057 | 0.000114143 | 0.000245907 |
| AL033519.5 | -2.232955737 | 0.007204298 | 0.011344162 |
| AP003080.1 | -1.723299129 | 1.43E-18 | 3.30E-17 |
| NRSN1 | -3.021299445 | 0.008030126 | 0.012533867 |
| RIMS4 | -1.929016506 | 1.18E-15 | 1.58E-14 |
| KCNC1 | -2.888144408 | 7.11E-17 | 1.16E-15 |
| SRRM4 | -2.713516487 | 0.001222421 | 0.002213904 |
| FGFBP2 | 1.71739963 | 0.00159119 | 0.002827076 |
| Z98885.1 | -1.578342425 | 6.14E-08 | 2.23E-07 |
| MYOT | -1.795287302 | 2.64E-08 | 1.02E-07 |
| LINC02783 | 1.878029228 | 0.000165847 | 0.000348374 |
| NKAIN1 | -1.643195817 | 7.78E-22 | 3.68E-20 |
| CAMKV | 1.747996847 | 4.12E-12 | 2.92E-11 |
| IGF2BP2 | 1.548651768 | 2.33E-12 | 1.73E-11 |
| KRTAP2-3 | 2.332871081 | 3.51E-07 | 1.13E-06 |
| PIEZO2 | -1.596916453 | 3.97E-21 | 1.61E-19 |
| AL353803.4 | -1.745426393 | 4.78E-09 | 2.08E-08 |
| KLK3 | -2.747685582 | 2.77E-06 | 7.69E-06 |
| FGFBP1 | 2.454239553 | 5.06E-09 | 2.19E-08 |
| MYOG | -2.251618157 | 5.39E-06 | 1.43E-05 |
| AC024592.2 | 2.135877664 | 2.78E-14 | 2.91E-13 |
| C1QL2 | 1.564485718 | 3.80E-14 | 3.89E-13 |
| AGTR1 | -2.080528975 | 4.41E-24 | 3.42E-22 |
| CST5 | -5.190488753 | 5.46E-16 | 7.75E-15 |
| AC108451.1 | -1.721505488 | 4.87E-09 | 2.11E-08 |
| OPRK1 | 1.844810283 | 2.36E-13 | 2.08E-12 |
| GSDMC | 1.887269058 | 8.59E-35 | 1.15E-31 |
| CNGB1 | 1.67739521 | 8.13E-08 | 2.88E-07 |
| RNY1P14 | -2.787315847 | 5.84E-15 | 6.88E-14 |
| AC090950.1 | -1.707518049 | 4.86E-09 | 2.11E-08 |
| AL356113.1 | -1.5534213 | 0.002023068 | 0.003525479 |
| IGFN1 | -1.522914761 | 1.98E-06 | 5.63E-06 |
| TRIM15 | 1.640321481 | 1.19E-15 | 1.59E-14 |
| TPSD1 | -1.808693081 | 6.33E-08 | 2.29E-07 |
| MPZ | 2.398539041 | 0.008765394 | 0.01359341 |
| AC025271.2 | -1.503330176 | 3.85E-09 | 1.70E-08 |
| AC008277.1 | -2.273364287 | 4.51E-05 | 0.000103846 |
| GABBR2 | 1.86602073 | 1.31E-16 | 2.05E-15 |
| AP003696.1 | -1.518599396 | 0.000435921 | 0.000851561 |
| ZBTB16 | -1.901445602 | 1.11E-27 | 2.16E-25 |
| AC046158.3 | -1.578270828 | 0.000373316 | 0.000738325 |
| WFDC12 | 2.191436421 | 1.09E-06 | 3.22E-06 |
| SRMP2 | -2.373606322 | 3.05E-08 | 1.16E-07 |
| LINC01411 | -2.221831011 | 3.04E-17 | 5.38E-16 |
| TAFA3 | 1.699660373 | 4.90E-08 | 1.80E-07 |
| CSF2 | 1.565287865 | 2.68E-05 | 6.40E-05 |
| ADGRF2 | 2.152348613 | 7.38E-08 | 2.64E-07 |
| KRT78 | 2.376414978 | 4.58E-17 | 7.75E-16 |
| RTL9 | -2.217842686 | 1.07E-09 | 5.16E-09 |
| AC091305.1 | -2.413240045 | 0.000373237 | 0.000738221 |
| ABCC8 | -1.555238667 | 3.11E-26 | 4.38E-24 |
| MIR133A1HG | -2.278120274 | 1.89E-07 | 6.34E-07 |
| NDUFB4P10 | -1.638148268 | 1.73E-10 | 9.48E-10 |
| AC092979.1 | -4.564162174 | 3.14E-11 | 1.94E-10 |
| CCL25 | 1.788422134 | 0.029270547 | 0.041090532 |
| C18orf15 | -2.678811693 | 0.000215852 | 0.000445312 |
| LUZP2 | -1.517260839 | 1.25E-13 | 1.17E-12 |
| NLRP8 | -2.115251919 | 2.67E-17 | 4.79E-16 |
| FDCSP | 2.115674885 | 6.48E-06 | 1.70E-05 |
| MAPT-IT1 | -2.155200142 | 1.62E-28 | 4.00E-26 |
| PRSS35 | -1.682202399 | 7.11E-11 | 4.15E-10 |
| PPIAP26 | 1.710773407 | 0.000419077 | 0.000821677 |
| NLRP5 | -1.575686596 | 3.28E-11 | 2.02E-10 |
| PCCA-AS1 | -1.598281191 | 1.74E-10 | 9.53E-10 |
| KLHDC7B | 1.710195167 | 9.86E-09 | 4.07E-08 |
| SLC5A7 | -3.312192791 | 6.62E-06 | 1.73E-05 |
| AL096803.2 | 2.262354722 | 1.04E-06 | 3.10E-06 |
| AL139383.1 | -1.592114654 | 3.98E-20 | 1.31E-18 |
| CRYAB | 1.580884982 | 1.83E-06 | 5.23E-06 |
| AL139393.2 | 1.5147085 | 1.71E-08 | 6.79E-08 |
| SNORA38B | -1.98233847 | 0.000127815 | 0.000272776 |
| AP000331.1 | -1.594594423 | 6.27E-12 | 4.34E-11 |
| CHRNA9 | -2.527349498 | 0.000246711 | 0.000503191 |
| AC092127.2 | -1.563085499 | 1.03E-10 | 5.88E-10 |
| NLRP7 | 2.042414434 | 0.014298156 | 0.021278816 |
| OR7E13P | -1.897513655 | 6.07E-10 | 3.05E-09 |
| ELOVL2 | -1.737481489 | 1.65E-21 | 7.32E-20 |
| AC069410.1 | -1.695344524 | 1.39E-16 | 2.17E-15 |
| LINC02487 | 1.788870989 | 3.89E-07 | 1.24E-06 |
| KRT75 | 1.940801507 | 8.05E-10 | 3.96E-09 |
| ROS1 | 3.672117999 | 1.08E-07 | 3.77E-07 |
| IGFL2-AS1 | 1.670527738 | 8.69E-05 | 0.000190876 |
| AL627443.3 | 1.570609038 | 0.003444595 | 0.005762466 |
| AC008663.1 | -1.720711375 | 6.20E-26 | 7.87E-24 |
| RNA5SP123 | -1.503756695 | 4.92E-09 | 2.13E-08 |
| KLK5 | 2.284652249 | 3.52E-09 | 1.56E-08 |
| AJ003147.2 | 2.013356351 | 2.05E-13 | 1.83E-12 |
| RN7SL381P | -1.571844472 | 1.83E-15 | 2.35E-14 |
| CYP4F2 | -2.690746467 | 1.12E-07 | 3.88E-07 |
| ADGRB3 | -1.821289582 | 3.98E-18 | 8.55E-17 |
| MGC32805 | 1.977124814 | 0.000300374 | 0.000603856 |
| AL133387.1 | -2.048174059 | 1.37E-26 | 2.09E-24 |
| S100A2 | 2.741782326 | 8.25E-14 | 7.97E-13 |
| PCAT14 | -3.340462626 | 1.13E-10 | 6.38E-10 |
| SPRR1A | 1.639699444 | 1.15E-12 | 8.92E-12 |
| AC011294.1 | 2.366228704 | 0.000219531 | 0.000452413 |
| RBM24 | -1.968404146 | 5.34E-17 | 8.89E-16 |
| AC027698.1 | -1.763908346 | 2.00E-13 | 1.80E-12 |
| AP000654.1 | -1.968494685 | 2.96E-16 | 4.36E-15 |
| GPER1 | -2.067840025 | 2.90E-17 | 5.15E-16 |
| PENK | -2.228639802 | 0.025955676 | 0.036793176 |
| ELF5 | 1.710720458 | 8.67E-14 | 8.33E-13 |
| AC091181.1 | -2.021367274 | 2.82E-29 | 9.45E-27 |
| STRA8 | 2.489649137 | 1.62E-11 | 1.05E-10 |
| AL078582.2 | -1.639613469 | 3.09E-29 | 1.01E-26 |
| KCNG1 | 2.392804804 | 1.98E-23 | 1.34E-21 |
| FAM71C | -2.098340331 | 4.21E-11 | 2.56E-10 |
| AC109631.1 | -1.643643478 | 2.75E-16 | 4.07E-15 |
| RPL39P40 | 1.807079902 | 1.18E-13 | 1.11E-12 |
| ANXA8 | 1.565833605 | 8.40E-06 | 2.16E-05 |
| PRAC2 | 1.806951539 | 9.79E-19 | 2.33E-17 |
| UCP1 | -4.027669627 | 2.95E-07 | 9.57E-07 |
| AL022314.1 | -2.093887786 | 7.77E-18 | 1.56E-16 |
| AL136984.1 | -1.689513435 | 2.42E-06 | 6.79E-06 |
| DUSP26 | -1.706868592 | 1.47E-07 | 5.02E-07 |
| RNU6-126P | -1.57300991 | 1.97E-06 | 5.61E-06 |
| MED15P4 | -2.409004781 | 5.18E-14 | 5.19E-13 |
| AC025284.1 | -2.5389402 | 1.01E-08 | 4.15E-08 |
| AL591845.1 | -1.736920313 | 3.33E-22 | 1.70E-20 |
| S100A7 | 2.234437938 | 4.05E-19 | 1.07E-17 |
| SORCS1 | -1.835673358 | 1.89E-17 | 3.50E-16 |
| AC027575.1 | -1.799528894 | 0.000283815 | 0.000572855 |
| SNAP25 | -2.255674696 | 0.012503931 | 0.018816336 |
| CR936218.2 | -1.697895138 | 5.50E-15 | 6.50E-14 |
| LINC01488 | -2.096444603 | 8.43E-24 | 6.23E-22 |
| EN1 | 1.820386499 | 1.14E-19 | 3.40E-18 |
| ECEL1 | 3.932681143 | 1.46E-05 | 3.62E-05 |
| CYP2A7P2 | -1.516732659 | 9.93E-14 | 9.45E-13 |
| RPL6P4 | -1.548724009 | 0.000147408 | 0.00031199 |
| AC008892.1 | -1.937083112 | 1.88E-10 | 1.03E-09 |
| AC010280.1 | 1.687322609 | 2.76E-05 | 6.57E-05 |
| RGR | 1.968220017 | 2.53E-07 | 8.29E-07 |
| SCN7A | -1.905395045 | 7.24E-28 | 1.52E-25 |
| LINC00518 | 2.65360252 | 1.67E-14 | 1.82E-13 |
| CTSV | 1.565626683 | 6.68E-21 | 2.59E-19 |
| LINC01554 | 2.017654091 | 3.39E-08 | 1.28E-07 |
| TAT | -3.264586884 | 5.47E-10 | 2.76E-09 |
| SLC22A31 | 1.641022274 | 1.04E-07 | 3.62E-07 |
| SCARNA7 | -4.973074077 | 0.006293912 | 0.010031734 |
| CT62 | -1.538419324 | 1.12E-29 | 4.20E-27 |
| STK32B | -1.84315638 | 1.91E-28 | 4.64E-26 |
| PEX5L | -3.546766344 | 3.53E-25 | 3.69E-23 |
| SNORA11 | -1.660139595 | 5.24E-19 | 1.34E-17 |
| LINC02182 | 1.518221076 | 2.53E-06 | 7.07E-06 |
| KCNH6 | -2.297119246 | 0.000179755 | 0.000375155 |
| HMGN2P30 | -1.553517579 | 0.000601041 | 0.001144676 |
| AC012354.1 | 1.560907266 | 1.09E-10 | 6.17E-10 |
| AL355336.1 | -1.778003494 | 9.48E-11 | 5.43E-10 |
| AP005131.4 | -2.49482386 | 1.30E-12 | 1.00E-11 |
| CPB2 | -1.667723439 | 0.009019871 | 0.013959619 |
| SMCR5 | -1.631487753 | 0.004705841 | 0.007668869 |
| AC245041.2 | 1.720429863 | 1.59E-06 | 4.58E-06 |
| AC078850.1 | 1.812895327 | 0.001693541 | 0.002992788 |
| NDST4 | -3.20768587 | 3.87E-10 | 2.00E-09 |
| VWC2L | -3.061947458 | 6.06E-07 | 1.87E-06 |
| AL109761.1 | 1.523287032 | 3.30E-11 | 2.04E-10 |
| AC023509.5 | -1.677897416 | 0.008215663 | 0.01279723 |
| AC103982.1 | 1.852069745 | 0.005090008 | 0.008244379 |
| LCN2 | 1.838097541 | 8.60E-25 | 8.10E-23 |
| NEURL1 | -1.685193266 | 1.57E-17 | 2.95E-16 |
| GPR139 | -2.230155337 | 1.16E-18 | 2.73E-17 |
| BX119927.1 | 2.301570342 | 2.71E-16 | 4.01E-15 |
| AC122718.2 | -2.103758821 | 2.80E-08 | 1.07E-07 |
| AC018628.2 | -1.916339566 | 0.000279218 | 0.000564386 |
| GPR88 | -3.171588108 | 1.98E-21 | 8.56E-20 |
| AL078645.1 | -1.73998517 | 1.57E-11 | 1.02E-10 |
| WNK4 | -1.713609326 | 1.49E-20 | 5.38E-19 |
| KCNMA1-AS3 | -1.667935708 | 8.53E-20 | 2.60E-18 |
| TRIML2 | 2.218597655 | 3.42E-17 | 5.96E-16 |
| AC009303.2 | -1.686992802 | 0.001302259 | 0.002347482 |
| ALB | -3.693017158 | 1.02E-07 | 3.56E-07 |
| UGT8 | 1.581175126 | 7.68E-08 | 2.74E-07 |
| HNRNPA1P68 | -1.876289156 | 0.034993425 | 0.04831617 |
| LINC02306 | -3.251110465 | 7.61E-18 | 1.53E-16 |
| CYP2A7 | -4.085058777 | 1.04E-15 | 1.40E-14 |
| LINC00707 | 3.462362374 | 0.000138875 | 0.000295039 |
| RAX | 1.615686769 | 3.43E-07 | 1.10E-06 |
| IGSF1 | -1.893459767 | 0.001628442 | 0.002888708 |
| PPP1R1B | 1.835320887 | 6.42E-06 | 1.69E-05 |
| MAS1L | -1.682348186 | 8.79E-12 | 5.95E-11 |
| CXCL5 | 1.911858746 | 1.89E-08 | 7.43E-08 |
| GPM6A | -3.095281765 | 1.23E-09 | 5.84E-09 |
| PRSS33 | 3.163296989 | 6.84E-06 | 1.79E-05 |
| EPGN | 1.671827458 | 6.65E-07 | 2.04E-06 |
| TMEM75 | -2.129906243 | 9.76E-08 | 3.42E-07 |
| MEG8 | -1.786361374 | 0.013432561 | 0.020081842 |
| AC036108.1 | -1.782973485 | 1.19E-21 | 5.43E-20 |
| STH | -2.058897642 | 8.24E-24 | 6.11E-22 |
| PDZPH1P | -3.559751854 | 8.98E-14 | 8.61E-13 |
| AC034213.1 | 3.703988129 | 5.09E-11 | 3.04E-10 |
| BPI | 1.58320814 | 2.36E-05 | 5.70E-05 |
| MARK2P8 | -1.690221217 | 0.012180636 | 0.018380105 |
| AC097526.1 | 1.727454778 | 0.000758318 | 0.001420397 |
| RGS22 | -1.791810752 | 1.08E-28 | 2.88E-26 |
| TEX19 | -2.135313609 | 0.008205663 | 0.01278236 |
| ADAM29 | -1.549431467 | 0.000639255 | 0.001211231 |
| BCL2 | -1.774101273 | 1.60E-49 | 2.25E-45 |
| SNORA12 | -3.65348692 | 0.000169076 | 0.000354548 |
| C8orf86 | -1.935160126 | 4.94E-20 | 1.59E-18 |
| RN7SL648P | -2.760831966 | 0.005665631 | 0.00910487 |
| SLURP1 | 2.006414111 | 4.56E-22 | 2.27E-20 |
| ERVFRD-1 | -2.204904651 | 6.19E-11 | 3.65E-10 |
| KRT6C | 4.048869353 | 4.70E-10 | 2.39E-09 |
| INSM1 | -2.567553941 | 0.004392072 | 0.007194572 |
| MANCR | 1.890478008 | 1.45E-06 | 4.20E-06 |
| UNC13C | -1.661176491 | 1.16E-13 | 1.09E-12 |
| DUXB | -1.988900053 | 1.57E-09 | 7.32E-09 |
| AMTN | 1.753126661 | 7.55E-10 | 3.73E-09 |
| PPFIA1P1 | -1.970905547 | 0.000411628 | 0.000808366 |
| GPR26 | -3.151907753 | 0.023508046 | 0.033609337 |
| NANOGP1 | 3.24900033 | 5.12E-16 | 7.32E-15 |
| GSG1L | -2.08118299 | 0.000321577 | 0.000643313 |
| TPSG1 | -2.148985771 | 1.38E-21 | 6.23E-20 |
| EPHX3 | 1.942554637 | 6.17E-06 | 1.62E-05 |
| PCARE | 1.612472203 | 0.001890079 | 0.003309078 |
| AC096589.2 | -1.871398473 | 9.91E-15 | 1.12E-13 |
| GPR87 | 1.599243206 | 0.000896266 | 0.001657954 |
| AC093866.1 | -1.553591046 | 3.52E-20 | 1.17E-18 |
| ANKRD1 | 1.580505404 | 7.84E-07 | 2.38E-06 |
| KRT17P1 | 1.871856424 | 6.61E-07 | 2.03E-06 |
| AL035603.1 | 1.527086283 | 3.76E-08 | 1.41E-07 |
| MSLN | 1.671306972 | 3.22E-11 | 1.99E-10 |
| GRIA1 | -4.313797177 | 6.06E-26 | 7.73E-24 |
| ZFP57 | 1.696625953 | 8.79E-10 | 4.30E-09 |
| AC011120.1 | -1.849158931 | 0.001459279 | 0.002608501 |
| MPRIP-AS1 | -1.536228754 | 0.001146262 | 0.002085762 |
| LYPD6 | -1.798692971 | 2.94E-28 | 6.90E-26 |
| CPB1 | -5.377139643 | 2.16E-18 | 4.88E-17 |
| AL136982.3 | -1.522540158 | 0.003400927 | 0.005694486 |
| KRT6A | 3.291002013 | 1.41E-14 | 1.55E-13 |
| KRT16P6 | 2.683436838 | 0.000124973 | 0.000267258 |
| GSTA5 | 1.915987267 | 5.93E-11 | 3.51E-10 |
| AL592078.2 | -1.804242395 | 7.74E-07 | 2.35E-06 |
| AC055807.1 | -3.167111198 | 2.94E-13 | 2.54E-12 |
| RFX6 | -2.538480202 | 2.03E-11 | 1.30E-10 |
| LONRF2 | -1.503895976 | 1.55E-29 | 5.67E-27 |
| AL022323.1 | -2.013158227 | 1.60E-07 | 5.44E-07 |
| FGF10 | -2.216049939 | 6.54E-15 | 7.65E-14 |
| ACTG2 | 1.603737462 | 0.025569139 | 0.036305523 |
| UGT2B7 | 2.082788423 | 5.04E-11 | 3.02E-10 |
| NTS | -6.124283828 | 0.00018222 | 0.000379906 |
| FABP5P7 | 1.611777338 | 1.49E-13 | 1.37E-12 |
| CCNYL7 | -1.917230418 | 5.29E-14 | 5.29E-13 |
| OLIG2 | 1.586324682 | 5.08E-08 | 1.86E-07 |
| AC022336.3 | -1.601153266 | 2.35E-12 | 1.74E-11 |
| AP001574.1 | 1.541347783 | 5.43E-20 | 1.73E-18 |
| AC138409.1 | -2.139358326 | 2.59E-13 | 2.27E-12 |
| LARGE-AS1 | -1.886345587 | 3.95E-05 | 9.18E-05 |
| NPTX1 | -2.813685006 | 1.95E-06 | 5.56E-06 |
| PHF21B | -2.409653677 | 2.88E-19 | 7.89E-18 |
| KRT5 | 1.704612119 | 0.000120596 | 0.000258565 |
| TAS2R50 | -1.614816832 | 1.92E-06 | 5.47E-06 |
| TNRC18P1 | -1.923484701 | 2.48E-29 | 8.41E-27 |
| AL356515.1 | 1.63187032 | 9.79E-07 | 2.93E-06 |
| CLEC4O | -1.505231442 | 2.00E-08 | 7.83E-08 |
| SLC16A12 | -1.676565661 | 8.35E-20 | 2.55E-18 |
| MOG | 3.776881963 | 9.12E-05 | 0.000199654 |
| AL035409.1 | -1.561760318 | 0.001424251 | 0.002550904 |
| LINC01559 | 2.665451825 | 5.17E-07 | 1.62E-06 |
| THSD4-AS1 | -3.384186683 | 6.33E-25 | 6.26E-23 |
| CA9 | 2.029857389 | 4.14E-26 | 5.55E-24 |
| AC079296.1 | -2.281173524 | 1.50E-28 | 3.88E-26 |
| AP006333.2 | -1.521908056 | 5.48E-06 | 1.45E-05 |
| MEPE | -3.500908025 | 0.020759541 | 0.029991799 |
| PI3 | 3.202334637 | 6.87E-14 | 6.74E-13 |
| AC105328.1 | -1.657200633 | 1.74E-29 | 6.21E-27 |
| HOXB-AS4 | 1.930247176 | 4.07E-09 | 1.79E-08 |
| STC2 | -1.824551942 | 1.43E-22 | 8.01E-21 |
| AC139792.1 | -1.793584023 | 0.001290886 | 0.002328769 |
| CYP2A6 | -3.444132284 | 1.29E-11 | 8.47E-11 |
| AL392083.1 | -1.801575763 | 2.71E-14 | 2.84E-13 |
| AC022784.1 | 2.519816899 | 7.71E-20 | 2.37E-18 |
| WFDC13 | -1.579071986 | 0.00141159 | 0.002530157 |
| SLCO4A1-AS1 | 1.778660009 | 1.83E-12 | 1.37E-11 |
| CCNYL2 | 1.568425793 | 0.000379529 | 0.000749823 |
| C6orf15 | 3.924711091 | 2.73E-08 | 1.05E-07 |
| ST7-AS2 | -3.547745447 | 0.007878774 | 0.012319465 |
| AKR7A3 | -1.543047159 | 6.21E-20 | 1.95E-18 |
| PCAT5 | 1.941174205 | 3.53E-09 | 1.56E-08 |
| MTND1P8 | -1.515049603 | 5.48E-11 | 3.26E-10 |
| RAET1L | 2.405999708 | 4.26E-35 | 6.67E-32 |
| AC245884.9 | 1.820496716 | 0.000118505 | 0.00025441 |
| CLDN19 | -1.634757079 | 2.90E-08 | 1.11E-07 |
| BANCR | 3.019956767 | 4.41E-05 | 0.000101822 |
| AC021134.1 | -2.046244397 | 1.72E-08 | 6.82E-08 |
| MRPS30-DT | -2.234049137 | 1.14E-24 | 1.05E-22 |
| AC020656.2 | -2.039601385 | 1.03E-10 | 5.87E-10 |
| LCN10 | -1.735685527 | 7.30E-06 | 1.90E-05 |
| CHODL | 2.056807213 | 1.61E-10 | 8.90E-10 |
| AL020995.1 | -1.551123936 | 0.003391075 | 0.005679003 |
| AC120349.3 | -1.528532727 | 0.001578341 | 0.002806017 |
| KLHDC7A | -1.923629947 | 5.20E-13 | 4.30E-12 |
| SERPINA11 | -1.572505752 | 7.34E-19 | 1.81E-17 |
| AP001972.3 | -1.963817721 | 0.000810979 | 0.001511299 |
| AC113346.1 | 2.431637805 | 4.28E-07 | 1.36E-06 |
| SHANK2-AS1 | -2.20356611 | 2.76E-05 | 6.57E-05 |
| IGSF23 | 1.559352002 | 3.99E-11 | 2.43E-10 |
| AC010261.2 | -1.695815917 | 9.78E-12 | 6.56E-11 |
| TPRG1 | -1.71228999 | 1.52E-23 | 1.06E-21 |
| MAGEA4 | 2.329643246 | 7.77E-10 | 3.83E-09 |
| AC008937.2 | -2.111560838 | 2.82E-11 | 1.77E-10 |
| SCARNA21 | -3.392017094 | 0.000688595 | 0.001298171 |
| NFE4 | 2.077391329 | 0.001432045 | 0.002563391 |
| AC011503.1 | -1.886265196 | 0.000324985 | 0.000649533 |
| LINC01085 | -1.640167356 | 1.32E-11 | 8.70E-11 |
| CSMD3 | -1.53410902 | 5.22E-05 | 0.000119072 |
| ARHGAP16P | -2.523927843 | 1.02E-08 | 4.18E-08 |
| AP005131.3 | -1.610736518 | 2.33E-17 | 4.22E-16 |
| KCNE5 | 2.065514767 | 0.000108476 | 0.000234631 |
| KRT17 | 1.66347127 | 4.90E-07 | 1.54E-06 |
| RERG-AS1 | -2.852106639 | 9.99E-23 | 5.75E-21 |
| IL36RN | 2.175862727 | 6.55E-14 | 6.46E-13 |
| KLHL34 | 1.951352555 | 0.003077474 | 0.005192086 |
| RN7SL395P | -1.647669891 | 0.001080112 | 0.001974063 |
| VWA5B1 | -1.637498286 | 4.13E-12 | 2.93E-11 |
| ANK1 | -2.521944863 | 0.004701746 | 0.007662639 |
| ZFP42 | 2.840366299 | 7.64E-05 | 0.000169421 |
| RAB3C | -1.785179564 | 1.10E-18 | 2.59E-17 |
| GPR79 | -1.511525931 | 1.42E-08 | 5.72E-08 |
| HORMAD1 | 1.787194717 | 1.45E-07 | 4.93E-07 |
| FMO6P | 2.393387504 | 2.06E-09 | 9.45E-09 |
| SNORA54 | -3.485471505 | 0.000122546 | 0.000262425 |
| RTBDN | -2.018649567 | 0.001388386 | 0.002492372 |
| MYT1 | -2.661329589 | 7.21E-14 | 7.05E-13 |
| AC093297.1 | -3.80163985 | 8.29E-25 | 7.90E-23 |
| ANKS4B | 1.786920685 | 5.75E-05 | 0.000130197 |
| BARX1 | 2.223219082 | 3.47E-12 | 2.49E-11 |
| IGDCC3 | -2.229469948 | 2.70E-20 | 9.28E-19 |
| LINC02159 | 2.083017215 | 3.63E-15 | 4.43E-14 |
| QRSL1P3 | -1.761882578 | 0.000393276 | 0.000774864 |
| S100A9 | 2.533005204 | 8.50E-29 | 2.40E-26 |
| GLRA3 | -2.096561947 | 5.16E-11 | 3.08E-10 |
| AL136146.1 | -2.430358163 | 1.05E-12 | 8.22E-12 |
| KRT81 | 2.360888179 | 1.62E-11 | 1.05E-10 |
| LBP | 1.934410947 | 7.96E-10 | 3.92E-09 |
| NLRP10 | 1.868834665 | 0.015642639 | 0.023104657 |
| C1orf167 | -2.522182508 | 3.93E-11 | 2.40E-10 |
| LINC02869 | 2.17180578 | 3.49E-13 | 2.98E-12 |
| SCARNA5 | -5.759235678 | 1.02E-05 | 2.59E-05 |
| RPS20P15 | -2.136182885 | 0.007002346 | 0.011051485 |
| AC016995.1 | 1.773740829 | 5.82E-15 | 6.86E-14 |
| CDKN2A | 1.606076481 | 9.31E-09 | 3.86E-08 |
| AC008592.4 | 1.569774928 | 5.69E-13 | 4.67E-12 |
| RN7SL181P | -1.87139691 | 5.50E-10 | 2.77E-09 |
| PSORS1C2 | 2.412939992 | 1.36E-15 | 1.79E-14 |
| KLK8 | 2.50106726 | 3.39E-09 | 1.51E-08 |
| CXCL17 | 1.853859712 | 1.64E-10 | 9.05E-10 |
| LINC02577 | 1.717884647 | 4.28E-10 | 2.19E-09 |
| AC109630.1 | -4.011169005 | 2.06E-12 | 1.54E-11 |
| AC012123.1 | -1.653019703 | 3.86E-11 | 2.35E-10 |
| AP001783.1 | 1.606497868 | 3.77E-11 | 2.30E-10 |
| F2 | -2.219132979 | 0.015719429 | 0.023210089 |
| ANKRD44-IT1 | -1.730343529 | 0.027094656 | 0.038272933 |
| RN7SKP275 | -2.267005294 | 5.94E-25 | 5.94E-23 |
| LINC00393 | 1.818257448 | 1.31E-10 | 7.34E-10 |
| TMEM72 | 1.636800038 | 0.00326165 | 0.005478212 |
| PAH | -1.607851845 | 2.83E-09 | 1.27E-08 |
| NAV3 | -2.233277239 | 1.71E-29 | 6.17E-27 |
| L1CAM | 1.562238956 | 7.12E-11 | 4.16E-10 |
| LINC02765 | 1.635944837 | 2.66E-09 | 1.20E-08 |
| AC018697.1 | 2.013959297 | 2.52E-06 | 7.05E-06 |
| AL034346.1 | 1.987095527 | 0.021886287 | 0.031482419 |
| LINC01667 | 2.066893013 | 1.26E-07 | 4.33E-07 |
| AP003059.2 | -1.643662072 | 3.46E-09 | 1.53E-08 |
| FERMT1 | 1.518918356 | 4.49E-08 | 1.66E-07 |
| AC068580.2 | 1.566123485 | 2.92E-14 | 3.05E-13 |
| AC103876.1 | -1.694633371 | 8.67E-06 | 2.23E-05 |
| AC022113.1 | -2.047736141 | 8.98E-20 | 2.72E-18 |
| AP001053.1 | 1.591348793 | 3.69E-12 | 2.64E-11 |
| AC097658.3 | -2.107039808 | 0.018963039 | 0.027578775 |
| AC008764.10 | -1.884073015 | 0.004085496 | 0.006734122 |
| HNRNPA3P7 | -1.505397321 | 0.000549226 | 0.001053759 |
| OTOG | 3.434077803 | 3.02E-10 | 1.59E-09 |
| TACR3 | -1.61034045 | 1.88E-06 | 5.37E-06 |
| NSG2 | -5.255167612 | 2.50E-14 | 2.63E-13 |
| PCDH8 | 1.870980763 | 1.95E-06 | 5.55E-06 |
| RN7SL128P | -3.41798358 | 0.011233334 | 0.01708221 |
| LHFPL4 | -2.227155434 | 5.50E-05 | 0.000124786 |
| AL035106.1 | -1.500190234 | 2.69E-24 | 2.21E-22 |
| SCARNA9 | -1.584501637 | 0.000117718 | 0.000252933 |
| INSYN2A | -2.635014682 | 1.06E-23 | 7.71E-22 |
| RN7SL314P | -1.801941188 | 8.90E-21 | 3.34E-19 |
| RLBP1 | 1.517525099 | 0.001302759 | 0.002348082 |
| AL596087.2 | 1.869944266 | 1.14E-06 | 3.36E-06 |
| AC093766.1 | -2.157542374 | 9.17E-09 | 3.81E-08 |
| RN7SL338P | -1.586241225 | 0.000381358 | 0.00075333 |
| SLC26A3 | -2.182303512 | 1.26E-21 | 5.71E-20 |
| UNC93A | 1.590723362 | 1.02E-12 | 8.01E-12 |
| C1orf94 | 3.37086448 | 2.15E-08 | 8.40E-08 |
| KRT8P41 | -1.769263553 | 2.81E-19 | 7.72E-18 |
| ETV3L | 1.655240937 | 0.000161426 | 0.00033962 |
| AL133444.1 | -1.766011964 | 0.000629434 | 0.001194067 |
| TMEM26-AS1 | -2.147775129 | 5.65E-23 | 3.42E-21 |
| TSPYL6 | -2.743742705 | 0.007183729 | 0.011314303 |
| DNAJC12 | -1.653093873 | 7.23E-26 | 9.02E-24 |
| AP005131.1 | -1.89023096 | 1.34E-13 | 1.24E-12 |
| AF127577.6 | -2.184158458 | 1.08E-11 | 7.17E-11 |
| STAC | 1.66672456 | 1.23E-08 | 4.98E-08 |
| AL732437.2 | 1.541249552 | 0.001398826 | 0.002509036 |
| PBX1-AS1 | -2.354792368 | 9.32E-05 | 0.000203833 |
| LINC02532 | 1.841335135 | 0.000207894 | 0.000429964 |
| KRT83 | 1.805518956 | 2.06E-14 | 2.19E-13 |
| AC129502.1 | -2.083969628 | 1.72E-08 | 6.83E-08 |
| AC114501.1 | -2.118234858 | 1.19E-10 | 6.70E-10 |
| B4GALNT2 | 1.671139789 | 0.002944385 | 0.004987257 |
| AC136621.1 | 1.922885703 | 1.16E-08 | 4.71E-08 |
| AC027018.1 | -1.99816412 | 0.008839807 | 0.013704279 |
| PTPRQ | -2.074540416 | 4.84E-07 | 1.52E-06 |
| AMER3 | -5.624741768 | 3.64E-06 | 9.92E-06 |
| SNORA63C | -1.784322972 | 0.026872371 | 0.037979888 |
| AL353583.1 | -2.206230933 | 1.41E-07 | 4.83E-07 |
| AP003783.1 | -1.999838732 | 0.000153677 | 0.000324308 |
| SLC4A10 | -2.462654118 | 5.39E-08 | 1.97E-07 |
| LHCGR | -2.083003575 | 0.001836254 | 0.003223857 |
| NRIP3 | -2.101487144 | 6.50E-14 | 6.41E-13 |
| BSN | -1.552326677 | 1.02E-25 | 1.22E-23 |
| NTRK2 | -1.604549042 | 3.08E-13 | 2.65E-12 |
| LEMD1-DT | 1.710195926 | 1.20E-05 | 3.01E-05 |
| KNOP1P2 | -1.556259548 | 3.60E-10 | 1.87E-09 |
| NR0B2 | -1.866747083 | 0.001627557 | 0.002887684 |
| AFF3 | -2.04358918 | 2.51E-41 | 1.01E-37 |
| TMEM161BP1 | -1.689248772 | 7.07E-19 | 1.75E-17 |
| PGBD5 | 1.856009106 | 3.28E-06 | 8.99E-06 |
| AL353748.2 | -1.797180894 | 3.90E-19 | 1.04E-17 |
| NRXN3 | -1.570523035 | 1.22E-20 | 4.44E-19 |
| PLA2G4E | 2.397742766 | 1.30E-06 | 3.80E-06 |
| P2RX2 | 1.51334823 | 7.40E-05 | 0.000164524 |
| LINC01956 | 1.806082109 | 4.60E-15 | 5.51E-14 |
| BNIP3P42 | -1.554796835 | 7.91E-06 | 2.05E-05 |
| IGF1R | -1.516386076 | 8.04E-32 | 5.08E-29 |
| AC005050.2 | 1.851762543 | 1.20E-15 | 1.59E-14 |
| AC012404.1 | -2.774280596 | 1.62E-05 | 4.01E-05 |
| GLIS3-AS1 | 1.596631408 | 1.72E-08 | 6.83E-08 |
| MIR2052HG | -1.669172066 | 1.31E-10 | 7.34E-10 |
| NCCRP1 | 2.334722034 | 5.71E-21 | 2.24E-19 |
| GJB3 | 2.14520639 | 1.52E-13 | 1.40E-12 |
| AL360169.2 | -1.578230154 | 1.25E-05 | 3.13E-05 |
| ART3 | 2.166973331 | 3.13E-18 | 6.83E-17 |
| GABRP | 1.945119244 | 4.14E-10 | 2.13E-09 |
| CHGA | -9.074382049 | 1.43E-05 | 3.56E-05 |
| FOXCUT | 1.839356761 | 4.71E-13 | 3.93E-12 |
| DACH1 | -1.55349173 | 1.10E-27 | 2.16E-25 |
| AL359258.1 | -1.698339066 | 5.43E-08 | 1.99E-07 |
| AC134349.2 | -1.524754852 | 0.026826246 | 0.037916599 |
| CDH7 | -1.891018138 | 7.94E-13 | 6.36E-12 |
| AL512283.3 | -1.603431336 | 3.60E-15 | 4.40E-14 |
| AC022196.1 | -2.901593011 | 9.19E-18 | 1.81E-16 |
| LINC01285 | 1.62264283 | 4.00E-13 | 3.38E-12 |
| AC004969.1 | -1.998642039 | 9.32E-17 | 1.49E-15 |
| CAPNS2 | 1.722306379 | 0.000789191 | 0.001473425 |
| ACTL8 | 1.83974899 | 9.64E-17 | 1.54E-15 |
| SBK2 | -1.674133019 | 5.25E-08 | 1.92E-07 |
| CHGB | -6.538531259 | 8.92E-06 | 2.29E-05 |
| CLRN1 | -1.523619162 | 4.80E-07 | 1.51E-06 |
| PGR | -1.565380903 | 1.59E-26 | 2.41E-24 |
| AC012085.2 | -1.841859895 | 7.60E-13 | 6.10E-12 |
| AL133368.1 | -2.086875143 | 1.52E-07 | 5.16E-07 |
| CES1P1 | -1.932441765 | 0.00271839 | 0.004634273 |
| AC010967.1 | 1.852699255 | 1.20E-16 | 1.89E-15 |
| AL132708.1 | -2.166167588 | 1.44E-18 | 3.33E-17 |
| AC092384.3 | -1.756471782 | 7.50E-18 | 1.52E-16 |
| SCG2 | -2.718293272 | 5.09E-06 | 1.36E-05 |
| AL117340.1 | -1.795625673 | 0.000112519 | 0.000242669 |
| IFITM5 | -1.598316562 | 5.62E-05 | 0.000127365 |
| CADM2 | -1.956985246 | 1.76E-25 | 1.99E-23 |
| KLK6 | 3.025324171 | 2.49E-15 | 3.15E-14 |
| LRP2 | -1.938656258 | 1.92E-17 | 3.56E-16 |
| HAR1A | -1.866447174 | 1.81E-05 | 4.44E-05 |
| AC080037.2 | 2.09777061 | 3.37E-11 | 2.08E-10 |
| ADGRD2 | -2.857993951 | 0.02793418 | 0.039356212 |
| PHGR1 | -2.138110125 | 4.34E-23 | 2.70E-21 |
| ANTXRL | 2.068553471 | 0.001521986 | 0.002712502 |
| CCKBR | 2.383789163 | 1.11E-07 | 3.86E-07 |
| SLC6A2 | 2.421622173 | 1.43E-08 | 5.74E-08 |
| TRH | -2.209096081 | 3.74E-20 | 1.24E-18 |
| GJB5 | 1.827529583 | 5.62E-08 | 2.05E-07 |
| RN7SL359P | -3.716995924 | 6.39E-12 | 4.42E-11 |
| Z98257.1 | 1.566137169 | 4.78E-08 | 1.76E-07 |
| PRICKLE2-AS3 | -1.623352651 | 0.00033682 | 0.000671186 |
| SPRR1B | 3.053014134 | 5.21E-15 | 6.20E-14 |
| AC023421.1 | -2.029866064 | 6.79E-10 | 3.38E-09 |
| AC073636.1 | -1.594160742 | 2.67E-05 | 6.38E-05 |
| PRDX3P4 | -1.518098626 | 2.69E-06 | 7.48E-06 |
| GP2 | -1.679063529 | 2.35E-17 | 4.24E-16 |
| GRIK1-AS1 | -1.985910598 | 3.79E-13 | 3.23E-12 |
| AL133284.1 | -2.350235493 | 5.27E-06 | 1.40E-05 |
| AC125603.1 | -3.116464256 | 3.56E-12 | 2.55E-11 |
| FSIP1 | -1.517458352 | 8.84E-27 | 1.42E-24 |
| FAM83A | 1.584284003 | 1.23E-06 | 3.61E-06 |
| RN7SL40P | -1.994375109 | 0.000983444 | 0.001808061 |
| SPATA46 | -1.625372483 | 8.93E-17 | 1.43E-15 |
| PRSS2 | -1.909724078 | 0.035507486 | 0.048963558 |
| AC022973.2 | -1.859711016 | 0.011762504 | 0.017803307 |
| SYP | -1.61571854 | 2.75E-07 | 8.99E-07 |
| LINC02323 | 1.717457548 | 7.52E-10 | 3.72E-09 |
| TBL1XR1-AS1 | -1.849762333 | 7.88E-05 | 0.000174414 |
| AC018978.1 | 3.307709224 | 3.42E-11 | 2.10E-10 |
| SEC24AP1 | -1.83530738 | 2.43E-14 | 2.57E-13 |
| WNT6 | 1.803154312 | 1.39E-09 | 6.57E-09 |
| SNX18P13 | -1.643425405 | 1.41E-11 | 9.19E-11 |
| KCNJ3 | -2.355254313 | 6.17E-25 | 6.12E-23 |
| FUT3 | 1.546979898 | 1.31E-33 | 1.27E-30 |
| FADS6 | 2.112775292 | 3.24E-06 | 8.91E-06 |
| SERPINB4 | 3.446293091 | 9.85E-10 | 4.78E-09 |
| IL20RB | 1.805543359 | 3.50E-14 | 3.61E-13 |
| RGS7 | -1.737405813 | 7.98E-12 | 5.43E-11 |
| ANO2 | -1.531216444 | 1.39E-15 | 1.82E-14 |
| SCUBE2 | -1.520180268 | 5.60E-22 | 2.76E-20 |
| KIAA0087 | -1.991751911 | 2.83E-15 | 3.54E-14 |
| AL079343.1 | -2.875831571 | 3.64E-23 | 2.32E-21 |
| SLC7A2-IT1 | -1.55539766 | 2.63E-11 | 1.65E-10 |
| SCUBE1 | -1.513252377 | 1.46E-19 | 4.25E-18 |
| RN7SL767P | -2.346760084 | 2.02E-11 | 1.29E-10 |
| AL359740.1 | -1.680898994 | 0.004966626 | 0.008058434 |
| GNAQP1 | -1.646761982 | 2.93E-18 | 6.43E-17 |
| SEZ6L | -3.285744997 | 3.01E-25 | 3.21E-23 |
| AL139081.1 | -2.520419546 | 3.20E-19 | 8.71E-18 |
| LINC00589 | -1.624533338 | 3.59E-05 | 8.39E-05 |
| AC093838.1 | -1.930898015 | 5.05E-25 | 5.11E-23 |
| LINC00163 | 1.702486412 | 0.000410766 | 0.000806842 |
| LINC02065 | 1.57778 | 1.97E-07 | 6.60E-07 |
| MYH1 | -2.457402757 | 3.65E-05 | 8.53E-05 |
| SH3PXD2A-AS1 | 1.886544136 | 0.001297647 | 0.002339993 |
| SIAH3 | -2.102576226 | 2.67E-05 | 6.37E-05 |
| CEACAM16 | -1.630867917 | 7.13E-11 | 4.16E-10 |
| NPSR1-AS1 | -2.152655536 | 6.37E-08 | 2.30E-07 |
| SBSN | 2.499541636 | 1.22E-23 | 8.64E-22 |
| AC091804.1 | -1.695468616 | 1.75E-07 | 5.89E-07 |
| TEX15 | 1.572959081 | 8.92E-05 | 0.000195704 |
| TMEM145 | -1.56783461 | 1.99E-17 | 3.68E-16 |
| OLIG1 | 1.526083034 | 0.006945718 | 0.01097133 |
| LY6D | 2.619564611 | 1.82E-11 | 1.17E-10 |
| PCSK9 | 1.616916003 | 0.000100112 | 0.000217737 |
| AC005803.1 | -1.524567235 | 9.16E-07 | 2.75E-06 |
| LINC01768 | -1.871650023 | 5.07E-13 | 4.21E-12 |
| RPL26P35 | -1.547537358 | 0.000690612 | 0.001301429 |
| LINC02747 | -2.032285409 | 3.90E-23 | 2.46E-21 |
| AC017002.3 | 1.915467294 | 1.12E-11 | 7.42E-11 |
| AL031667.2 | -1.753034284 | 9.52E-10 | 4.63E-09 |
| SLC22A10 | -1.754762009 | 1.51E-12 | 1.15E-11 |
| AC098679.5 | -1.921667137 | 8.19E-22 | 3.84E-20 |
| ZDHHC22 | -1.976957805 | 4.79E-08 | 1.77E-07 |
| AC053513.1 | 1.938140063 | 1.05E-11 | 7.00E-11 |
| AC093627.1 | 2.214730336 | 1.58E-11 | 1.02E-10 |
| RASGEF1C | 2.36142527 | 2.95E-11 | 1.84E-10 |
| C5orf64 | -2.304452497 | 4.71E-15 | 5.65E-14 |
| NPY6R | -4.160034229 | 3.91E-10 | 2.02E-09 |
| AC016831.4 | -2.972436946 | 0.005409614 | 0.008719942 |
| SLC18A1 | -2.872342668 | 0.00014156 | 0.0003002 |
| PCP4L1 | 2.042566562 | 5.80E-15 | 6.85E-14 |
| PRSS3 | 1.619008104 | 1.10E-11 | 7.29E-11 |
| AL138999.2 | -1.873727732 | 2.37E-10 | 1.27E-09 |
| OR7E136P | 1.574805131 | 9.27E-09 | 3.84E-08 |
| CST9 | -3.6482103 | 5.60E-35 | 8.31E-32 |
| TLR8-AS1 | 1.952135636 | 0.004331536 | 0.007102429 |
| SLIT1 | -2.467904049 | 3.13E-06 | 8.63E-06 |
| SNORA7B | -1.846256376 | 0.025055652 | 0.035621304 |
| AC025423.3 | -2.770176403 | 5.87E-10 | 2.95E-09 |
| RNVU1-29 | -5.087218173 | 0.017122187 | 0.025119376 |
| KLK7 | 2.450700187 | 1.20E-09 | 5.72E-09 |
| AC119424.1 | 1.78775975 | 0.00052791 | 0.001016598 |
| PGLYRP4 | 2.268348106 | 7.03E-25 | 6.85E-23 |
| EEF1A1P32 | -2.942920263 | 0.001132418 | 0.002062036 |
| AK3P5 | -2.171643369 | 4.46E-05 | 0.000102893 |
| PTPN2P1 | -1.583782366 | 0.033750447 | 0.046785675 |
| SLC28A2 | -1.946951014 | 2.53E-15 | 3.19E-14 |
| AL360268.1 | -1.517951427 | 0.000296317 | 0.000596211 |
| CASC8 | 2.212527475 | 2.15E-13 | 1.92E-12 |
| AC115837.1 | -2.161581794 | 9.04E-11 | 5.19E-10 |
| SLC16A6 | -1.583105543 | 1.42E-25 | 1.63E-23 |
| CASC16 | -1.56999252 | 6.20E-06 | 1.63E-05 |
| CLCP2 | -1.540272043 | 3.51E-11 | 2.15E-10 |
| AL589182.1 | 1.995815954 | 1.47E-10 | 8.16E-10 |
| GRPR | -1.737285825 | 2.48E-20 | 8.62E-19 |
| SLC27A2 | -1.85515388 | 1.58E-22 | 8.74E-21 |
| AC061961.1 | -2.814252799 | 2.92E-22 | 1.51E-20 |
| AC133485.7 | -3.211107705 | 0.033938294 | 0.047011378 |
| CTCFL | -1.63959406 | 0.018269712 | 0.026639193 |
| FOXG1 | 1.805766519 | 0.008447227 | 0.013136142 |
| UPK2 | 1.614747636 | 1.72E-21 | 7.59E-20 |
| SLC15A1 | 2.468598789 | 8.37E-20 | 2.56E-18 |
| CCDC175 | -1.763979429 | 4.77E-08 | 1.76E-07 |
| AL512378.1 | -1.839276535 | 4.53E-15 | 5.45E-14 |
| MPPED1 | -2.635486847 | 7.96E-16 | 1.10E-14 |
| CST9L | -2.693111019 | 2.53E-23 | 1.66E-21 |
| AF127577.2 | -1.747063588 | 2.43E-08 | 9.39E-08 |
| GAL | 1.57039909 | 2.23E-19 | 6.21E-18 |
| DSG3 | 1.808275999 | 0.013699924 | 0.020455504 |
| AL033384.1 | 1.598812977 | 2.46E-13 | 2.16E-12 |
| MYH2 | -3.430508887 | 0.003977854 | 0.006570676 |
| TAC1 | -2.169224401 | 2.09E-17 | 3.83E-16 |
| SLC2A3P2 | -1.890793385 | 8.44E-23 | 4.91E-21 |
| PVALB | -2.071452904 | 3.33E-07 | 1.07E-06 |
| LINC01634 | 2.484053951 | 2.36E-07 | 7.79E-07 |
| AFP | -2.166144782 | 3.59E-10 | 1.87E-09 |
| NIPAL4 | 1.759829709 | 8.06E-08 | 2.86E-07 |
| AL136305.1 | -2.974966475 | 1.35E-16 | 2.11E-15 |
| LINC02732 | 2.724180326 | 3.67E-19 | 9.87E-18 |
| GRAMD4P8 | -1.89588112 | 5.84E-25 | 5.89E-23 |
| AC058791.1 | -2.348385922 | 0.026318044 | 0.037259981 |
| USH1C | 2.527630085 | 0.031723343 | 0.044217136 |
| KCND3 | -1.640620364 | 4.76E-25 | 4.88E-23 |
| SCEL | 1.775282272 | 7.87E-15 | 9.04E-14 |
| CACNG6 | -2.097153688 | 0.009383387 | 0.014480059 |
| AC008571.2 | -1.89768636 | 1.50E-09 | 7.05E-09 |
| S100A7A | 2.265724757 | 1.65E-14 | 1.80E-13 |
| HAO2 | -3.320242816 | 1.95E-10 | 1.06E-09 |
| AC021231.1 | -1.561896687 | 6.01E-12 | 4.17E-11 |
| AC013480.1 | -1.849451868 | 6.89E-11 | 4.04E-10 |
| SLC6A14 | 1.644465609 | 1.71E-12 | 1.29E-11 |
| RN7SL449P | -1.608651344 | 4.00E-14 | 4.08E-13 |
| IL22RA2 | 1.670741616 | 1.38E-14 | 1.52E-13 |
| RERG-IT1 | -1.755854705 | 8.80E-31 | 4.43E-28 |
| PROK2 | 1.644103559 | 0.000679826 | 0.001283013 |
| MMP7 | 1.749947582 | 1.67E-09 | 7.79E-09 |
| AC092127.1 | -2.084695527 | 1.52E-08 | 6.08E-08 |
| AL357146.1 | 1.811548723 | 6.29E-11 | 3.70E-10 |
| RARRES1 | 1.943192224 | 4.17E-16 | 6.02E-15 |
| DMRT1 | 1.913505359 | 7.27E-22 | 3.48E-20 |
| LINP1 | 2.490359833 | 1.37E-09 | 6.48E-09 |
| PF4V1 | 1.599627146 | 1.39E-08 | 5.58E-08 |
| KCNMA1-AS1 | -1.61626146 | 5.53E-20 | 1.76E-18 |
| SEZ6 | -5.071838884 | 4.79E-15 | 5.73E-14 |
| CALML5 | 1.64379733 | 3.56E-16 | 5.19E-15 |
| FER1L6 | -1.527675154 | 5.25E-07 | 1.64E-06 |
| ACP7 | 1.767032439 | 2.64E-07 | 8.64E-07 |
| VTN | -1.931580987 | 1.93E-15 | 2.48E-14 |
