# Supplementary material for: Exploring the prognostic value of S100A11 and its association with immune infiltration in breast cancer
Source: Sci Rep. 2023 Dec 21;13:22922. doi: 10.1038/s41598-023-50160-x (PMC10739898; doi:10.1038/s41598-023-50160-x)
Supplement: Supplementary file 5 — Supplementary Table S4. [file 41598_2023_50160_MOESM5_ESM.docx]

**Supplementary Table S4 the common genes of the S100A11 co-expressed genes and S100A11-related DEGs**

| **Serial number** | **gene** |
| --- | --- |
| 1 | RN7SL674P |
| 2 | CHI3L2 |
| 3 | DCDC1 |
| 4 | TMPRSS6 |
| 5 | CWH43 |
| 6 | LINC02224 |
| 7 | AL356311.1 |
| 8 | AC096733.2 |
| 9 | CCL20 |
| 10 | CRABP1 |
| 11 | ADCY1 |
| 12 | NXPH3 |
| 13 | MAG |
| 14 | KCNC2 |
| 15 | SLPI |
| 16 | KISS1 |
| 17 | TMEM171 |
| 18 | LINC01133 |
| 19 | TBC1D9 |
| 20 | RAB39B |
| 21 | DPY19L2P4 |
| 22 | CASP14 |
| 23 | PTPRN2 |
| 24 | DEFB1 |
| 25 | S100A8 |
| 26 | IVL |
| 27 | AL021068.1 |
| 28 | SYN3-AS1 |
| 29 | THSD4 |
| 30 | NOVA1 |
| 31 | A2ML1 |
| 32 | KRT16 |
| 33 | PPP1R14C |
| 34 | AC103740.2 |
| 35 | AC064799.2 |
| 36 | VGLL1 |
| 37 | CLSTN2 |
| 38 | CALB2 |
| 39 | WDR17 |
| 40 | AC093297.2 |
| 41 | SRARP |
| 42 | ACADSB |
| 43 | TEX14 |
| 44 | AL445933.2 |
| 45 | IRAIN |
| 46 | AL136115.1 |
| 47 | ORM2 |
| 48 | AC092667.1 |
| 49 | TMEM26 |
| 50 | DUSP9 |
| 51 | CPA4 |
| 52 | ATP1A2 |
| 53 | AC125603.2 |
| 54 | HEPACAM2 |
| 55 | NKAIN1 |
| 56 | PIEZO2 |
| 57 | AGTR1 |
| 58 | GSDMC |
| 59 | ZBTB16 |
| 60 | ABCC8 |
| 61 | MAPT-IT1 |
| 62 | ELOVL2 |
| 63 | AC008663.1 |
| 64 | RN7SL381P |
| 65 | AL133387.1 |
| 66 | S100A2 |
| 67 | AL078582.2 |
| 68 | KCNG1 |
| 69 | S100A7 |
| 70 | SORCS1 |
| 71 | LINC01488 |
| 72 | EN1 |
| 73 | CTSV |
| 74 | CT62 |
| 75 | STK32B |
| 76 | PEX5L |
| 77 | SNORA11 |
| 78 | LCN2 |
| 79 | NEURL1 |
| 80 | GPR139 |
| 81 | WNK4 |
| 82 | AC036108.1 |
| 83 | RGS22 |
| 84 | BCL2 |
| 85 | SLURP1 |
| 86 | TPSG1 |
| 87 | GRIA1 |
| 88 | LYPD6 |
| 89 | CPB1 |
| 90 | KRT6A |
| 91 | LONRF2 |
| 92 | PHF21B |
| 93 | TNRC18P1 |
| 94 | CA9 |
| 95 | AC079296.1 |
| 96 | AC105328.1 |
| 97 | STC2 |
| 98 | AKR7A3 |
| 99 | RAET1L |
| 100 | MRPS30-DT |
| 101 | TPRG1 |
| 102 | RERG-AS1 |
| 103 | MYT1 |
| 104 | AC093297.1 |
| 105 | S100A9 |
| 106 | KRT81 |
| 107 | LBP |
| 108 | NAV3 |
| 109 | INSYN2A |
| 110 | DNAJC12 |
| 111 | KRT83 |
| 112 | AFF3 |
| 113 | AL353748.2 |
| 114 | NRXN3 |
| 115 | LINC01956 |
| 116 | IGF1R |
| 117 | NCCRP1 |
| 118 | ART3 |
| 119 | DACH1 |
| 120 | CADM2 |
| 121 | LRP2 |
| 122 | PHGR1 |
| 123 | TRH |
| 124 | FSIP1 |
| 125 | SPATA46 |
| 126 | KCNJ3 |
| 127 | FUT3 |
| 128 | IL20RB |
| 129 | SEZ6L |
| 130 | AC093838.1 |
| 131 | SBSN |
| 132 | TMEM145 |
| 133 | AC098679.5 |
| 134 | CST9 |
| 135 | SLC16A6 |
| 136 | SLC27A2 |
| 137 | AC061961.1 |
| 138 | UPK2 |
| 139 | SLC15A1 |
| 140 | CST9L |
| 141 | GAL |
| 142 | GRAMD4P8 |
| 143 | KCND3 |
| 144 | S100A7A |
| 145 | RERG-IT1 |
| 146 | RARRES1 |
| 147 | SEZ6 |
| 148 | CALML5 |
| 149 | VTN |
|  |  |
