# Supplementary material for: Exploring the prognostic value of S100A11 and its association with immune infiltration in breast cancer
Source: Sci Rep. 2023 Dec 21;13:22922. doi: 10.1038/s41598-023-50160-x (PMC10739898; doi:10.1038/s41598-023-50160-x)
Supplement: Supplementary file 6 — Supplementary Table S5. [file 41598_2023_50160_MOESM6_ESM.docx]

**Supplementary Table S5 The results of GO and KEGG enrichment analysis**

| **ONTOLOGY** | **ID** | **Description** | **GeneRatio** | **BgRatio** | **pvalue** | **p.adjust** | **qvalue** | **geneID** | **Count** |
| --- | --- | --- | --- | --- | --- | --- | --- | --- | --- |
| BP | GO:0019730 | antimicrobial humoral response | 7/102 | 126/18614 | 5.97E-06 | 0.00548012 | 0.004982343 | SLPI/DEFB1/S100A7/KRT6A/S100A9/CST9/CST9L | 7 |
| BP | GO:0031424 | keratinization | 6/102 | 83/18614 | 6.26E-06 | 0.00548012 | 0.004982343 | CASP14/IVL/KRT16/KRT6A/KRT81/KRT83 | 6 |
| BP | GO:0042742 | defense response to bacterium | 10/102 | 314/18614 | 8.75E-06 | 0.00548012 | 0.004982343 | CCL20/SLPI/DEFB1/S100A8/GSDMC/S100A7/LCN2/KRT6A/S100A9/LBP | 10 |
| BP | GO:0051238 | sequestering of metal ion | 4/102 | 26/18614 | 1.16E-05 | 0.00548012 | 0.004982343 | S100A8/S100A7/LCN2/S100A9 | 4 |
| BP | GO:0071805 | potassium ion transmembrane transport | 8/102 | 217/18614 | 2.57E-05 | 0.009729542 | 0.008845776 | KCNC2/ATP1A2/ABCC8/KCNG1/WNK4/KCNJ3/GAL/KCND3 | 8 |
| BP | GO:0008544 | epidermis development | 10/102 | 375/18614 | 4.01E-05 | 0.011381956 | 0.010348095 | CASP14/IVL/KRT16/S100A7/BCL2/KRT6A/KRT81/KRT83/GAL/CALML5 | 10 |
| BP | GO:0030216 | keratinocyte differentiation | 7/102 | 174/18614 | 4.84E-05 | 0.011381956 | 0.010348095 | CASP14/IVL/KRT16/S100A7/KRT6A/KRT81/KRT83 | 7 |
| BP | GO:0043588 | skin development | 9/102 | 308/18614 | 4.93E-05 | 0.011381956 | 0.010348095 | CASP14/IVL/KRT16/S100A7/BCL2/KRT6A/KRT81/KRT83/GAL | 9 |
| BP | GO:0006813 | potassium ion transport | 8/102 | 241/18614 | 5.41E-05 | 0.011381956 | 0.010348095 | KCNC2/ATP1A2/ABCC8/KCNG1/WNK4/KCNJ3/GAL/KCND3 | 8 |
| BP | GO:0006959 | humoral immune response | 8/102 | 250/18614 | 7.00E-05 | 0.013248474 | 0.012045072 | SLPI/DEFB1/S100A7/BCL2/KRT6A/S100A9/CST9/CST9L | 8 |
| BP | GO:0019935 | cyclic-nucleotide-mediated signaling | 5/102 | 85/18614 | 0.000103742 | 0.017853139 | 0.01623148 | ADCY1/KCNC2/DEFB1/PEX5L/GAL | 5 |
| BP | GO:0042886 | amide transport | 9/102 | 349/18614 | 0.000127891 | 0.01963758 | 0.017853835 | KISS1/PTPRN2/S100A8/ATP1A2/ABCC8/LRP2/TRH/SLC15A1/GAL | 9 |
| BP | GO:0002523 | leukocyte migration involved in inflammatory response | 3/102 | 19/18614 | 0.000145233 | 0.01963758 | 0.017853835 | S100A8/S100A9/LBP | 3 |
| BP | GO:0035930 | corticosteroid hormone secretion | 3/102 | 19/18614 | 0.000145233 | 0.01963758 | 0.017853835 | AGTR1/WNK4/GAL | 3 |
| BP | GO:0019933 | cAMP-mediated signaling | 4/102 | 52/18614 | 0.000187992 | 0.023724587 | 0.021569605 | ADCY1/DEFB1/PEX5L/GAL | 4 |
| BP | GO:0019932 | second-messenger-mediated signaling | 8/102 | 292/18614 | 0.000204136 | 0.024151882 | 0.021958088 | CCL20/ADCY1/KCNC2/DEFB1/ATP1A2/AGTR1/PEX5L/GAL | 8 |
| BP | GO:0060986 | endocrine hormone secretion | 4/102 | 56/18614 | 0.000250805 | 0.027927851 | 0.025391073 | KISS1/AGTR1/WNK4/GAL | 4 |
| BP | GO:0009913 | epidermal cell differentiation | 7/102 | 240/18614 | 0.000354485 | 0.037280052 | 0.033893784 | CASP14/IVL/KRT16/S100A7/KRT6A/KRT81/KRT83 | 7 |
| BP | GO:0035929 | steroid hormone secretion | 3/102 | 27/18614 | 0.000424659 | 0.042309426 | 0.038466322 | AGTR1/WNK4/GAL | 3 |
| BP | GO:1901018 | positive regulation of potassium ion transmembrane transporter activity | 3/102 | 29/18614 | 0.000526296 | 0.045941059 | 0.041768082 | KCNC2/ABCC8/GAL | 3 |
| BP | GO:0010959 | regulation of metal ion transport | 9/102 | 423/18614 | 0.000526985 | 0.045941059 | 0.041768082 | KCNC2/ATP1A2/NKAIN1/ABCC8/KCNG1/WNK4/BCL2/STC2/GAL | 9 |
| BP | GO:0015833 | peptide transport | 7/102 | 259/18614 | 0.000558986 | 0.045941059 | 0.041768082 | KISS1/PTPRN2/S100A8/ABCC8/TRH/SLC15A1/GAL | 7 |
| BP | GO:1901016 | regulation of potassium ion transmembrane transporter activity | 4/102 | 69/18614 | 0.000559046 | 0.045941059 | 0.041768082 | KCNC2/ABCC8/KCNG1/GAL | 4 |
| BP | GO:0099505 | regulation of presynaptic membrane potential | 3/102 | 30/18614 | 0.000582454 | 0.045941059 | 0.041768082 | KCNC2/GRIA1/KCNJ3 | 3 |
| BP | GO:0042391 | regulation of membrane potential | 9/102 | 433/18614 | 0.000622958 | 0.047170396 | 0.042885755 | KCNC2/ATP1A2/PIEZO2/BCL2/GRIA1/INSYN2A/KCNJ3/KCND3/SEZ6 | 9 |
| BP | GO:0045109 | intermediate filament organization | 4/102 | 73/18614 | 0.000692175 | 0.048529178 | 0.044121114 | KRT16/KRT6A/KRT81/KRT83 | 4 |
| BP | GO:0071260 | cellular response to mechanical stimulus | 4/102 | 73/18614 | 0.000692175 | 0.048529178 | 0.044121114 | MAG/ATP1A2/PIEZO2/IGF1R | 4 |
| BP | GO:0071621 | granulocyte chemotaxis | 5/102 | 131/18614 | 0.00077179 | 0.052178506 | 0.047438962 | CCL20/S100A8/S100A7/S100A9/LBP | 5 |
| CC | GO:0043025 | neuronal cell body | 12/106 | 500/19518 | 1.74E-05 | 0.00319547 | 0.002504459 | KCNC2/KISS1/ATP1A2/NEURL1/GRIA1/LYPD6/IGF1R/CADM2/SEZ6L/GAL/KCND3/SEZ6 | 12 |
| CC | GO:0034705 | potassium channel complex | 5/106 | 89/19518 | 0.000124163 | 0.011423014 | 0.00895282 | KCNC2/ABCC8/KCNG1/KCNJ3/KCND3 | 5 |
| CC | GO:0045095 | keratin filament | 5/106 | 97/19518 | 0.000186275 | 0.011424886 | 0.008954287 | CASP14/KRT16/KRT6A/KRT81/KRT83 | 5 |
| CC | GO:0008076 | voltage-gated potassium channel complex | 4/106 | 78/19518 | 0.000861018 | 0.032398554 | 0.025392459 | KCNC2/KCNG1/KCNJ3/KCND3 | 4 |
| CC | GO:0042383 | sarcolemma | 5/106 | 136/19518 | 0.000880395 | 0.032398554 | 0.025392459 | ATP1A2/ABCC8/IGF1R/KCNJ3/KCND3 | 5 |
| CC | GO:0045111 | intermediate filament cytoskeleton | 6/106 | 253/19518 | 0.002590646 | 0.063181288 | 0.049518515 | CASP14/S100A8/KRT16/KRT6A/KRT81/KRT83 | 6 |
| CC | GO:0030315 | T-tubule | 3/106 | 52/19518 | 0.002836219 | 0.063181288 | 0.049518515 | ATP1A2/IGF1R/KCNJ3 | 3 |
| CC | GO:0097440 | apical dendrite | 2/106 | 15/19518 | 0.002929491 | 0.063181288 | 0.049518515 | NEURL1/SEZ6 | 2 |
| CC | GO:0034703 | cation channel complex | 5/106 | 192/19518 | 0.003975307 | 0.063181288 | 0.049518515 | KCNC2/ABCC8/KCNG1/KCNJ3/KCND3 | 5 |
| CC | GO:0001533 | cornified envelope | 3/106 | 59/19518 | 0.004059181 | 0.063181288 | 0.049518515 | CASP14/IVL/KRT16 | 3 |
| CC | GO:0090533 | cation-transporting ATPase complex | 2/106 | 18/19518 | 0.004223612 | 0.063181288 | 0.049518515 | ATP1A2/ABCC8 | 2 |
| CC | GO:0035580 | specific granule lumen | 3/106 | 62/19518 | 0.004667208 | 0.063181288 | 0.049518515 | SLPI/ORM2/LCN2 | 3 |
| CC | GO:0098688 | parallel fiber to Purkinje cell synapse | 2/106 | 19/19518 | 0.004703847 | 0.063181288 | 0.049518515 | CALB2/KCNJ3 | 2 |
| CC | GO:0060076 | excitatory synapse | 3/106 | 64/19518 | 0.005101515 | 0.063181288 | 0.049518515 | CALB2/GRIA1/KCNJ3 | 3 |
| CC | GO:0099634 | postsynaptic specialization membrane | 4/106 | 129/19518 | 0.005382971 | 0.063181288 | 0.049518515 | ADCY1/CLSTN2/GRIA1/KCND3 | 4 |
| CC | GO:1902495 | transmembrane transporter complex | 7/106 | 392/19518 | 0.005557252 | 0.063181288 | 0.049518515 | KCNC2/ATP1A2/ABCC8/KCNG1/GRIA1/KCNJ3/KCND3 | 7 |
| CC | GO:0034702 | ion channel complex | 6/106 | 299/19518 | 0.005837402 | 0.063181288 | 0.049518515 | KCNC2/ABCC8/KCNG1/GRIA1/KCNJ3/KCND3 | 6 |
| MF | GO:0050786 | RAGE receptor binding | 3/102 | 10/18369 | 1.94E-05 | 0.005429607 | 0.004531476 | S100A8/S100A7/S100A9 | 3 |
| MF | GO:0005249 | voltage-gated potassium channel activity | 5/102 | 88/18369 | 0.000130058 | 0.018208146 | 0.015196272 | KCNC2/ABCC8/KCNG1/KCNJ3/KCND3 | 5 |
| MF | GO:0015079 | potassium ion transmembrane transporter activity | 6/102 | 157/18369 | 0.00024271 | 0.022652907 | 0.018905809 | KCNC2/ATP1A2/ABCC8/KCNG1/KCNJ3/KCND3 | 6 |
| MF | GO:0005267 | potassium channel activity | 5/102 | 121/18369 | 0.00057127 | 0.039988869 | 0.033374169 | KCNC2/ABCC8/KCNG1/KCNJ3/KCND3 | 5 |
| MF | GO:0030280 | structural constituent of skin epidermis | 3/102 | 36/18369 | 0.001038941 | 0.040047141 | 0.033422802 | KRT6A/KRT81/KRT83 | 3 |
| MF | GO:0022843 | voltage-gated monoatomic cation channel activity | 5/102 | 140/18369 | 0.001102919 | 0.040047141 | 0.033422802 | KCNC2/ABCC8/KCNG1/KCNJ3/KCND3 | 5 |
| MF | GO:0016248 | channel inhibitor activity | 3/102 | 41/18369 | 0.001520284 | 0.040047141 | 0.033422802 | ABCC8/WNK4/BCL2 | 3 |
| MF | GO:0031731 | CCR6 chemokine receptor binding | 2/102 | 11/18369 | 0.001625352 | 0.040047141 | 0.033422802 | CCL20/DEFB1 | 2 |
| MF | GO:0099508 | voltage-gated monoatomic ion channel activity involved in regulation of presynaptic membrane potential | 2/102 | 11/18369 | 0.001625352 | 0.040047141 | 0.033422802 | KCNC2/KCNJ3 | 2 |
| MF | GO:0022836 | gated channel activity | 7/102 | 308/18369 | 0.00165197 | 0.040047141 | 0.033422802 | KCNC2/PIEZO2/ABCC8/KCNG1/GRIA1/KCNJ3/KCND3 | 7 |
| MF | GO:0022839 | monoatomic ion gated channel activity | 7/102 | 308/18369 | 0.00165197 | 0.040047141 | 0.033422802 | KCNC2/PIEZO2/ABCC8/KCNG1/GRIA1/KCNJ3/KCND3 | 7 |
| MF | GO:0061134 | peptidase regulator activity | 6/102 | 230/18369 | 0.001788955 | 0.040047141 | 0.033422802 | SLPI/A2ML1/CTSV/CST9/CST9L/RARRES1 | 6 |
| MF | GO:0008188 | neuropeptide receptor activity | 3/102 | 47/18369 | 0.002257503 | 0.040047141 | 0.033422802 | SORCS1/GPR139/GAL | 3 |
| MF | GO:0031994 | insulin-like growth factor I binding | 2/102 | 13/18369 | 0.002288408 | 0.040047141 | 0.033422802 | IGF1R/LRP2 | 2 |
| MF | GO:0035325 | Toll-like receptor binding | 2/102 | 13/18369 | 0.002288408 | 0.040047141 | 0.033422802 | S100A8/S100A9 | 2 |
| MF | GO:1902282 | voltage-gated potassium channel activity involved in ventricular cardiac muscle cell action potential repolarization | 2/102 | 13/18369 | 0.002288408 | 0.040047141 | 0.033422802 | KCNJ3/KCND3 | 2 |
| MF | GO:0005504 | fatty acid binding | 3/102 | 49/18369 | 0.002544564 | 0.041910469 | 0.03497791 | CRABP1/S100A8/S100A9 | 3 |
| MF | GO:0004866 | endopeptidase inhibitor activity | 5/102 | 173/18369 | 0.002791335 | 0.043420763 | 0.036238381 | SLPI/A2ML1/CST9/CST9L/RARRES1 | 5 |
| MF | GO:0030414 | peptidase inhibitor activity | 5/102 | 180/18369 | 0.003309312 | 0.044324263 | 0.03699243 | SLPI/A2ML1/CST9/CST9L/RARRES1 | 5 |
| MF | GO:0030548 | acetylcholine receptor regulator activity | 2/102 | 16/18369 | 0.003482621 | 0.044324263 | 0.03699243 | SLURP1/LYPD6 | 2 |
| MF | GO:0086008 | voltage-gated potassium channel activity involved in cardiac muscle cell action potential repolarization | 2/102 | 16/18369 | 0.003482621 | 0.044324263 | 0.03699243 | KCNJ3/KCND3 | 2 |
| MF | GO:0099602 | neurotransmitter receptor regulator activity | 2/102 | 16/18369 | 0.003482621 | 0.044324263 | 0.03699243 | SLURP1/LYPD6 | 2 |
| MF | GO:0061135 | endopeptidase regulator activity | 5/102 | 187/18369 | 0.003893247 | 0.045615681 | 0.03807023 | SLPI/A2ML1/CST9/CST9L/RARRES1 | 5 |
| MF | GO:0005244 | voltage-gated monoatomic ion channel activity | 5/102 | 189/18369 | 0.004072829 | 0.045615681 | 0.03807023 | KCNC2/ABCC8/KCNG1/KCNJ3/KCND3 | 5 |
| MF | GO:0022832 | voltage-gated channel activity | 5/102 | 189/18369 | 0.004072829 | 0.045615681 | 0.03807023 | KCNC2/ABCC8/KCNG1/KCNJ3/KCND3 | 5 |
| MF | GO:0005520 | insulin-like growth factor binding | 2/102 | 19/18369 | 0.004909227 | 0.050324189 | 0.041999887 | IGF1R/LRP2 | 2 |
| MF | GO:0036041 | long-chain fatty acid binding | 2/102 | 19/18369 | 0.004909227 | 0.050324189 | 0.041999887 | S100A8/S100A9 | 2 |
| MF | GO:0015267 | channel activity | 8/102 | 478/18369 | 0.005148276 | 0.050324189 | 0.041999887 | KCNC2/PIEZO2/ABCC8/KCNG1/BCL2/GRIA1/KCNJ3/KCND3 | 8 |
| MF | GO:0022803 | passive transmembrane transporter activity | 8/102 | 479/18369 | 0.005212148 | 0.050324189 | 0.041999887 | KCNC2/PIEZO2/ABCC8/KCNG1/BCL2/GRIA1/KCNJ3/KCND3 | 8 |
| KEGG | hsa04657 | IL-17 signaling pathway | 6/45 | 94/8579 | 8.51E-06 | 0.001386318 | 0.001163844 | CCL20/S100A8/S100A7/LCN2/S100A9/S100A7A | 6 |
| KEGG | hsa04915 | Estrogen signaling pathway | 5/45 | 137/8579 | 0.000706002 | 0.054323598 | 0.045605862 | ADCY1/KRT16/BCL2/KCNJ3/CALML5 | 5 |
| KEGG | hsa04261 | Adrenergic signaling in cardiomyocytes | 5/45 | 154/8579 | 0.001195929 | 0.054323598 | 0.045605862 | ADCY1/ATP1A2/AGTR1/BCL2/CALML5 | 5 |
| KEGG | hsa04713 | Circadian entrainment | 4/45 | 97/8579 | 0.001604449 | 0.054323598 | 0.045605862 | ADCY1/GRIA1/KCNJ3/CALML5 | 4 |
| KEGG | hsa04925 | Aldosterone synthesis and secretion | 4/45 | 98/8579 | 0.001666368 | 0.054323598 | 0.045605862 | ADCY1/ATP1A2/AGTR1/CALML5 | 4 |
